# Two-dimensional melt growth of large-scale, single-crystalline hybrid organic-inorganic perovskite films

**DOI:** 10.1038/s41467-026-73886-4

**Published:** 2026-06-03

**Authors:** Yuanyuan Jin, Gang Wang, Qiye Guan, Yixin Li, Tae Joo Shin, Seulyi Lee, Tingting Li, Song Liu, Guankui Long, Philip C. Y. Chow, Yongqing Cai, Kian Ping Loh, Junhao Lin, Kai Leng

**Affiliations:** 1 Department of Applied Physics, The Hong Kong Polytechnic University, Hong Kong, China; 2 State Key Laboratory of Quantum Functional Materials, Department of Physics, Guangdong Basic Research Center of Excellence for Quantum Science, Southern University of Science and Technology (SUSTech), Shenzhen, China; 3 Institute of Applied Physics and Materials Engineering, University of Macau, Taipa, Macau, China; 4 Graduate School of Semiconductor Materials and Devices Engineering, Ulsan National Institute of Science and Technology (UNIST), Ulsan, Republic of Korea; 5 Office of Research Facilities and Training, Ulsan National Institute of Science and Technology (UNIST), Ulsan, Republic of Korea; 6 College of Chemistry and Chemical Engineering, Hunan University, Changsha, China; 7 National Key Laboratory of Semiconductor Laser, School of Materials Science and Engineering, Nankai University, Tianjin, China; 8 Department of Mechanical Engineering, The University of Hong Kong, Hong Kong, China; 9 Department of Chemistry, National University of Singapore, Singapore, Singapore; 10 Quantum Science Center of Guangdong-Hong Kong-Macao Greater Bay Area (Guangdong), Shenzhen, China

**Keywords:** Two-dimensional materials, Organic-inorganic nanostructures

## Abstract

Melt growth is a process for creating large, bulk single crystals by solidifying a molten material. It combines elements of the Czochralski method, which creates a molten phase, and the Bridgman method, which controls the temperature gradient. Here, we apply two-dimensional (2D) melt growth to synthesize large-scale, single-crystal hybrid organic-inorganic perovskites (HOIPs), enabling substrate-agnostic crystallization with precise thickness control. Our method involves a vapor-liquid-solid process, where the reaction between the pre-deposited inorganic Na_x_PbBr_y_ seeding layer and the organic precursor flux produces the 2D molten phase of HOIPs. This molten phase spreads into a 2D liquid film and allows uniform, large-scale crystallization of ultrathin HOIPs in a substrate-agnostic manner, bypassing requirements for lattice matching. Using this approach, we successfully grow 2D (*n* = 1) and quasi-2D (*n* > 1) ferroelectric HOIP films on SiO_2_/Si wafers at a low thermal budget, enabling direct large-scale device fabrication. Statistical analysis of devices demonstrates reliable ferroelectric switching and uniform electronic performance across the film. Our method holds great potential for other types of HOIPs and heterostructures, paving the way for applications in large-scale on-chip devices.

## Abstract (web summary)

Researchers have developed a two-dimensional melt-growth method to produce large-area, ultrathin single-crystal hybrid perovskite films independent of substrate type, thereby enabling the scalable fabrication of reliable ferroelectric devices.

## Introduction

The scalable growth of uniform, high-quality HOIP films with controllable thickness remains a significant challenge in perovskite research, which is crucial for device performance and industrial applications^1–12^. Traditionally, the large-scale growth of single-crystalline films typically relies on lattice matching between the material and the substrate^13,14^. However, growing single-crystalline films on amorphous substrates has consistently been challenging due to the lack of orientational alignment between the grains and the substrate. Epitaxial growth, a method widely used for inorganic transition metal dichalcogenide (TMD) materials^15,16^, presents significant challenges for HOIPs due to the absence of lattice-matching substrates. Although vapor-phase growth of HOIPs has been explored on crystalline substrates such as mica and sapphire, the resulting single-crystalline domains are typically limited to micro-scale sizes with inconsistent thicknesses^17–19^. To date, centimetre-scale single-crystalline three-dimensional (3D) MAPbI_3_ films have only been achieved by homoepitaxy on MAPbI_3_ substrate^10^. Meanwhile, lateral heteroepitaxy of 2D HOIP heterostructures (e.g., (PEA)_2_PbI_4_/(PEA)_2_PbBr_4_) produced micrometer-scale single-crystalline domains^20,21^. However, there is a lack of reports on the growth of large-area (e.g., >1 cm^2^) single-crystalline HOIP films on hetero-substrate or amorphous substrate. Furthermore, regardless of substrate, quasi-2D HOIPs (*n* > 1) are challenging to grow due to thermodynamic instability and phase segregation between lower-*n* and higher-*n* homologues^1^. Addressing these challenges is critical, as *n* > 1 HOIPs exhibit superior performance in optoelectronic and quantum devices^9,22^.

Recently, the 2D Czochralski method has been demonstrated to grow centimetre-scale inorganic single-crystal MoS_2_ on glass^23^. In this method, the eutectic phase formation between pre-deposited MoO_3_ powder and Na_2_O from the glass substrate is critical in creating a 2D liquid precursor film on the molten glass substrate at 1100 °C, which allows 2D crystallization. However, a further transfer step is needed to delaminate the MoS_2_ from glass onto SiO_2_/Si for device fabrication. Compared to TMDs, HOIPs present complex elemental composition, and their structures consist of ionic lattices and soft hybrid interfaces. Here, we apply 2D melt growth, which combines the elements of 2D Czochralski and Bridgman methods^24,25^, to successfully grow single-crystal HOIPs with desired thickness on SiO_2_/Si wafer at CMOS-compatible temperature. Directly growing HOIPs on SiO_2_/Si eliminates the transfer process and enables wafer-scale integration with silicon-based photonics^2,4,26,27^. HOIPs exhibit glass transition temperatures (< 250 °C) well below their decomposition thresholds, potentially allowing melt-growth from the molten state^28,29^. However, simply heating pre-coated HOIP solids to their molten state, followed by conventional melt crystallization, a bulk crystal growth technique, fails to produce large-scale single-crystalline films. For example, blade-coating molten 2D HOIPs on amorphous FTO substrates yields polycrystalline domains^30^. To achieve substrate-agnostic growth of single-crystalline HOIP films via 2D melt growth, two critical criteria must be fulfilled. First, it is essential to achieve large-area congruent melting of both organic and inorganic HOIP precursors. Second, the molten phase must fully wet the substrate to form a stable, continuous 2D liquid film. In such a process, the mobility of ions or molecules in the liquid film allows reorganization driven by intrinsic bonding interactions rather than substrate forces. HOIPs with their strong in-plane covalent or ionic bonds, as well as dangling bond-free surfaces, are particularly favorable for substrate-agnostic crystallization^31^. Additionally, interfacial forces also offer pathways to achieve order in the grown layer^32,33^. The formation of a 2D liquid film requires minimizing interfacial energy between the molten phase of HOIPs and the substrate.

In this study, we demonstrate a seeding-assisted 2D melt growth of single-crystal HOIPs by leveraging the reaction of NaOH with PbBr_2_ to form the Na_*x*_PbBr_*y*_ seeding layer, which is the key to reducing interfacial energy during 2D melt growth. Exposure of Na_*x*_PbBr_*y*_ to the organic vapor precursor initiates a melt reaction at a substrate temperature of 210 °C, and the resulting molten phase rapidly spreads across the entire substrate. In contrast, using a conventional PbBr_2_ inorganic precursor for HOIPs growth fails to produce a large-area liquid film. This strategy enables control over film thickness and crystallographic orientation while ensuring structural uniformity across wafer-scale substrates. Using this method, we successfully grow single-crystalline films of (BA)_2_PbBr_4_ (*n* = 1), (BA)_2_PbI_4_ (*n* = 1), (HA)_2_PbBr_4_ (*n* = 1), (HA)_2_(MA)Pb_2_Br_7_ (*n* = 2) and ferroelectric (BA)_2_(MA)Pb_2_Br_7_ (*n* = 2), (BA)_2_(MA)_2_Pb_3_Br_10_ (*n* = 3) on SiO_2_/Si substrates, demonstrating the versatility of this approach. To confirm the centimetre-scale single-crystalline nature of the grown HOIP films, synchrotron-based grazing-incidence X-ray diffraction (GIXRD) measurements were conducted. Additionally, cross-sectional high-resolution transmission electron microscopy (HRTEM) verified the substrate-agnostic crystallization of the 2D HOIP films with the underlying SiO_2_/Si. This scalable method provides a promising pathway for the tailored synthesis of thin-film HOIPs, enabling large-scale integration with advanced device applications.

## Results

### 2D melt growth of centimetre-scale HOIPs

**Fig. 1:**
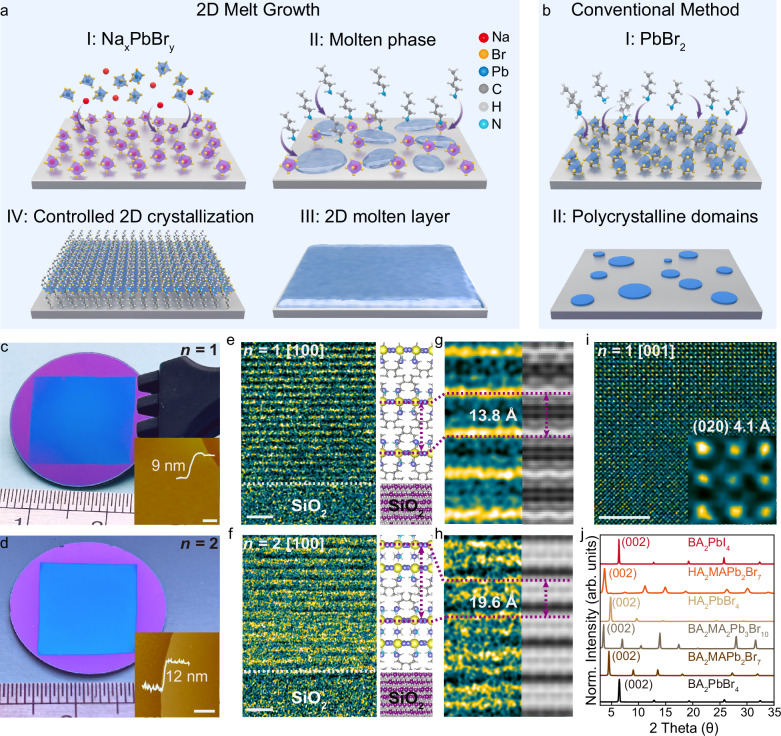
Seeding-assisted 2D melt growth of large-scale, single-crystal HOIPs. **a** Schematic illustration of the 2D melt growth process of HOIPs via CVD. **b** Schematic depiction of conventional CVD growth. **c**, **d** Photographs of centimetre-scale, single-crystalline films of *n* = 1 (BA)_2_PbBr_4_ with thickness of 9 nm (**c**) and *n* = 2 (BA)_2_(MA)Pb_2_Br_7_ with thickness of 12 nm (**d**) grown on SiO_2_/Si. Insets: AFM images of the corresponding films. **e**, **f** Cross-sectional cryogenic HRTEM images of *n* = 1 (**e**) and *n* = 2 (**f**) films on SiO_2_/Si, with corresponding structural models displayed alongside each image. **g**, **h** High-magnification cross-sectional HRTEM images of *n* = 1 (**g**) and *n* = 2 (**h**) films, highlighting alternating bright (inorganic) and dark (organic) layers. The left side shows the experimental results, while the right side presents the simulation results. **i** Cryogenic HRTEM images of *n* = 1 (BA)_2_PbBr_4_ film viewed along the [001] direction. **j** XRD spectra of six different crystalline HOIP films by 2D melt growth. Scale bars, 1 µm (inset in **c**), 3 µm (inset in **d**), 5 nm (**e**, **f**, **i**). Source data are provided as a Source Data file.

The 2D melt growth involves four stages (Fig. 1a). In stage I, pre-mixed NaOH and PbBr_2_ powders are co-evaporated onto SiO_2_/Si via chemical vapor deposition (CVD) to form a uniform Na_*x*_PbBr_*y*_ (*x* < 1) layer (Supplementary Fig. 1a), as confirmed by subsequent stoichiometric analysis and structural characterization. Among the Na_*x*_PbBr_*y*_ formed, NaPb_3_Br_7_ is the most frequently observed composition, thus, a representative reaction is described by Eq. (1):

1 $$2{{{\rm{NaOH}}}}+7{{{{\rm{PbBr}}}}}_{2}\to {2{{{\rm{NaPb}}}}}_{3}{{{{\rm{Br}}}}}_{7}+{{{{\rm{Pb}}}}\left({{{\rm{OH}}}}\right)}_{2}$$

At stage II, exposure of the Na_*x*_PbBr_*y*_ to organic vapor triggers a solid-to-liquid phase transition, forming the molten phase of (BA)_2_PbBr_4_. The molten phase spread uniformly across the SiO_2_/Si surface, forming a 2D molten layer that suppresses vertical crystallization (stage III). Subsequent controlled cooling of the molten layer enables the 2D crystallization of single-crystalline HOIP films (stage IV). The conversion reaction is described by Eq. (2):

2 $${{{{\rm{NaPb}}}}}_{3}{{{{\rm{Br}}}}}_{7}+6{{{\rm{BABr}}}}\to {3({{{\rm{BA}}}})}_{2}{{{{\rm{PbBr}}}}}_{4}+{{{\rm{NaBr}}}}$$

Our 2D melt growth fundamentally differs from conventional vapor-to-solid growth of HOIPs (Fig. 1b), and the latter produces only micro-scale domains^34^.

Figure 1c, d show representative photographs of centimetre-scale single-crystalline films of *n* = 1 (BA)_2_PbBr_4_ and *n* = 2 (BA)_2_(MA)Pb_2_Br_7_ grown directly on SiO_2_/Si. The atomic force microscopy (AFM) images (Fig. 1c, d, insets) reveal boundary-free surfaces with thicknesses of 9 nm and 12 nm, respectively. Significantly, cross-sectional HRTEM images of the *n* = 1 and *n* = 2 films (Fig. 1e, f) verify basal-plane-oriented growth of HOIP films, even on amorphous SiO_2_/Si, facilitated by strain buffering provided by vertically aligned organic cations. In the *n* = 1 film, inorganic [PbBr_4_]^2^⁻ octahedral layers, highlighted in yellow, alternated with interdigitated BA⁺ gaps, resulting in a periodicity of 13.8 Å (Fig. 1g). In contrast, the *n* = 2 displayed an increased periodicity of 19.6 Å, attributed to the thicker inorganic unit, as highlighted in yellow (Fig. 1h). This quasi-epitaxial growth leverages the strong covalent and ionic bonds within the inorganic quantum wells of 2D HOIPs, along with van der Waals forces between the organic spacers, overcoming the limitation of the substrate. In-plane HRTEM further confirms orthorhombic structure of *n* = 1 (Fig. 1i), with bright dots corresponding to Pb, Br, and BA columns along the [001] projection and dark dots representing equatorial Br columns along [001] (Supplementary Fig. 1b–f). Similarly, the *n* = 2 film also exhibits an orthorhombic structure akin to the *n* = 1 but with expanded unit cells and distinct MA incorporation (Supplementary Fig. 2). Additionally, we also grew (BA)_2_(MA)_2_Pb_3_Br_10_ (*n* = 3), (HA)_2_PbBr_4_ (*n* = 1), (HA)_2_(MA)Pb_2_Br_7_ (*n* = 2) and Pb-I based (BA)_2_PbI_4_ (*n* = 1) large-scale films on SiO_2_/Si, as shown in Supplementary Figs. 3–6. X-ray diffraction (XRD) patterns of six different HOIPs in Fig. 1j indicate the growth of highly oriented (001)-textured films^35^. Raman and photoluminescence mappings conducted confirm the uniformity of the grown HOIP films (Supplementary Fig. 7). It is worth noting that during the HOIP growth process, the by‑product Pb(OH)_2_ further decomposes into PbO in the crucible of the CVD furnace. Another by‑product, NaBr, is primarily detected at the edges of the as‑grown HOIP films (Supplementary Fig. 8) and can be readily removed using a DMF‑soaked cotton swab.

### Verification of large-area single-crystallinity

**Fig. 2:**
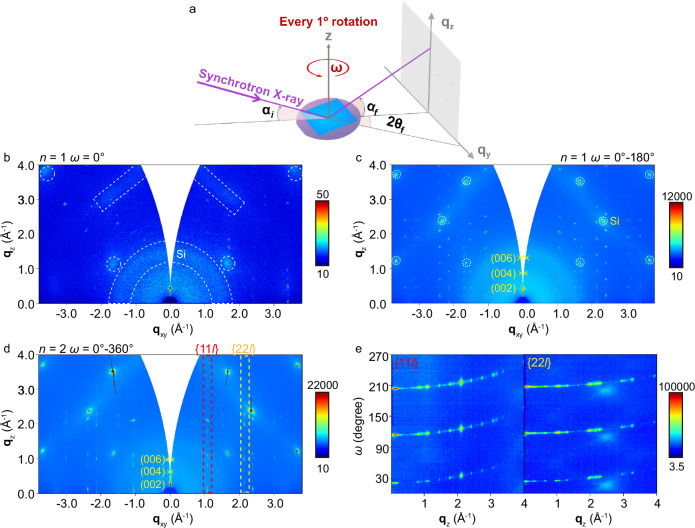
Verification of the large-area single-crystallinity of grown HOIP films. **a** Schematic of the synchrotron-based *ω*-scan GIXRD measurement performed on the grown HOIP films. **b** GIXRD pattern of the *n* = 1 film measured at *ω* = 0°. The diffuse scattering in the regions outlined by the white dashed line is due to the Si substrate. **c** Summed *ω*-dependent (0°−180°) GIXRD pattern of the *n* = 1 film, obtained by rotating the sample in-plane at 1° intervals. The diffractions circled in white dotted circles are due to the Si substrate. **d** Summed *ω*-dependent (0°−360°) GIXRD pattern of the *n* = 2 film, with the {11 *l*} and {22 *l*} diffraction spots highlighted in red and yellow, respectively. **e**, **q**_z_ vs *ω* results derived from the {11 *l*} (left) and {22 *l*} (right) diffraction spots shown in (**d**). Source data are provided as a Source Data file.

Synchrotron-based in-plane *ω*-scan GIXRD was performed to assess the crystallinity of the films over a large area (see “Methods” section). *ω* represents the in-plane rotation angle of the sample while maintaining a constant X-ray incidence direction (Fig. 2a). For the *n* = 1 film on SiO_2_/Si, the *ω* = 0° GIXRD pattern exhibits a limited number of diffraction spots (Fig. 2b). This is a characteristic of a single-crystalline film, where the reciprocal lattice points are discrete and periodic. In contrast, a polycrystalline film with random crystal orientations would exhibit numerous diffraction spots or diffraction rings across the Ewald sphere, even at a single *ω* angle^36,37^. Due to the thinness of the 2D HOIP film, diffuse diffraction spots originating from the SiO_2_/Si substrate were observed, indicated as the white circles (Fig. 2b). Crystallographic analysis via *ω*-dependent GIXRD (0°–180° for *n* = 1; 0°−360° for *n* = 2) confirmed single-crystalline order (Fig. 2c, d). Sample rotation enabled reciprocal lattice points to intersect the Ewald sphere, generating symmetric diffraction spots around **q**_*xy*_ = 0 Å^–1^. For *n* = 1, all measured spots except those from Si (Fig. 2c, white circles) matched the simulation result (Supplementary Fig. 9a), confirming an orthorhombic structure (*a* = 8.268 Å, *b* = 8.385 Å, *c* = 27.7 Å, space group *Pbca*), consistent with single-crystalline (BA)_2_PbBr_4_. Similarly, *n* = 2 (BA)_2_(MA)Pb_2_Br_7_ exhibited an orthorhombic lattice (*a* = 8.4 Å, *b* = 8.35 Å, *c* = 39.35 Å, *Cmc*2_1_), validated by simulated and measured spots alignment (Supplementary Fig. 9b and Supplementary Table 1). Furthermore, the **q**_z_ vs *ω* plot from *n* = 2 {11*l*} and {22*l*} spots (Fig. 2e) showed discrete 90°-spaced features, reflecting pseudo-tetragonal symmetry due to near-identical *a* and *b* parameters. Uniform spot spacing, without splitting or overlap, confirmed single crystallinity, excluding twinning effects (Supplementary Fig. 10).

### Mechanism of seeding-assisted 2D melt growth

**Fig. 3:**
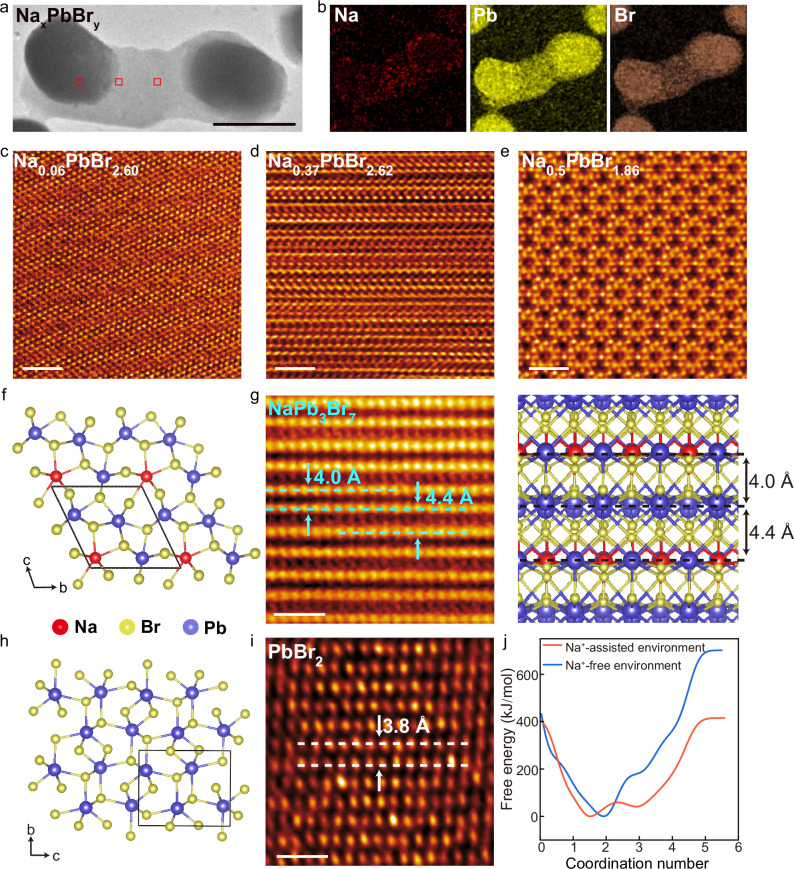
Structural characterization of Na_*x*_PbBr_*y*_ (*x* < 1) seeding phases. **a** Typical TEM morphology of Na_x_PbBr_y_, showing a transformation from particle-like to film-like morphology as Na^+^ is alloyed with PbBr_2_. **b** Corresponding EDS elemental mapping of Na, Pb, and Br in (**a**). **c**–**e** HRTEM images of three typical phases with sub-stoichiometric compositions: Na_0.06_PbBr_2.60_ (**c**), Na_0.37_PbBr_2.62_ (**d**), and Na_0.5_PbBr_1.86_ (**e**), corresponding to the red-boxed regions in (**a**) from left to right, respectively. **f**, Resolved atomic structure of NaPb_3_Br_7_ along the *a*-axis, obt*a*ined via 3D electron diffraction measurement of Na_0.37_PbBr_2.62_. **g** Atomic-resolution ADF-STEM image of NaPb_3_Br_7_ along the [102] direction, consistent with the atomic model shown on the right. **h** Atomic structure model of PbBr_2_ along the *a*-axis. **i** HRTEM image of PbBr_2_ along the [123] direction. **j** Free energy plots for Na^+^-assisted environment compared to Na^+^-free environment for the HOIP octahedron formation. Scale bars, 0.5 µm (**a**), 2 nm (**c**–**e**), 1 nm (**g**, **i**). Source data are provided as a Source Data file.

The alloying of PbBr_2_ with a small stoichiometric amount of NaOH is the key to preparing Na_*x*_PbBr_*y*_ (*x* < 1) seeding phases. Figure 3a shows the typical TEM image of the interface between PbBr_2_-rich zone (dark contrast) and intermediate Na_*x*_PbBr_*y*_ zone (lighter contrast), with the corresponding energy dispersive spectrometer (EDS) elemental mapping shown in Fig. 3b. The amalgamation of PbBr_2_ with Na^+^ results in a range of sub-stoichiometric alloyed phases as revealed in Fig. 3c–e. The identified sub-stoichiometric phases are Na_0.06_PbBr_2.60_, Na_0.37_PbBr_2.62_, and Na_0.5_PbBr_1.86_ (Supplementary Fig. 11), corresponding to the red box regions shown in Fig. 3a from left to right, respectively. Notably, as the Na^+^ content increases, the structure transitions from quasi-3D PbBr_2_ to 2D Na_*x*_PbBr_*y*_ (*x* < 1). AFM and scanning electron microscopy (SEM) characterizations further confirm the high density and 2D morphology of Na_*x*_PbBr_*y*_ as compared to PbBr_2_ (Supplementary Fig. 12).

We selected Na_0.37_PbBr_2.62_ as a representative and refined its stoichiometry to NaPb_3_Br_7_. A 3D electron diffraction resolved the crystal cell structure of NaPb_3_Br_7_ as: *a* = 4.31 Å, *b* = 9.23 Å, *c* = 9.34 Å, α = 111.27°, β = 103.92°, γ = 103.21° (Supplementary Fig. 13). In NaPb_3_Br_7_, Na^+^ ions (red) substitute Pb^2+^ sites in PbBr_2_ (Fig. 3f), inducing lattice contraction. This substitution ultimately distorts PbBr_2_’s orthorhombic framework into a triclinic structure. Atomic-resolution annular dark-field-scanning transmission electron microscopy (ADF-STEM) image of NaPb_3_Br_7_ revealed a layered configuration, with alternating Pb and Br rows (Fig. 3g). The repeating unit consists of three Pb rows, with Na^+^ occupation (intra-row distances: 4.0 Å and 4.4 Å), which differ from 3D PbBr_2_ (Fig. 3h, i). The layered structure of NaPb_3_Br_7_ demonstrates a stronger propensity for forming HOIPs compared to PbBr_2_. Molecular dynamic simulations reveal that the free energy of HOIP octahedron formation is lower in the Na^+^-assisted environment compared to the Na^+^-free environment (Fig. 3j and Supplementary Data 1–4). Besides the free energy consideration, structural factors also provide an additional explanation for why the Na^+^-assisted environment facilitates the conversion to 6-coordinate PbBr_6_ octahedra in HOIPs. The 3D PbBr_2_ crystallizes in the orthorhombic *Pnma* space group and adopts a cotunnite structure, where each Pb^2+^ is bonded in a 7-coordinate geometry to Br^−^. Upon substitution of Na^+^ in PbBr_2_ and its subsequent elimination as NaBr, the concentration of Br^-^ available to coordinate with Pb^2+^ decreases, thereby promoting the formation of 6-coordinate PbBr_6_ octahedra in HOIP. The formation enthalpies (Δ*H*) were calculated as −2.05 eV for Na^+^-assisted reaction (Eq. 3) and −1.43 eV for Na^+^-free reaction (Eq. 4), indicating that the seeding-assisted reaction is thermodynamically more favorable for producing the HOIPs.

3 $$\begin{array}{c}\frac{1}{3}{{{\rm{NaOH}}}}+\frac{7}{6}{{{\rm{PbB}}}}{{{{\rm{r}}}}}_{2}+2{{{\rm{BABr}}}}\to {{{\rm{B}}}}{{{{\rm{A}}}}}_{2}{{{\rm{PbB}}}}{{{{\rm{r}}}}}_{4}+\frac{1}{3}{{{\rm{NaBr}}}}+\frac{1}{6}{{{\rm{Pb}}}}{\left({{{\rm{OH}}}}\right)}_{2}\\ \triangle H=-2.05{{{\rm{eV}}}}\end{array}$$

4 $$2{{{\rm{BABr}}}}+{{{\rm{PbB}}}}{{{{\rm{r}}}}}_{2}\to {{{\rm{B}}}}{{{{\rm{A}}}}}_{2}{{{\rm{PbB}}}}{{{{\rm{r}}}}}_{4},\,\triangle H=-1.43{{{\rm{eV}}}}$$

**Fig. 4:**
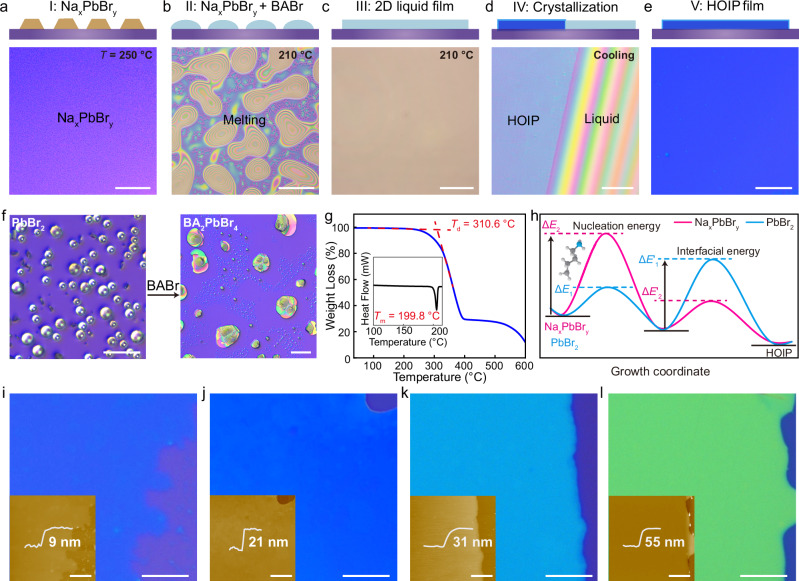
Mechanism of 2D melt growth. **a** Optical image of deposited Na_*x*_PbBr_*y*_ nanoparticles on SiO_2_/Si. **b** Congruent melting induced by BA^+^ vapor interacting with Na_*x*_PbBr_*y*_. **c** Formation of a uniform 2D molten layer. **d** 2D homogenous crystallization into HOIP film by cooling. **e** Optical image of as-grown BA_2_PbBr_4_ film on SiO_2_/Si. **f** Conventional CVD growth of HOIP using PbBr_2_. **g** TGA analysis of BA_2_PbBr_4_ and DSC curve of Na_*x*_PbBr_*y*_ interacting with BABr (inset). **h** Qualitative comparison of nucleation energy and interfacial energy variation during HOIPs growth using Na_*x*_PbBr_*y*_ (pink line) vs PbBr_2_ (blue line). **i**–**l** Thickness-dependent growth of HOIP films. Insets: corresponding AFM images. Film thicknesses are 9 nm (**i**), 21 nm (**j**), 31 nm (**k**), and 55 nm (**l**), respectively. Scale bars, 50 µm (**a**), 100 µm (**b**–**d**), 25 µm (**e**), 20 µm (**f**), and 10 µm (**i**–**l**). Source data are provided as a Source Data file.

The 2D melt growth process of HOIP films with different thickness were recorded in situ using a CVD system equipped with optical microscopy (Supplementary Movies 1 and 2 and Methods). Figure 4a–e are extracted from Supplementary Movie 1 and present as in situ optical images that clearly illustrate the sequence of congruent melting (Fig. 4b), formation of a 2D liquid film (Fig. 4c), and subsequent crystallization into molecularly thin 2D HOIP film (Fig. 4d). In Fig. 4a, Na_*x*_PbBr_*y*_ forms a highly dense layer across the entire substrate, appearing as nanosized black dots on the SiO_2_/Si. In contrast, evaporating PbBr_2_ only forms microsized particles due to its poorer wetting property (Fig. 4f and Supplementary Fig. 14). To further verify the melting behavior of the Na_*x*_PbBr_*y*_ and BABr precursors, differential scanning calorimetry (DSC) measurements were performed. The DSC result reveals a melting temperature (*T*_m_) of 199.8 °C, which is well below the decomposition temperature (*T*_d_) of BA_2_PbBr_4_ (Fig. 4g and Supplementary Fig. 15). This melting temperature closely matches the substrate temperature of 210 °C used during growth within experimental error margin (Fig. 4c), thus confirming that the precursors are maintained in the liquid phase under this condition. Consequently, the reaction mechanisms of these two inorganic precursors with organic cation vapor (e.g., BA^+^) differ significantly: PbBr_2_ particles serve directly as heterogeneous nucleation sites for HOIP formation by vapor–solid reaction (Supplementary Movie 3 and Fig. 4f). In contrast, the nanosized and high-density Na_*x*_PbBr_*y*_ seeds melt readily upon reaction with organic precursor vapor, forming a liquid phase. Thereafter, homogeneous nucleation occurs in the liquid. The much larger energy barrier for homogeneous nucleation compared to heterogeneous nucleation gives a lower nucleation density in the liquid phase^23,38^, however, the rapid spreading of the liquid phase lowers the diffusion barrier (Fig. 4h), and 2D crystallization occurs fast once the initial seed of HOIP is formed. Importantly, controlled cooling rate plays a vital role in film formation. If the cooling rate is too rapid, dendritic crystals form (Supplementary Movie 4 and Supplementary Fig. 16a–c), a phenomenon influenced by the temperature gradient. Thickness-controlled growth of *n* = 1 (BA)_2_PbBr_4_ films has been achieved with thicknesses of 9 nm, 21 nm, 31 nm, and 55 nm, as shown in Fig. 4i–l, respectively. Similarly, thickness-controlled growth of *n* = 2 (BA)_2_(MA)Pb_2_Br_7_ films are shown in Supplementary Fig. 16d–i. The advantage of 2D HOIP growth by 2D melt growth lies in its low thermal budget, making it an efficient method for large-scale production.

### Large-scale device fabrication on grown ferroelectric HOIP film

**Fig. 5:**
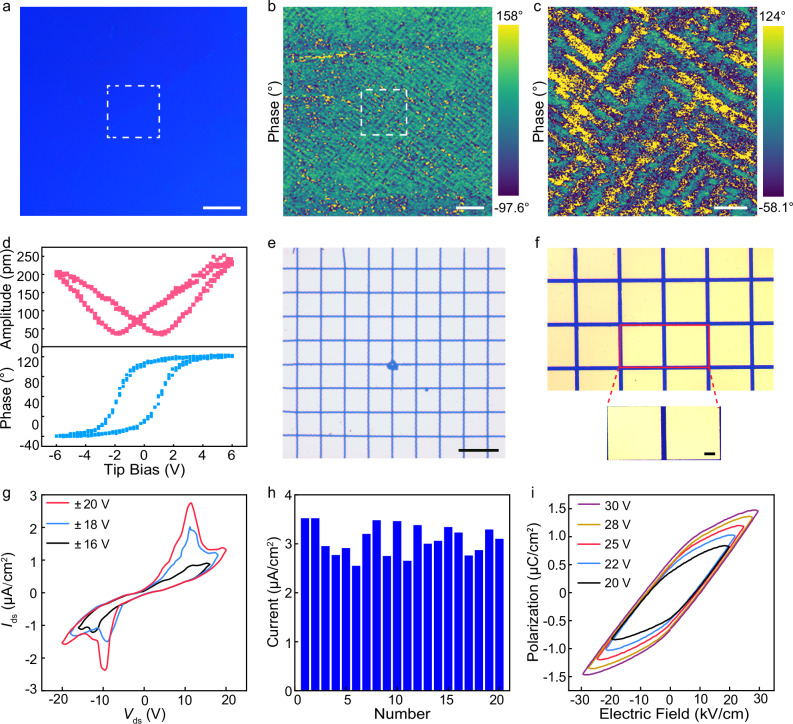
Electronic characterization of *n* = 2 ferroelectric HOIP film. **a** Optical image of 16-layer-thick single-crystalline *n* = 2 film. **b** Large-area, highly ordered ferroelectric domains observed by PFM in the region highlighted by the dashed line in (**a**). **c** Zoom-in PFM phase image scan of the region highlighted by the dashed line in (**b**). **d** Amplitude vs voltage and phase vs voltage curves for the *n* = 2 film. **e** Optical image of large-area two-terminal arrays directly fabricated on the grown *n* = 2 film on SiO_2_/Si. **f** Zoom-in optical image of source/drain pairs in (**e**), showing clear material morphology in the device channels. **g** Room-temperature *I*_ds_–*V*_ds_ curves for 16-layer *n* = 2 film device under different *V*_ds_ sweep ranges. **h** Statistical bar graph of the *I*_ds_ values measured from 20 devices. **i** *P–E* hysteresis loops taken at different voltage scan ranges. Scale bars, 10 µm (**a**), 2 µm (**b**), 0.5 µm (**c**), 200 µm (**e**), 20 µm (**f**). Source data are provided as a Source Data file.

The successfully grown single-crystalline *n* = 2 (BA)_2_(MA)Pb_2_Br_7_ film (Fig. 5a) exhibits robust in-plane ferroelectricity at room temperature. Due to weak van der Waals interlayer interaction in (BA)_2_(MA)Pb_2_Br_7_, long-range depolarization effects are prevented from propagating through the film, thus these films retain stable ferroelectricity even at thicknesses in the range of tens of nanometers^39^. A hallmark of single-crystalline films is their continuous, ordered lattice structure, enabling coherent alignment of electric dipoles across the film. This structural uniformity allows the spontaneous polarization of dipoles into well-defined domains with distinct orientations, thereby minimizing electrostatic energy^40–42^. Indeed, the optical image of a large-scale, 16-layer-thick *n* = 2 film (32 nm thick) in Fig. 5a reveals highly ordered orthogonally aligned ferroelectric domains with 180° phase contrast, as measured by PFM (Fig. 5b, c). Notably, such ferroelectric domain structures are absent in polycrystalline films due to their randomly oriented grains, preventing long-range dipole alignment. PFM measurement reveals a butterfly-shaped amplitude hysteresis loop and corresponding phase-voltage curve (Fig. 5d) confirm switchable ferroelectric polarization in the *n* = 2 film, complemented by a second harmonic generation response (Supplementary Fig. 17a).

Electrode arrays were directly fabricated on the grown film on SiO_2_/Si to evaluate its ferroelectric performance. Figure 5e shows large-scale evaporated electrodes, with a zoomed-in source/drain pair illustrated in Fig. 5f. The *I*_ds_–*V*_ds_ curves under varying bias voltages exhibit hysteresis loops that increases with increasing *V*_ds_, a characteristic of ferroelectric switching (Fig. 5g). The statistical *I*_ds_ data from 20 devices, demonstrating the uniformity of the film in terms of the *I*_ds_ values, with an average value of 3.08 µA/cm^2^ (Fig. 5h and Supplementary Fig. 17b). Notably, *I*_ds_ increases with film thickness, reaching 11.8 µA/cm^2^ for a thickness of 75 nm (Supplementary Fig. 17c, d). Importantly, the *E*_c_ of our film (10 kV/cm) (Fig. 5i) is much lower than most reported *E*_c_ values for solution-process HOIP films of several tens of kV/cm (Supplementary Table 2), which can be attributed to the lower defect density in the single-crystalline films. In contrast, polycrystalline films contain grain boundaries that pin domain wall motion, resulting in a higher *E*_c_.

We demonstrate a substrate-agnostic strategy for synthesizing single-crystalline 2D and quasi-2D HOIP films through a 2D melt growth technique, enabling proof-of-concept fabrication of ferroelectric devices at scale. Unlike conventional 2D Czochralski methods for TMDs, which rely on inorganic eutectic phase formation with molten glass, our approach exploits the low-temperature reaction between HOIP precursors’ organic cation vapor and an inorganic seeding layer to generate a 2D molten phase. This process occurs at HOIP’s inherently low melting temperature and is fully compatible with vacuum-based thermal processing, offering a scalable pathway for batch fabrication of HOIP films on CMOS-compatible architecture. The versatility of this method is anticipated to advance the synthesis of diverse single-crystal HOIP systems. In addition to HOIPs based on aliphatic chains and cyclic organic cations, HOIPs incorporating aromatic cations can also be grown using our 2D melt growth approach. Moreover, congruent melting has been reported for chiral NEA-based HOIPs, halogen-substituted PEA-based HOIPs, and even 3D perovskites^43–45^. These findings highlight strong potential for extending the 2D melt growth strategy to these systems. Compared to furnace-type CVD, organic molecular beam epitaxy provides a more tunable growth method, affording wafer-scale growth with precise thickness control. Bypassing substrate limitations and enabling parallel production, this work provides a foundation for expanding HOIP-based devices into large-scale industrial applications, including integrated HOIP/Si photovoltaics, photonics, and neuromorphic computing platforms.

## Methods

### Chemicals

n-Butylammonium bromide (BABr, >99%, powder), Methylammonium bromide (MABr, 99.99%, powder), BAI powder (>99%, powder) and n-Hexylammonium bromide (HABr, >99%, powder) were purchased from Greatcell Solar Materials Pty Ltd. Lead(II) bromide (PbBr_2_, 99.99%, powder) and lead(II) iodide (PbI_2_, 99.999%, powder) was purchased from Xi’an Yuri Solar Co., Ltd. Sodium hydroxide (NaOH, ACS reagent, ≥97%, pellets) was purchased from Sigma-Aldrich. All chemicals were used as received without further purification.

### 2D melt growth of centimetre-scale *n* = 1 (BA)_2_PbBr_4_ film

A horizontal furnace with dual temperature zones (Zone 1 and Zone 2) was utilized to grow HOIP films. The entire growth process was conducted under an inert atmosphere provided by high-purity N_2_ gas (99.9%). As illustrated in Supplementary Fig. 1a, the inorganic source-a mixture of PbBr_2_ (15 mg) and NaOH (5 mg) was placed in a crucible with dimensions of 10 cm × 1 cm × 1 cm (length × width × height) in Zone 1. The SiO_2_/Si substrate was positioned in Zone 2. Prior to the growth process, the SiO_2_/Si substrate was pre-soaked in a NaOH solution (2 mol/L) for 5 minutes. The distance between the inorganic source and the SiO_2_/Si substrate is denoted as “*D*” in Supplementary Fig. 1a. The growth process involves three consecutive steps: 1. Deposition of inorganic Na_*x*_PbBr_*y*_ precursor; 2. Molten phase formation and spreading of the 2D molten layer; 3. 2D crystallization of HOIPs. For the growth of *n* = 1 (BA)_2_PbBr_4_, the N_2_ flow rate was 80 sccm and the distance *D* was set to 19 cm. In step 1, the temperature of Zone 1 was ramped up to 580 °C over 30 minutes and maintained at this temperature for 20 minutes. During this phase, NaOH reacted sufficiently with PbBr_2_, resulting in the formation of Na_*x*_PbBr_*y*_. These phases vaporized and were carried by the N_2_ gas flow from Zone 1 to Zone 2. Simultaneously, the temperature of Zone 2 was set to 250 °C, enabling the vaporized Na_*x*_PbBr_*y*_ to deposit as a solid layer on the SiO_2_/Si substrate. Next, in step 2, the temperature of Zone 1 decreased to 420 °C, while Zone 2 decreased to 210 °C. At this stage, a crucible containing an excess amount of BABr powder (35 mg) was pushed into Zone 1 using a magnet. At 420 °C, BABr began to vaporize, whereas the inorganic source did not. The gaseous BABr was carried by the N_2_ gas flow to the SiO_2_/Si substrate (maintained at 210 °C). Upon contact with the Na_*x*_PbBr_*y*_ layer, a liquefaction process was triggered, resulting in a molten phase. The molten phase then spread uniformly across the SiO_2_/Si substrate to form a molten layer. In final step 3, a cooling rate of 1.0 °C/min was applied in Zone 2, gradually lowering the temperature from 210 °C to 190 °C. The heating and cooling scans of DSC measurement can be used to estimate the experimental melting temperature (210 °C) and crystallization temperature (190 °C) (Supplementary Fig. 15). During cooling from 210 °C to 190 °C, the crystallization has nearly completed. Subsequently, the cap of the CVD system was opened, and the sample was allowed to cool to room temperature under a continuous N_2_ gas flow.

### 2D melt growth of centimetre-scale *n* = 2 (BA)_2_(MA)Pb_2_Br_7_ film

For the growth of *n* = 2 (BA)_2_(MA)Pb_2_Br_7_, the entire process was conducted under N_2_ gas (99.9%) with a flow rate of 140 sccm. The distance *D* was set to 19 cm. The organic source was modified to a mixture of BABr (30 mg) and MABr (10 mg). Step 1 is identical to the procedure used for the growth of *n* = 1 (BA)_2_PbBr_4_. In step 2, the temperature of Zone 2 was reduced to 200 °C. In step 3, the cooling rate was set to 1.3 °C/min, cooling the temperature from 200 °C to 188 °C.

### 2D melt growth of centimetre-scale *n* = 3 (BA)_2_(MA)_2_Pb_3_Br_10_ film

For the growth of *n* = 3 (BA)_2_(MA)_2_Pb_3_Br_10_, the entire process was conducted under N_2_ gas (99.9%) with a flow rate of 80 sccm. The distance *D* was set to 18 cm. The organic source was modified to a mixture of BABr (31 mg) and MABr (22 mg). Step 1 is identical to the procedure used for the growth of *n* = 1 (BA)_2_PbBr_4_. In step 2, the temperature of Zone 2 was reduced to 175 °C. In step 3, the temperature of Zone 2 was allowed to cool naturally from 175 °C to 150 °C, after which the cap of the CVD system was opened.

### 2D melt growth of centimetre-scale *n* = 1 (HA)_2_PbBr_4_ film

For the growth of *n* = 1 (HA)_2_PbBr_4_, the entire process was conducted under N_2_ gas (99.9%) with a flow rate of 80 sccm. The distance *D* was set to 17 cm. The organic sources used were HABr (32 mg), and a mixture of PbBr_2_ (21 mg) and NaOH (7 mg) was used as the inorganic source. Step 1 is identical to the procedure used for the growth of *n* = 1 (BA)_2_PbBr_4_. In step 2, the temperature of Zone 2 was reduced to 230 °C. In step 3, the cooling rate was set to 2 °C/min, cooling the temperature of Zone 2 from 230 °C to 196 °C.

### 2D melt growth of centimetre-scale *n* = 2 (HA)_2_(MA)Pb_2_Br_7_ film

For the growth of *n* = 2 (HA)_2_(MA)Pb_2_Br_7_, the entire process was conducted under N_2_ gas (99.9%) with a flow rate of 80 sccm. The distance *D* was set to 17 cm. The organic source was modified to a mixture of HABr (36 mg) and MABr (11 mg). Step 1 is identical to the procedure used for the growth of (BA)_2_PbBr_4_ (*n* = 1). In step 2, the temperature of Zone 2 was reduced to 220 °C. In step 3, the cooling rate was set to 2.2 °C/min, cooling the temperature of Zone 2 from 220 °C to 200 °C.

### 2D melt growth of centimetre-scale *n* = 1 (BA)_2_PbI_4_ film

For the growth of *n* = 1 (BA)_2_PbI_4_, the entire process was conducted under N_2_ gas (99.9%) with a flow rate of 80 sccm. The distance *D* was set to 19 cm. The organic source used was BAI (35 mg), and a mixture of PbI_2_ (30 mg) and NaOH (8 mg) was used as the inorganic source. In step 1, the temperature of Zone 1 was ramped to 520 °C over 25 minutes and maintained at this temperature for 20 minutes. Simultaneously, Zone 2 was heated to 250 °C. In step 2, the temperatures of Zone 1 and Zone 2 were reduced to 370 °C and 213 °C, respectively. In step 3, Zone 2 was cooled from 213 °C to 200 °C at a rate of 1 °C/min, after which the cap of the CVD was opened.

### Thickness-dependent growth of *n* = 1 (BA)_2_PbBr_4_ film

To achieve thickness-dependent film growth, the amount of inorganic Na_*x*_PbBr_*y*_ deposited on the SiO_2_/Si substrate is the determining factor, while the organic precursor was supplied in excess. Any excess organic vapor that did not interact with the inorganic phases was vaporized and removed by the N₂ gas as exhaust. Both the raw amount of inorganic precursor (the mass of NaOH and PbBr_2_) and the distance *D* were adjusted to precisely control the film thickness. For the growth of *n* = 1 BA_2_PbBr_4_ films with thicknesses of 9 nm, 21 nm, 31 nm, and 55 nm, the required mass of inorganic precursors and corresponding distances *D* were: PbBr_2_ (15 mg) and NaOH (5 mg) with *D* = 19 cm, PbBr_2_ (18 mg) and NaOH (6 mg) with *D* = 18 cm, PbBr_2_ (24 mg) and NaOH (8 mg) with *D* = 17 cm, PbBr_2_ (30 mg) and NaOH (10 mg) with *D* = 16 cm, respectively. The organic precursor BABr was maintained at an excess amount of 35 mg in all the cases above.

### Thickness-dependent growth of *n* = 2 (BA)_2_(MA)Pb_2_Br_7_ film

For the growth of *n* = 2 (BA)_2_(MA)Pb_2_Br_7_ films with thicknesses of 15 nm, 29 nm and 51 nm, the required mass of inorganic precursors and corresponding distances *D* were: PbBr_2_ (15 mg) and NaOH (5 mg) with *D* = 20 cm, PbBr_2_ (18 mg) and NaOH (6 mg) with *D* = 18.5 cm, PbBr_2_ (24 mg) and NaOH (8 mg) with *D* = 17 cm, respectively. The organic precursor was a mixture of BABr (30 mg) and MABr (10 mg) in the cases above.

### In situ imaging of 2D melt growth of (BA)_2_PbBr_4_ film

The growth process was carried out in a dual-zone tubular furnace equipped with optical microscopy and a water-cooling system (Micro-STS1200, Units Technology). Na_*x*_PbBr_*y*_ seeding layer on SiO_2_/Si was placed in Zone 2. N_2_ gas with 20 sccm is fed into the furnace. When the temperature of Zone 1 reached 420 °C, a small amount of BABr powder was introduced into Zone 1. The subsequent growth procedure followed Step 2 and Step 3 described for the growth of *n* = 1 (BA)_2_PbBr_4_ film above.

### Synchrotron-based GIXRD measurement

Synchrotron-based GIXRD experiments were performed at the PLS-II 6D UNIST-PAL beamline of the Pohang Accelerator Laboratory, Korea. The GIXRD measurements were performed on samples with diameters of approximately 2-3 mm. Using *ω*-scan GIXRD, which involves capturing diffraction patterns while rotating the sample around its normal axis, the analysis covered areas ranging from sub-cm^2^ to cm^2^ regions of the sample. The X-rays from the bending magnet were monochromatized to 18.986 keV (*λ* = 0.6530 Å) using a double-crystal monochromator and focused both horizontally and vertically (90 (V) × 120 (H) μm^2^ full width at half maximum) at the sample position via a sagittal Si (111) crystal and a toroidal mirror, respectively. The GIXRD sample chamber was equipped with a 5-axis motorised stage for fine sample alignment. The NIST SRM660b (LaB6) was used to calibrate the diffraction angle. GIXRD patterns were recorded using a 2D-CCD detector (MX 225-HS, Rayonix L.L.C., USA) with a sample-to-detector distance of 240.08 mm. An incidence angle of 0.117° was used, and each GIXRD pattern was obtained with 1 second X-ray exposure while rotating every 1° in the in-plane direction.

### TEM specimen preparations and measurements

HOIP films were directly grown on an ultrathin carbon film on a Mo TEM grid via CVD for in-plane characterization. Cross-sectional specimens for cryogenic TEM analysis were prepared from HOIP films grown on SiO_2_/Si substrates using an Aquilos cryogenic dual-beam SEM-FIB system. Before FIB preparation, a protective gold (Au) layer (~50 nm) was deposited on the surface of the HOIPs film. The sample was subsequently loaded into the FIB chamber under liquid nitrogen conditions, with the sample stage maintained at ~78 K throughout the process. An additional layer of organometallic platinum (Pt) precursor (~1 μm) was deposited in situ at ~78 K to form a Pt–C protective layer over the Au layer. Using standard FIB procedures combined with a focused Ga⁺ ion beam at low current, a thin cross-section specimen was prepared. The resulting FIB lamellae were then transferred under liquid nitrogen to a cryo-TEM for further imaging. Na_*x*_PbBr_*y*_ specimens were directly grown onto ultrathin carbon film-coated TEM grids via CVD for characterization.

ADF-STEM imaging was performed using an aberration-corrected JEOL ARM200F TEM operating at 200 kV, equipped with a super dual EDS system. Low-dose STEM imaging was conducted with an 11 pA beam current, a convergence angle of 29.2 mrad, and a collection angle of 54-220 mrad. HRTEM imaging was carried out using an aberration-corrected Titan Krios TEM operating at 300 kV and a Talos F200X G2 TEM operating at 200 kV. Low-dose cryogenic TEM imaging was performed at ~78 K using a K3 direct-detection electron-counting camera controlled by the Serial EM software package. To mitigate beam-induced degradation of the organic components, a dose-fractionation strategy commonly used in structural biology was employed. Multi-frame TEM images were recorded at high frame rates over 1–2 s, followed by computational alignment and summation after precise drift correction (17 frames per second, 0.88 e^−^/Å^2^ per frame). 3D electron diffraction data were collected using the EPUD acquisition software. The specimen stage was rotated serially from ±55°, with data collected every 0.5°. The diffraction data were processed using the REDp and SHELXL software packages.

HRTEM simulations were performed using the ToTEM software with a multislice algorithm. The simulation parameters were set to match the experimental conditions. For the in-plane models (Supplementary Figs. 1c and 2b), both *n* = 1 and *n* = 2 structures were simulated with a model thickness of ~10 nm, a pixel size of 0.2 Å/pixel, and defocus values of 20 nm and −155 nm, respectively. Cross-sectional models for *n* = 1 and *n* = 2 were simulated with a thickness of ~20 nm, a pixel size of 0.68 Å/pixel, and defocus values of −450 nm and −650 nm, respectively (Fig. 1g, h). The accelerating voltage was 300 kV, and spherical aberration coefficients were set according to measured values from the image corrector (A1 = 1 nm; A2, B2 = 20 nm; C3 = 7 μm; A3, S3 = 1 μm; A4, D4, B4 = 40 μm; C5, A5 = 10 mm). Electron diffraction patterns and reciprocal lattices were simulated using CrystalMaker with a model thickness of 10 nm.

### Device fabrications and electrical measurements

For the fabrication of the device array, a Cu hard mask with channel widths of 115 µm and channel lengths of 10 µm was transferred onto the surface of the ferroelectric (BA)_2_(MA)Pb_2_Br_7_ film on SiO_2_/Si using a transfer stage in a glove box. Subsequently, Ti/Au metal electrodes were deposited via thermal evaporation. Finally, the hard mask was removed to complete the fabrication process. *I*–*V* curves were measured using a probe station equipped with a Keithley 4200 semiconductor tester under a base pressure of 10^−^^2^ Pa. The *P*–*E* loops were recorded using the TF ANALYZER 3000 system from aixACCT System GmbH (Germany).

### Calculation of formation enthalpy

First-principles calculations were carried out by the plane wave code Vienna ab initio simulation package (VASP)^46^ with the projector augmented wave (PAW) method. Exchange-correlation energy was performed under the generalized gradient approximation (GGA) with the form of Perdew-Burke-Ernzerhof (PBE)^47^. DFT-D3 functional with Grimme correction was employed to describe the van der Waals interaction^48^. An energy cutoff of 400 eV and an energy convergence of 10^−4 ^eV were used in all calculations. All the structures were optimized until the forces exerted on each atom are <0.005 eV Å^−1^. For the formation enthalpies calculation, the BABr were simulated in an implicit solvent model through the VASPsol package^49^. The formation enthalpies were calculated at 515 K from the bond energies and vibrational frequencies by using a normal mode analysis through the TAMkin package^50^.

### Molecular dynamics simulations

For the free energy calculation well-tempered metadynamics (WTmetaD) simulation was implemented through CP2K combined with the PLUMED plugin^51,52^. All the WTmetaD simulations were performed at 515 K and 1 atm with NPT ensembles and a timestep of 1.0 fs. The Nosé-Hoover thermostat and Martyna–Tobias–Klein barostat were used with coupling constants of 1.0 ps and 1.0 ps, respectively.

In the WTmetaD simulations, a time increment of 20 fs was adopted, a Gaussian height was set to be 5.0 kJ/mol, the sigma is 0.25 Å, and the bias factor is 100. All the simulations were run for a minimum of 65.0 ps to ensure convergence and obtain reliable results. For tracking the formation of Pb^2+^ octahedron, the collective variable (CV) was introduced and defined as the coordination number (CN)^53^ of the Br with Pb:

5 $${CN}=\frac{1-{(\frac{r}{{r}_{0}})}^{n}}{1-{(\frac{r}{{r}_{0}})}^{m}}$$

where *r* is the distance between the ion and the Pb atoms, and *r*_0_ is set to be 3.0 Å. The *n* and *m* parameters of the switching function are set to 6 and 12, respectively.

### Characterizations

The optical images were acquired using a Leica DM2700 M microscope controlled by Leica LAS X software and a Nikon ECLIPSE LV150N microscope controlled by NIS-Elements software. No alterations or filters were applied to the optical images. The single crystal XRD diffraction measurements were conducted using a Rigaku SmartLab X-ray diffractometer with CuKα radiation. The photoluminescence spectrum and mapping of (BA)_2_(MA)Pb_2_Br_7_ were measured using a pulsed diode 405 nm wavelength laser in a vacuum chamber (PDL 800-D). For the photoluminescence spectrum of (BA)_2_PbBr_4_ and (HA)_2_PbBr_4_, a 365 nm wavelength was used for excitation (Edinburgh FLS980, xenon lamp excitation). Raman spectra and mapping were measured at 80 K using a confocal Raman microscopy system (WITec alpha300R) with a 532 nm continuous-wavelength excitation laser. SHG spectra were performed under vacuum conditions using a 1064 nm wavelength excitation laser (Rainbow 1064 OEM). PFM measurements were conducted in air using a scanning probe microscope (Asylum MFP-3D Infinity). AFM measurements were conducted using a Bruker Dimension ICON system placed in the glove box and a scanning probe microscope (Asylum MFP-3D Infinity) in air. SEM images were captured using a field-emission scanning electron microscope (FE-SEM, Tescan MAIA3). XPS measurements were performed using a monochromatic Al Kα X-ray source (12 kV, 400 μm spot size; Thermo Fisher Scientific Nexsa). Contact angle measurements were conducted using an OCA25-HTV1800 system (Dataphysics). TGA was measured under a N_2_ flow of 50 sccm (Mettler Toledo TGA/DSC3+). DSC were measured under a N_2_ flow of 50 sccm (Mettler Toledo DSC3).

### Reporting summary

Further information on research design is available in the Nature Portfolio Reporting Summary linked to this article.

## Supplementary information

Supplementary Information

Description of Additional Supplementary Files

Supplementary Movie 1

Supplementary Movie 2

Supplementary Movie 3

Supplementary Movie 4

Supplementary Data 1

Supplementary Data 2

Supplementary Data 3

Supplementary Data 4

Reporting Summary

Transparent Peer Review file

## Source data

Source Data 1

Source Data 2

## Data Availability

The authors declare that all the data supporting the findings of this study are available within the article (and Supplementary Information Files), or available from the corresponding author on request. Source data are provided with this paper.
