# Supplementary material for: Two-dimensional melt growth of large-scale, single-crystalline hybrid organic-inorganic perovskite films
Source: Nat Commun. 2026 Jun 3;17:7169. doi: 10.1038/s41467-026-73886-4 (PMC13396412; doi:10.1038/s41467-026-73886-4)
Supplement: Supplementary file 1 — Supplementary Information [file 41467_2026_73886_MOESM1_ESM.pdf]

Supplementary Information

Two-dimensional melt growth of large-scale, single-crystalline hybrid organic-inorganic perovskite films

Yuanyuan Jin[1,#], Gang Wang[2,#], Qiye Guan[3], Yixin Li[1], Tae Joo Shin[4], Seulyi Lee[5], Tingting Li[2], Song Liu[6], Guankui Long[7], Philip C.Y. Chow[8], Yongqing Cai[3], Kian Ping Loh[9], Junhao Lin[2,10]*, Kai Leng[1]*

[1]Department of Applied Physics, The Hong Kong Polytechnic University, Hong Kong, China.

[2]State Key Laboratory of Quantum Functional Materials, Department of Physics, Guangdong Basic Research Center of Excellence for Quantum Science, Southern University of Science and Technology (SUSTech), Shenzhen 518055, China.

[3]Institute of Applied Physics and Materials Engineering, University of Macau, Taipa, Macau, China.

[4]Graduate School of Semiconductor Materials and Devices Engineering, Ulsan National Institute of Science and Technology (UNIST), Ulsan 44919, Republic of Korea.

[5]Office of Research Facilities and Training, Ulsan National Institute of Science and Technology (UNIST), Ulsan 44919, Republic of Korea.

[6]College of Chemistry and Chemical Engineering, Hunan University, Changsha 410082, China.

[7]National Key Laboratory of Semiconductor Laser, School of Materials Science and Engineering, Nankai University, Tianjin, 300350, China.

[8]Department of Mechanical Engineering, The University of Hong Kong, Pokfulam 999077, Hong Kong, China.

[9]Department of Chemistry, National University of Singapore, Singapore 117543, Singapore.

[10]Quantum Science Center of Guangdong-Hong Kong-Macao Greater Bay Area (Guangdong), Shenzhen 518045, China.

[#]These authors contributed equally: Yuanyuan Jin, Gang Wang.

***Corresponding author. Email: linjh@sustech.edu.cn (J.L.); kathy-kai.leng@polyu.edu.hk (K.L.)**

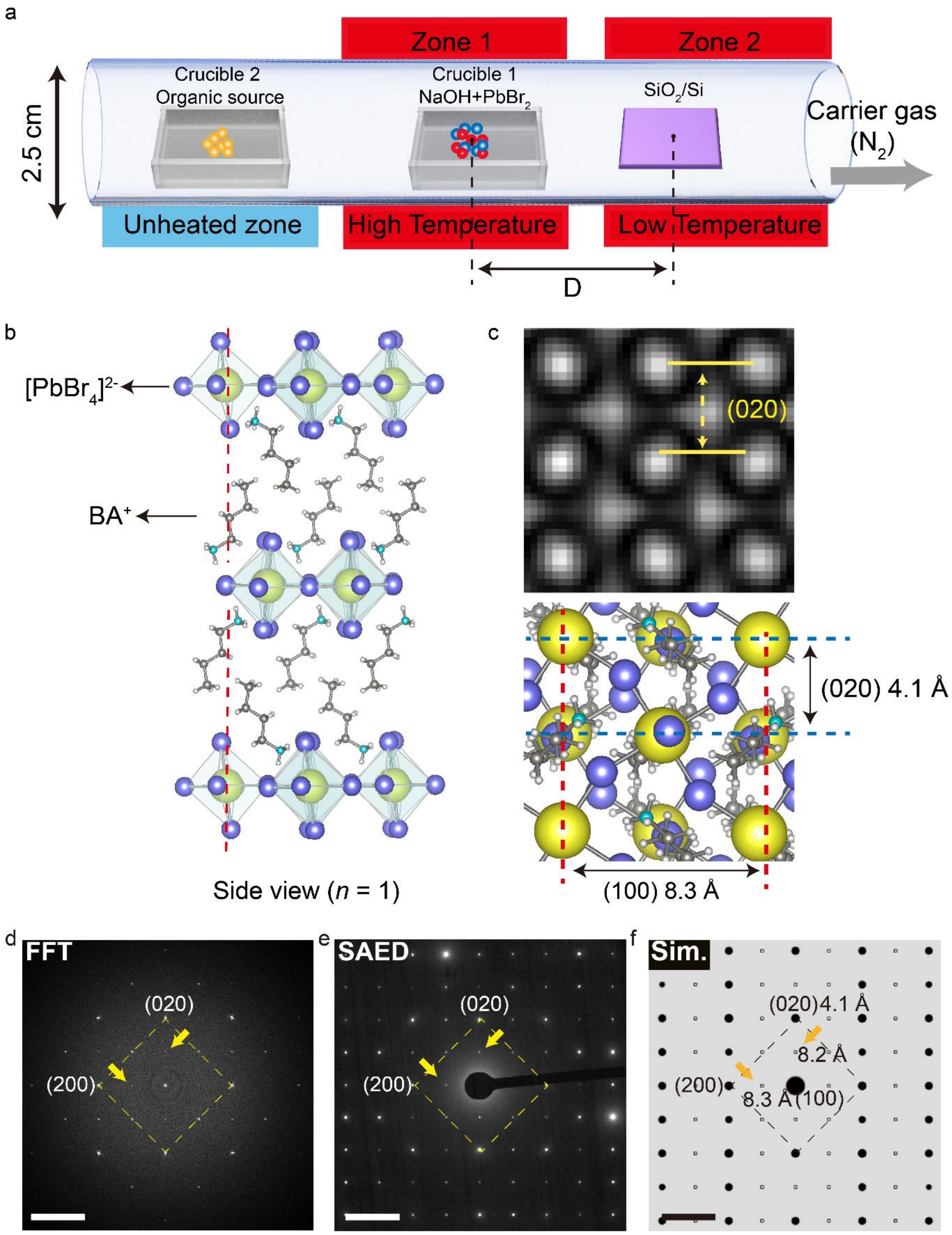

Supplementary Fig. 1 2D melt growth of single-crystal HOIPs and atomic structure of grown *n* = 1 $(BA)_2PbBr_4$ confirmed by TEM characterization. a, A schematic illustration of the 2D melt growth HOIP films via CVD, achieved by sequentially depositing inorganic and organic precursors in a horizontal furnace with dual temperature zones (Zone 1 and Zone 2). The distance between the inorganic source and the SiO_2/Si substrate is denoted as "*D*" (refer to the Methods section for detailed growth information). **b**, Side view atomic structural model of *n* =1 $(BA)_2PbBr_4$, showing the projection

columns of Pb atoms, apical Br atoms and portions of BA molecules (marked with a red dashed line), corresponding to the bright yellow dots in Fig. 1i in main text. **c**, HRTEM simulations (top) and single-unit-cell atomic models (bottom) viewed along the [001] direction of n = 1 $(BA)_2PbBr_4$ film. In the single-unit-cell atomic structure model, the (100) and (020) planes are highlighted with light red and blue lines, respectively, showing interplanar spacings of 8.3 Å and 4.1 Å. **d-f**, Fast Fourier transform (FFT) pattern (**d**), selected area electron diffraction (SAED) pattern (**e**), and simulated diffraction pattern (**f**) for the n = 1 grown film. The dashed boxes highlight the (020) reflections, which exhibit the highest diffraction intensity in both samples. Scale bars, 2 nm (**d-f**).

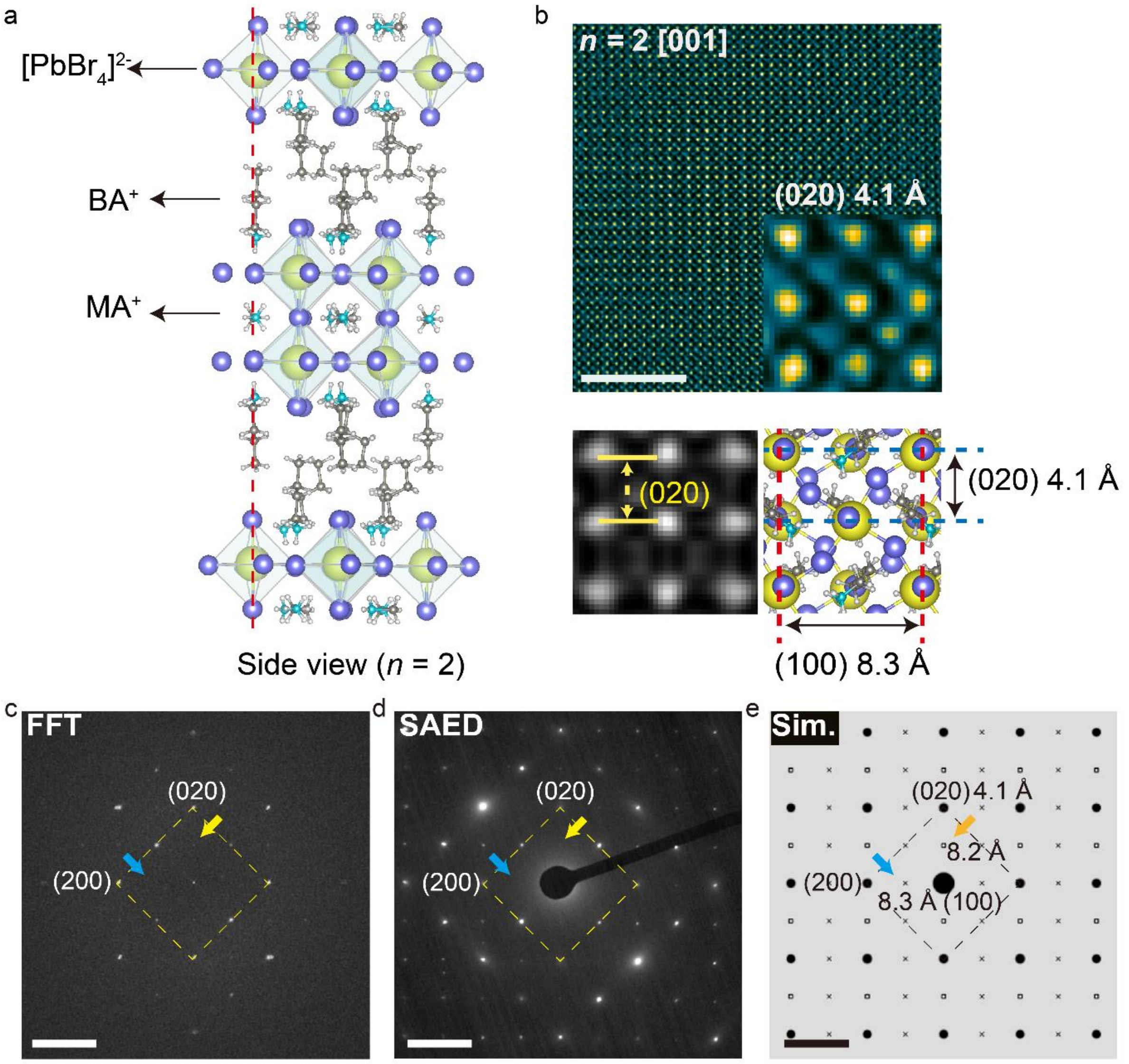

Supplementary Fig. 2 Atomic structure of grown *n* = 2 $(BA)_2(MA)Pb_2Br_7$ confirmed by TEM characterization. a, Side view atomic structural model of *n* = 2, displaying the projection columns of Pb atoms, apical Br atoms, portions of BA molecules, and MA ions (marked with a red dashed line), corresponding to the yellow dots in (**b**). **b**, Cryogenic HRTEM images of *n* = 2 $(BA)_2(MA)Pb_2Br_7$ film viewed along the [001] direction. Inset: magnified single-unit-cell HRTEM images for *n* = 2 $(BA)_2(MA)Pb_2Br_7$. Image is accompanied by corresponding HRTEM simulations and atomic models displayed in the bottom-left and bottom-right corners, respectively. The (100) and (020) planes in structural model are highlighted with light red and blue lines, respectively, showing interplanar spacings of 8.3 Å and 4.1 Å. **c-e**, Corresponding FFT (**c**), SAED (**d**), and simulated diffraction pattern (**e**) for the *n* = 2 grown film. The primary difference between the reciprocal lattices of *n* = 1 and *n* = 2 lies in the (100) reflection series, which show distinct extinction behaviors. Diffraction spots indicated by yellow arrows appear with weak intensity, while those marked with blue arrows are absent in *n* = 2. Scale bars, 5 nm (**b**), 2 nm (**c-e**). Source data are provided as a Source Data file.

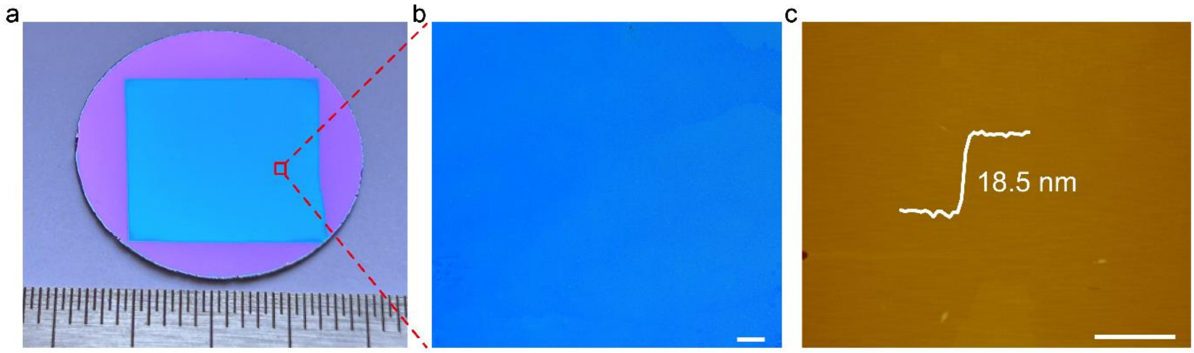

Supplementary Fig. 3 Centimetre-scale $(HA)_2PbBr_4$ single-crystalline film grown on SiO_2/Si by 2D melt growth. **a**, Photograph of n = 1 $(HA)_2PbBr_4$ single-crystalline film grown on SiO_2/Si wafer. **b**, Zoom-in optical image of n = 1 $(HA)_2PbBr_4$ film, corresponding to the red box marked area in (**a**). **c**, Corresponding AFM image showing a thickness of 18.5 nm for (**a**). Scale bars, 50 μm (**b**), 8 μm (**c**). Source data are provided as a Source Data file.

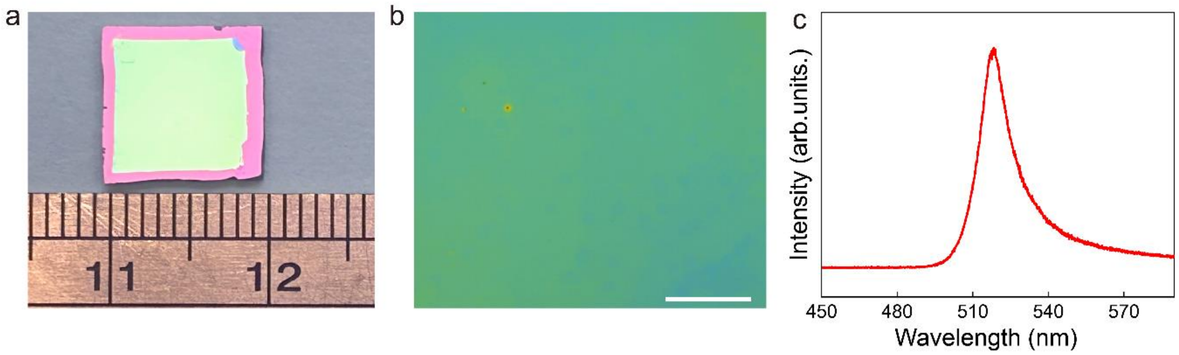

Supplementary Fig. 4 Centimetre-scale $(BA)_2PbI_4$ single-crystalline film grown on SiO_2/Si by 2D melt growth. **a**, Photograph of $n = 1$ $(BA)_2PbI_4$ film grown on SiO_2/Si. **b**, Zoom-in optical image of $n = 1$ $(BA)_2PbI_4$ film. **c**, Photoluminescence (PL) emission spectrum of $n = 1$ $(BA)_2PbI_4$ grown film. Scale bar, 50 μm (**b**). Source data are provided as a Source Data file.

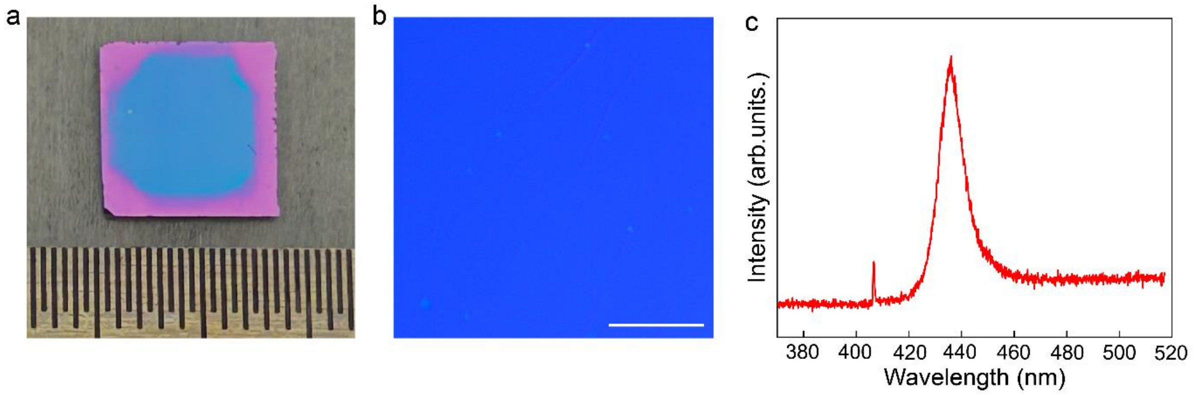

Supplementary Fig. 5 Centimetre-scale $(HA)_2(MA)Pb_2Br_7$ film grown on SiO_2/Si by 2D melt growth. a, Photograph of $n = 2$ $(HA)_2(MA)Pb_2Br_7$ film grown on SiO_2/Si. **b**, Zoom-in optical image of $n = 2$ $(HA)_2(MA)Pb_2Br_7$. **c,** PL emission spectrum of $n = 2$ $(HA)_2(MA)Pb_2Br_7$ grown film. Scale bar, 25 μm (**b**). Source data are provided as a Source Data file.

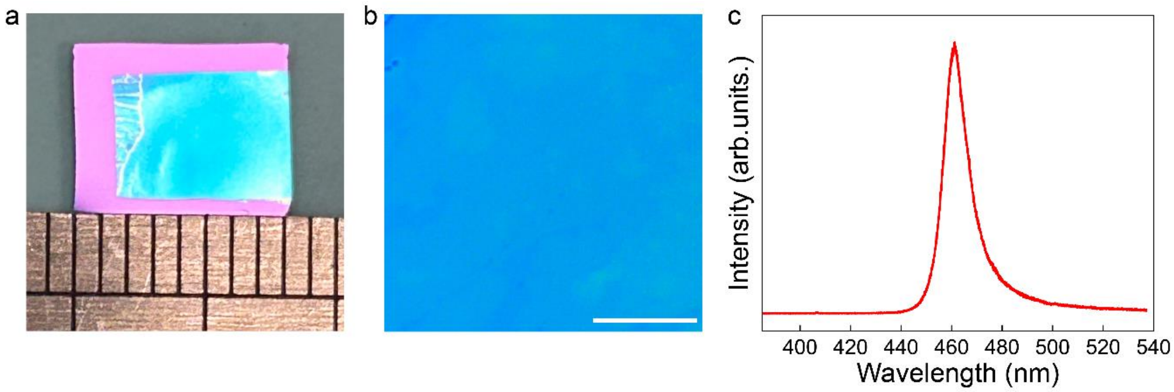

Supplementary Fig. 6 Centimetre-scale $(BA)_2(MA)_2Pb_3Br_{10}$ film grown on SiO_2/Si by 2D melt growth. **a**, Photograph of *n* = 3 $(BA)_2(MA)_2Pb_3Br_{10}$ film grown on SiO_2/Si. **b**, Zoom-in optical image of *n* = 3 $(BA)_2(MA)_2Pb_3Br_{10}$. **c,** PL emission spectrum of *n* = 3 $(BA)_2(MA)_2Pb_3Br_{10}$ grown film. Scale bar, 50 μm (**b**). Source data are provided as a Source Data file.

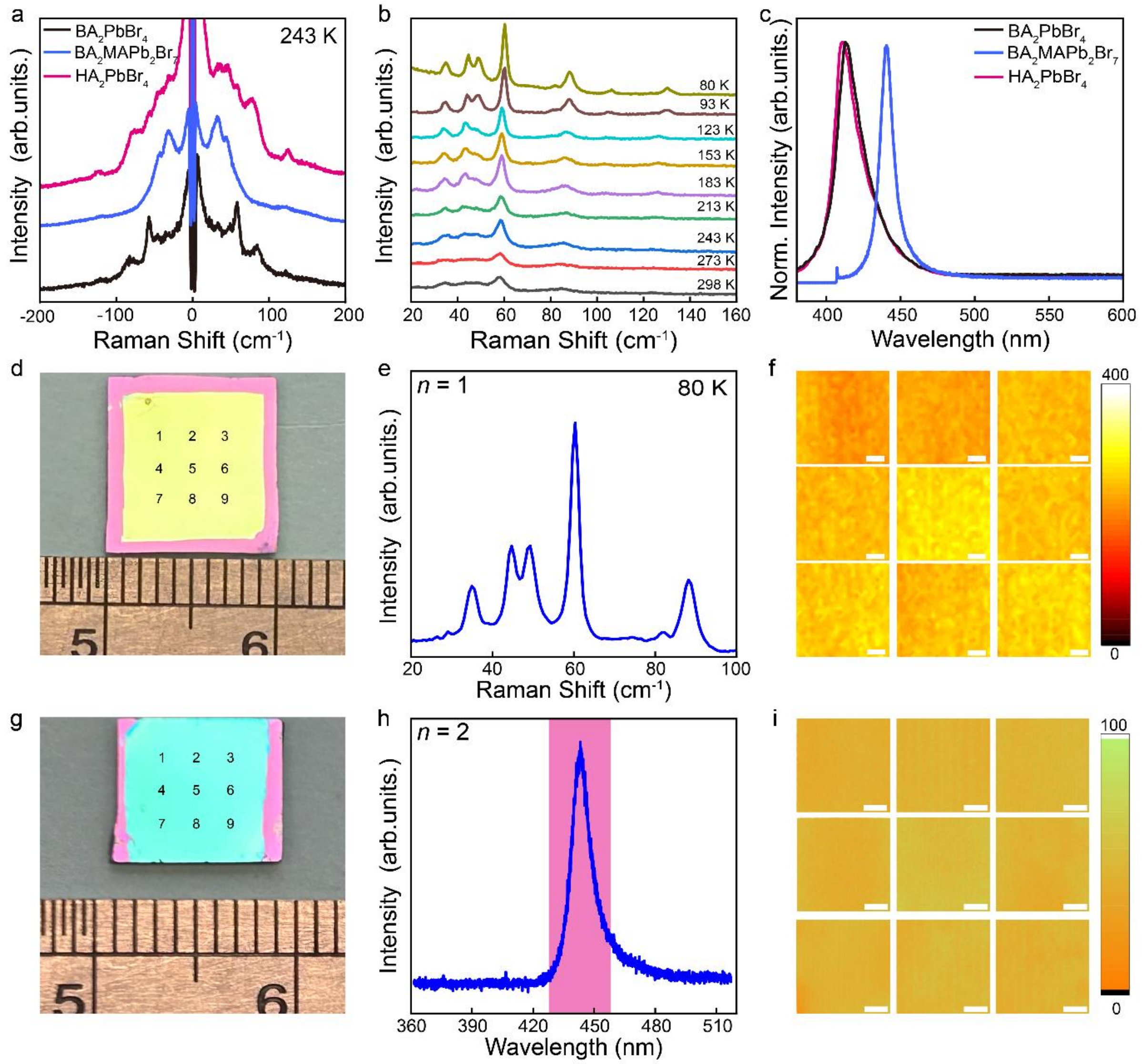

Supplementary Fig. 7 Characterization of uniformity of grown HOIP films. **a**, Raman spectra of the three types of grown HOIP films. The positions of their characteristic peaks are consistent with reported values[1,2]. **b**, Temperature-dependent Raman spectra of n = 1 $(BA)_2PbBr_4$ film. **c**, PL spectra of n = 1 $(BA)_2PbBr_4$ film (black line), n = 2 $(BA)_2(MA)Pb_2Br_7$ film (blue line) and n = 1 $(HA)_2PbBr_4$ film (pink line). **d**, Photograph of large-scale n = 1 $(BA)_2PbBr_4$ film grown on SiO_2/Si divided into nine regions. **e**, Raman spectrum of (**d**) measured at T = 80 K. **f**, Raman intensity mapping of the nine regions 1-9 labeled in (**d**). **g**, Photograph of large-scale n = 2 $(BA)_2(MA)Pb_2Br_7$ film grown on SiO_2/Si. **h**, PL spectrum of (**g**) measured at room temperature. **i**, PL intensity mapping of the nine regions 1-9 labeled in (**g**). Scale bars, 1 μm (**f**), 5 μm (**i**). Source data are provided as a Source Data file.

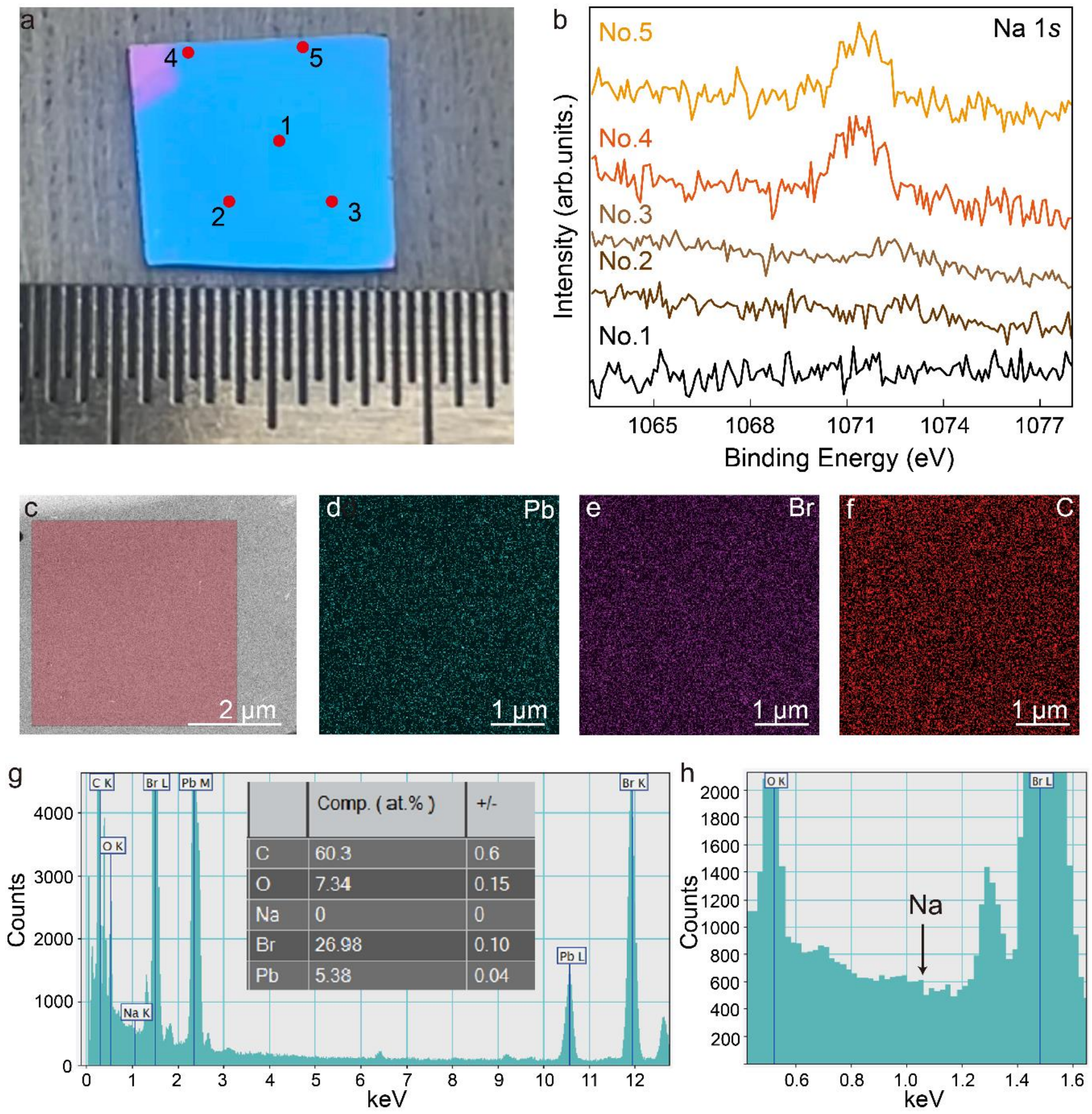

Supplementary Fig. 8 Characterization of NaBr by-product. a, Photograph of $n = 1$ BA_2PbBr_4 film on SiO_2/Si. **b**, Corresponding Na 1*s* XPS spectra collected at five positions marked in (**a**). **c**, TEM image of BA_2PbBr_4 film grown on a TEM grid. **d-f**, Elemental maps of Pb (**d**), Br (**e**) and C (**f**) acquired from the red rectangular region in (**c**). **g**, EDS spectrum acquired from the red rectangular region in (**c**). **h**, Zoom-in EDS spectrum on the Na-related energy range. Source data are provided as a Source Data file.

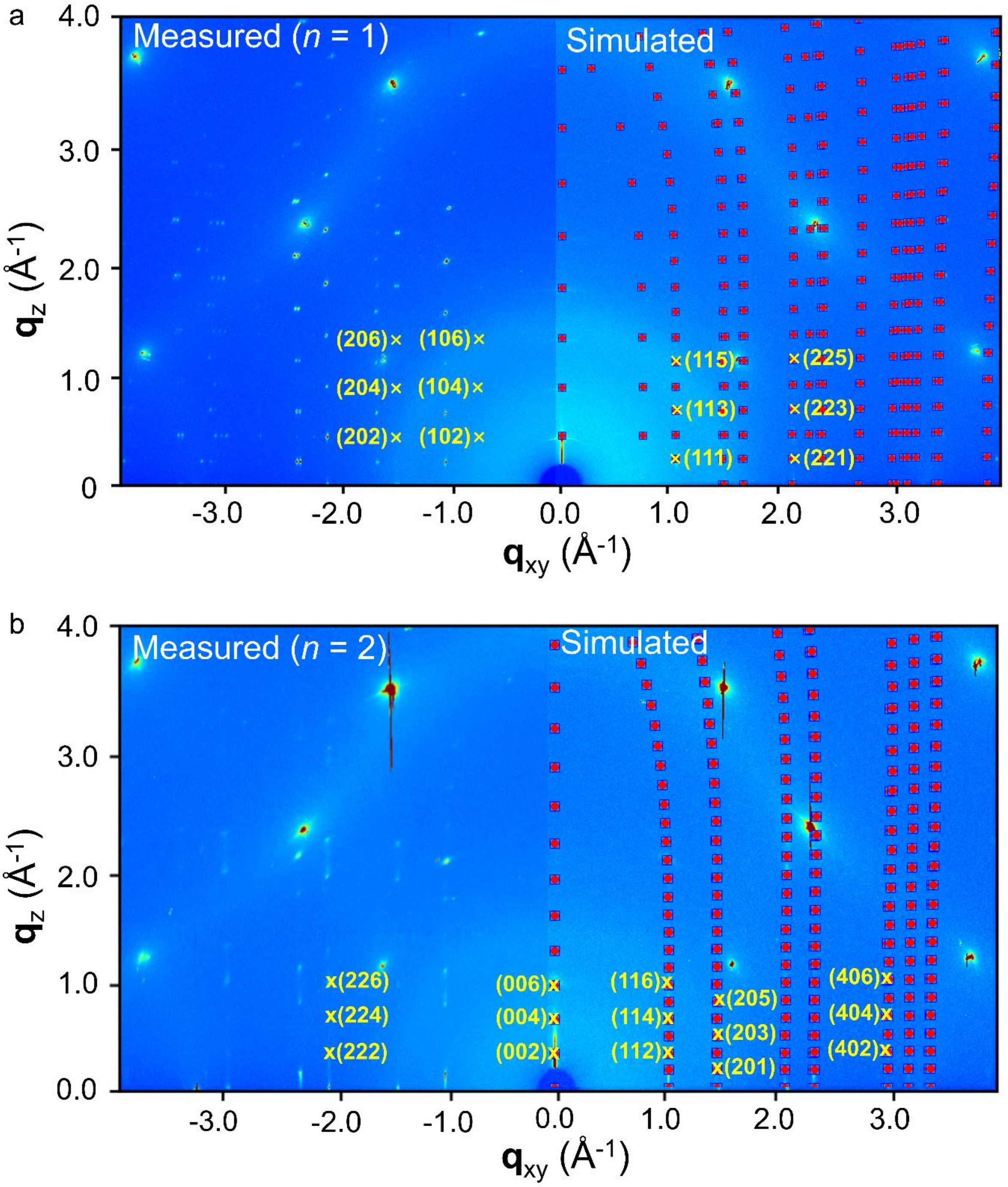

Supplementary Fig. 9 Verification of the large-area single-crystallinity of grown HOIP films. a, Structural analysis of the summed ω-dependent (0°-180°) GIXRD patterns of the n = 1 film. **b**, Structural analysis of the summed ω-dependent (0°-360°) GIXRD patterns of the n = 2 film. The left panel presents the measured GIXRD patterns, while the right panel displays the simulated patterns derived from single-crystal diffraction data. Source data are provided as a Source Data file.

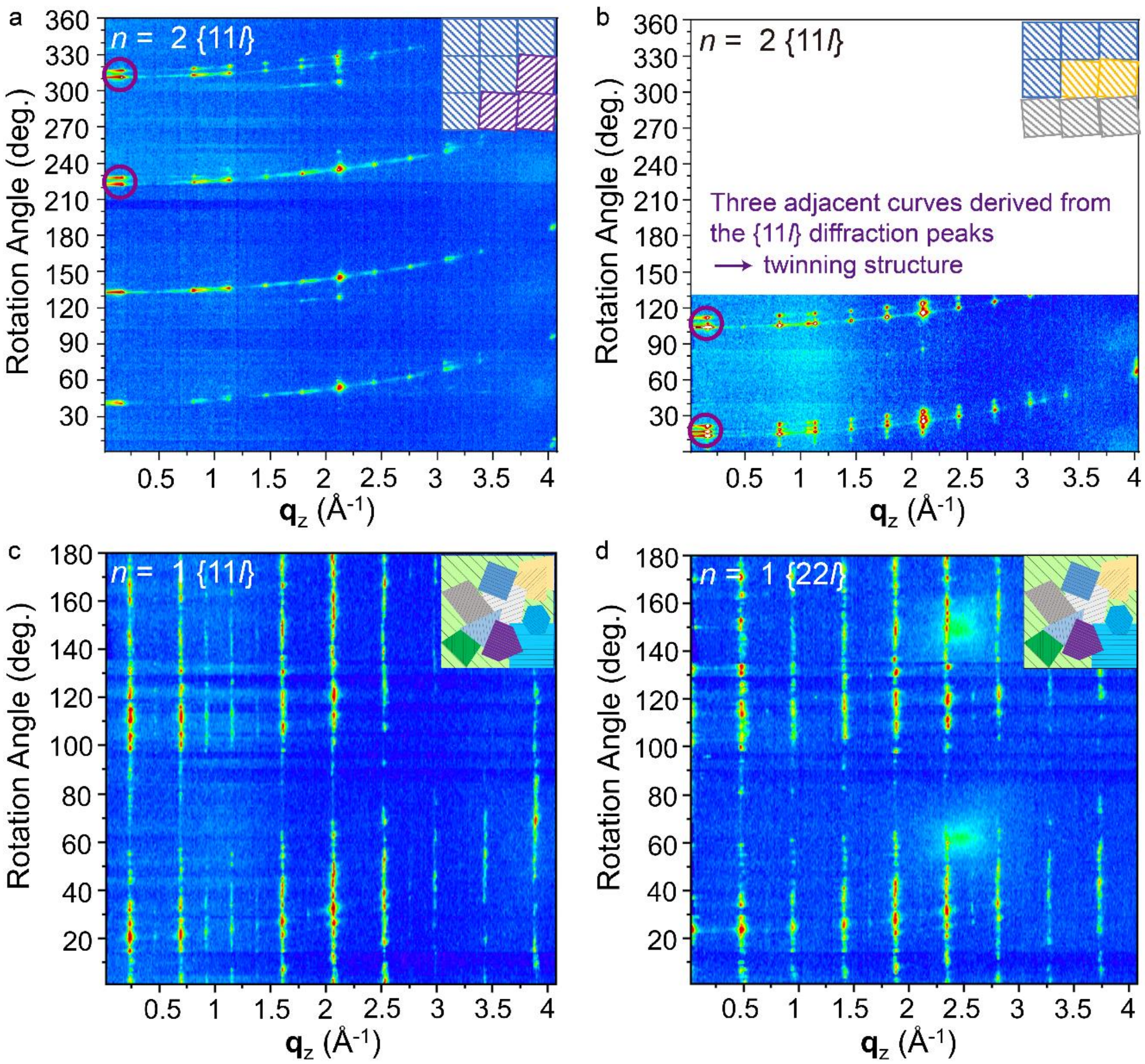

Supplementary Fig. 10 GIXRD results of twinning and polycrystalline structures. a,**b,** Typical $\mathbf{q}_z$ vs. ω results for the n = 2 $(BA)_2(MA)Pb_2Br_7$ film, revealing a twinning structure with two (**a**) and three (**b**) adjacent splitting curves derived from the {11l} diffraction spots. The number of adjacent curves is easily distinguished from the area circled with purple lines. **c,d,** The $\mathbf{q}_z$ vs. ω results for the n = 1 $(BA)_2PbBr_4$ polycrystalline film derived from {11l} (**c**) and {22l} (**d**) diffraction spots. The vertical lines indicate diffraction spots at all rotation angles, attributed to the random orientation of the polycrystals. Inset: schematic illustration of the corresponding twin and polycrystalline structures. Source data are provided as a Source Data file.

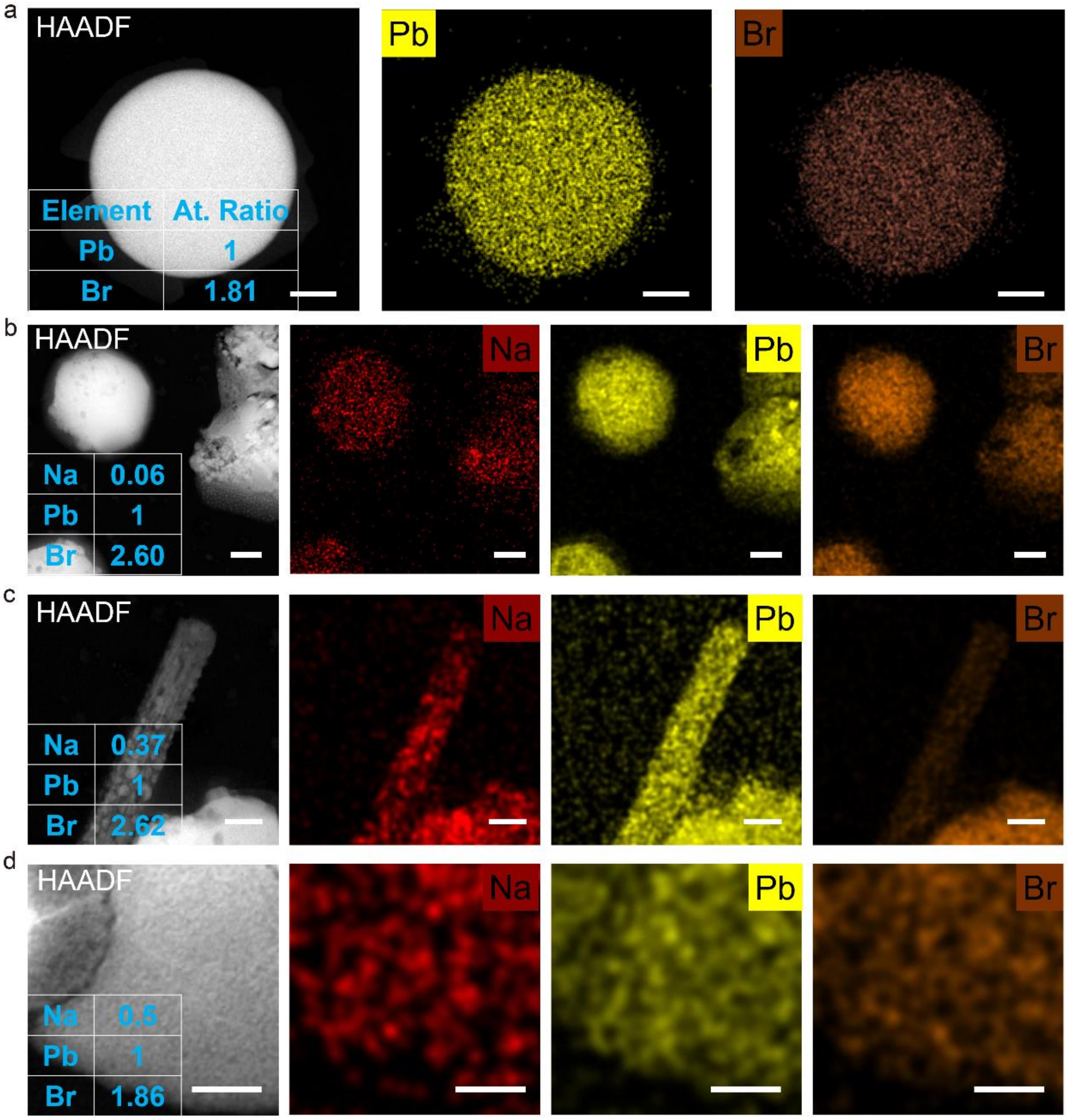

Supplementary Fig. 11 EDS spectrum analysis of pure $PbBr_2$ and substoichiometric Na_xPbBr_y phases. **a-d**, HAADF-STEM image and corresponding EDS elemental maps of pure $PbBr_2$ (**a**), and three typical substoichiometric intermediates: $Na_{0.06}PbBr_{2.60}$ with a particle-like morphology (**b**), $Na_{0.37}PbBr_{2.62}$ with a quasi-layered film morphology (**c**), and $Na_{0.5}PbBr_{1.86}$ with a fully layered film morphology (**d**). Scale bars, 200 nm (**a**), 100 nm (**b-d**).

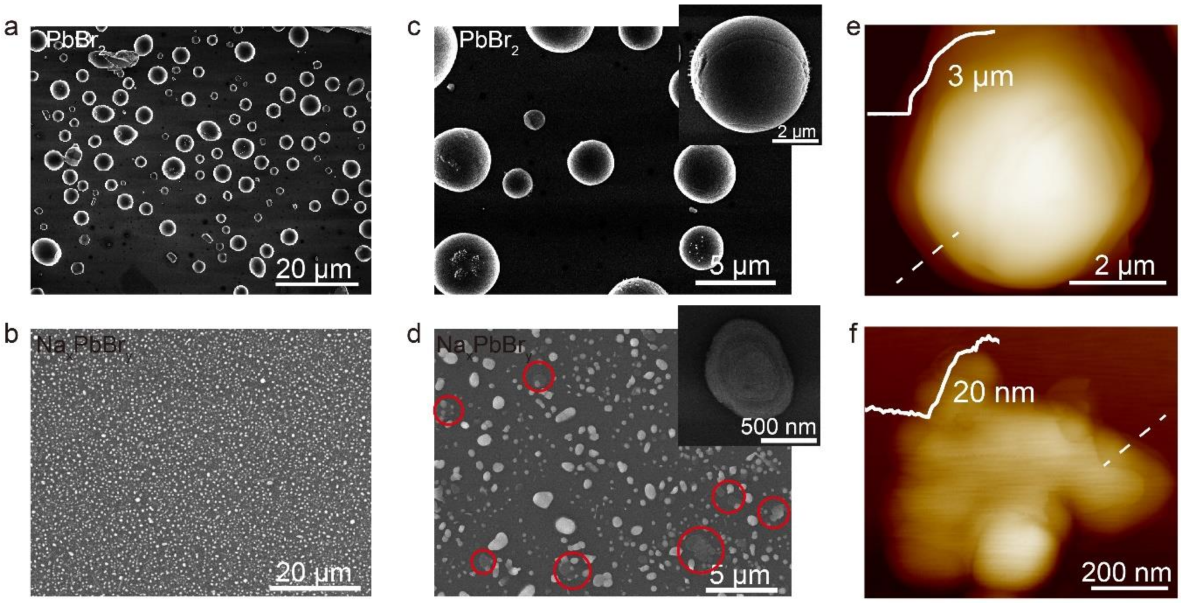

Supplementary Fig. 12 Characterization of Na_xPbBr_y seeding layer and $PbBr_2$ layer. **a**,**b,** Comparative scanning electron microscope (SEM) images of $PbBr_2$ (**a**) and Na_xPbBr_y (**b**). **c**,**d,** Enlarged SEM images of $PbBr_2$ (**c**) and Na_xPbBr_y (**d**), in which 2D Na_xPbBr_y flakes are visible in the area circled by red circles. **e**,**f,** Comparative atomic force microscope (AFM) images of $PbBr_2$ (**e**) and Na_xPbBr_y (**f**). Source data are provided as a Source Data file.

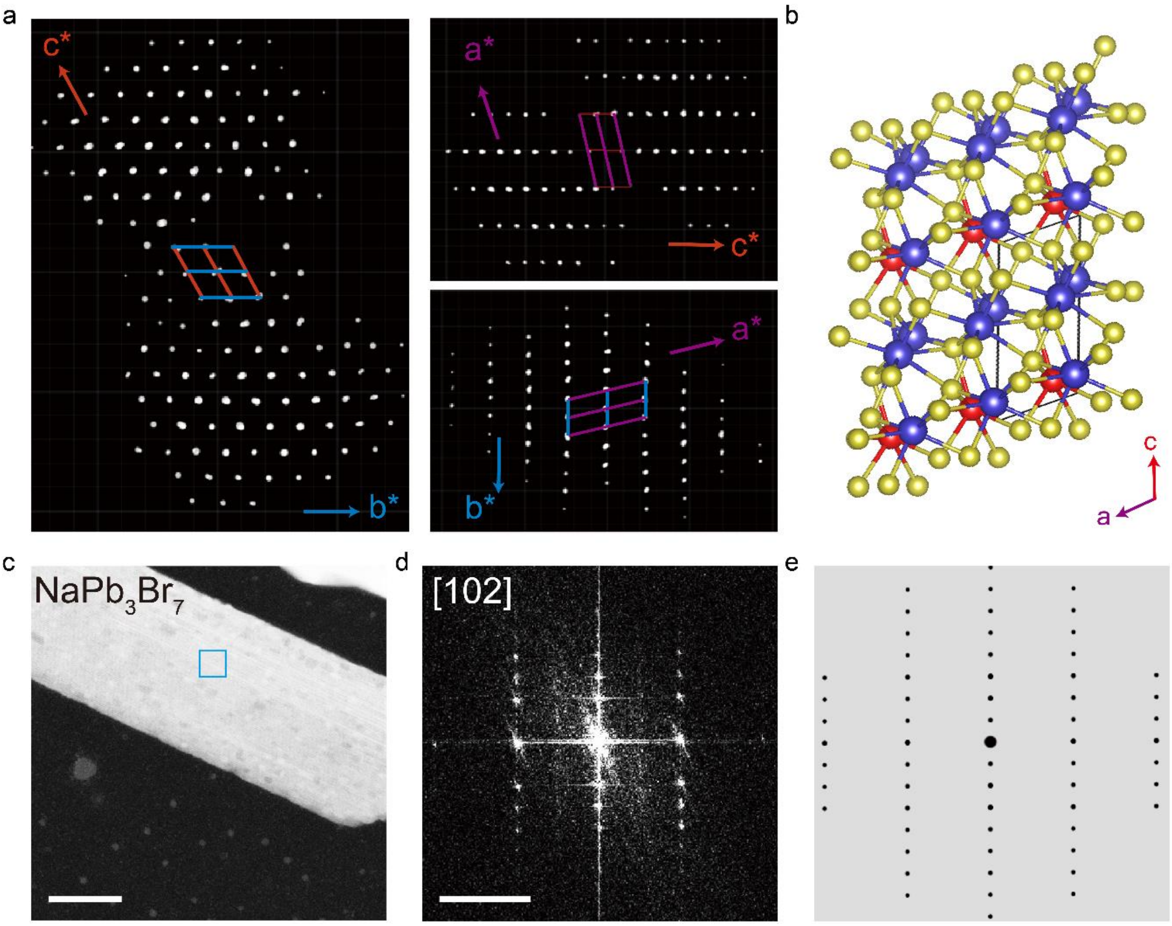

Supplementary Fig. 13 3D electron diffraction measurement of $NaPb_3Br_7$ compared to the atomic structure of $PbBr_2$. a, Projection views of the reconstructed 3D reciprocal lattice of $NaPb_3Br_7$ along the *a**, *b**, and *c** axes, respectively. The 3D electron diffraction data yields the following crystal cell parameters: $a = 4.31$ Å, $b = 9.23$ Å, $c = 9.34$ Å, $\alpha = 111.27°$, $\beta = 103.92°$, $\gamma = 103.21°$. **b**, Resolved atomic structure model of $NaPb_3Br_7$ viewed along [010] axes. **c**, Low-magnification HAADF image of $NaPb_3Br_7$. The atomic-resolution HAADF image shown in Fig. 3g was captured from the blue box region in (**c**). **d**,**e**, FFT pattern (**d**) of the HAADF image of $NaPb_3Br_7$ in Fig. 3g, showing perfect agreement with the simulated reciprocal lattice (**e**) of $NaPb_3Br_7$, derived from the 3D electron diffraction refined crystal structure along the [102] crystal orientation. Scale bars, 50 nm (**c**), 5 nm (**d**).

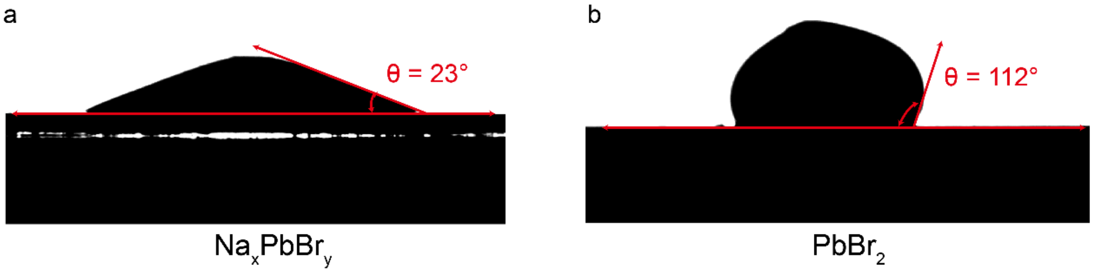

Supplementary Fig. 14 Contact angle of Na_xPbBr_y (a) and $PbBr_2$ (b) on SiO_2/Si substrate.

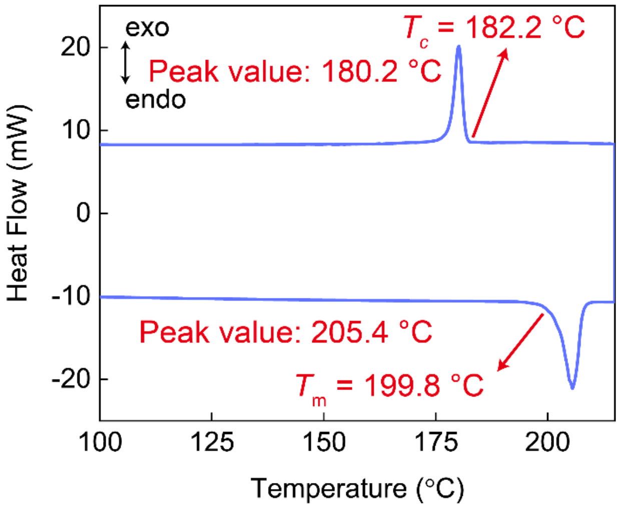

Supplementary Fig. 15 Heating-cooling DSC scans of Na_xPbBr_y interacting with BABr. Source data are provided as a Source Data file.

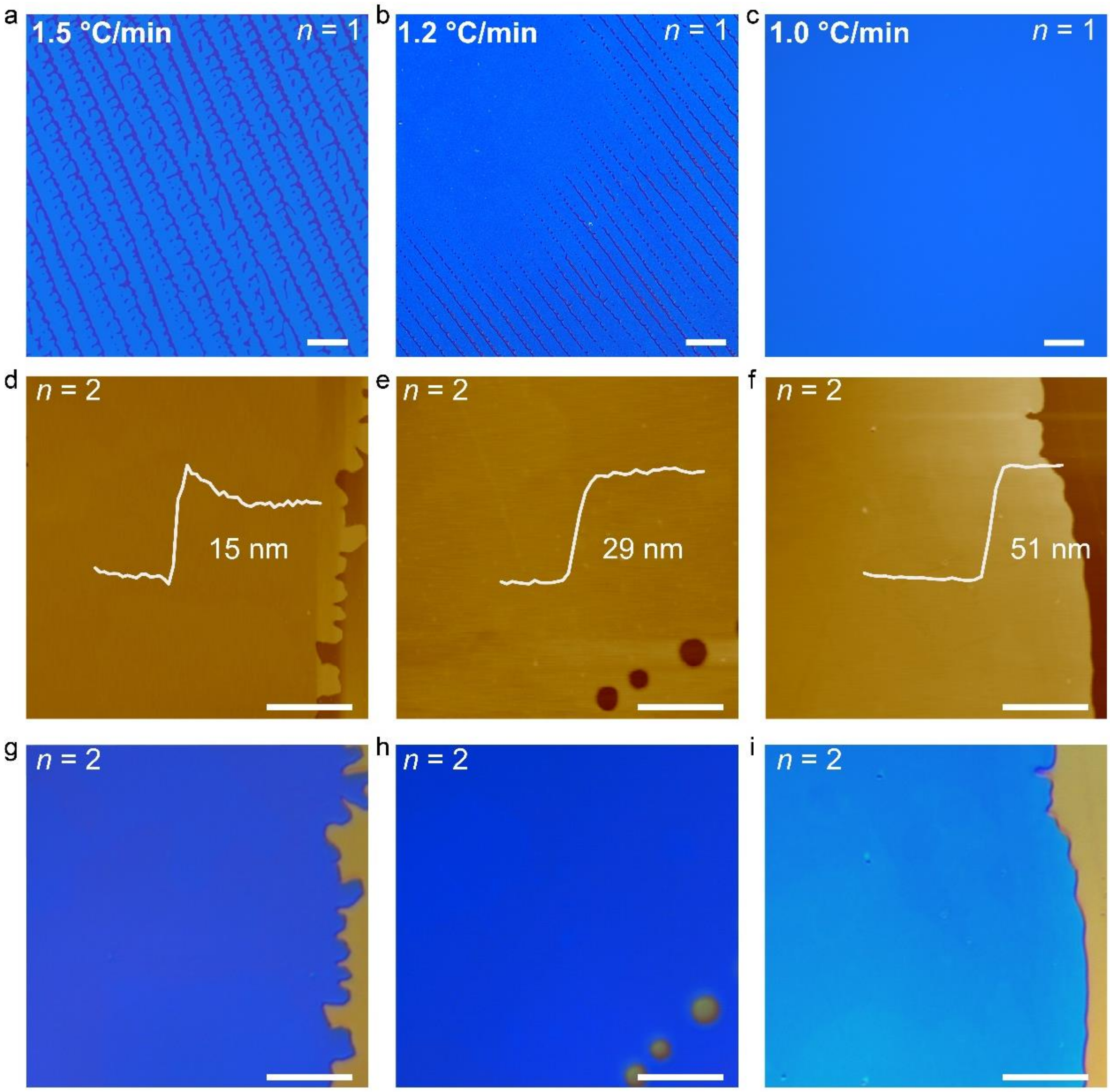

Supplementary Fig. 16 Controlled growth of single-crystalline *n* = 1 $(BA)_2PbBr_4$ and *n* = 2 $(BA)_2(MA)Pb_2Br_7$ films. a-c, Influence of cooling rate on HOIP morphology formation on SiO_2/Si. The HOIP morphology transitions from dendrites structures to film as the cooling rate decreases from 1.5 ºC/min to 1.0 ºC/min. **d-f**, Thickness-dependent growth of *n* = 2 $(BA)_2(MA)Pb_2Br_7$ films, characterized by AFM, showing film thickness of 15 nm (**d**), 29 nm (**e**) and 51 nm (**f**). **g-i**, Corresponding thickness-dependent optical images associated with (**d-f**), respectively. Scale bars, 10 µm (**a-i**). Source data are provided as a Source Data file.

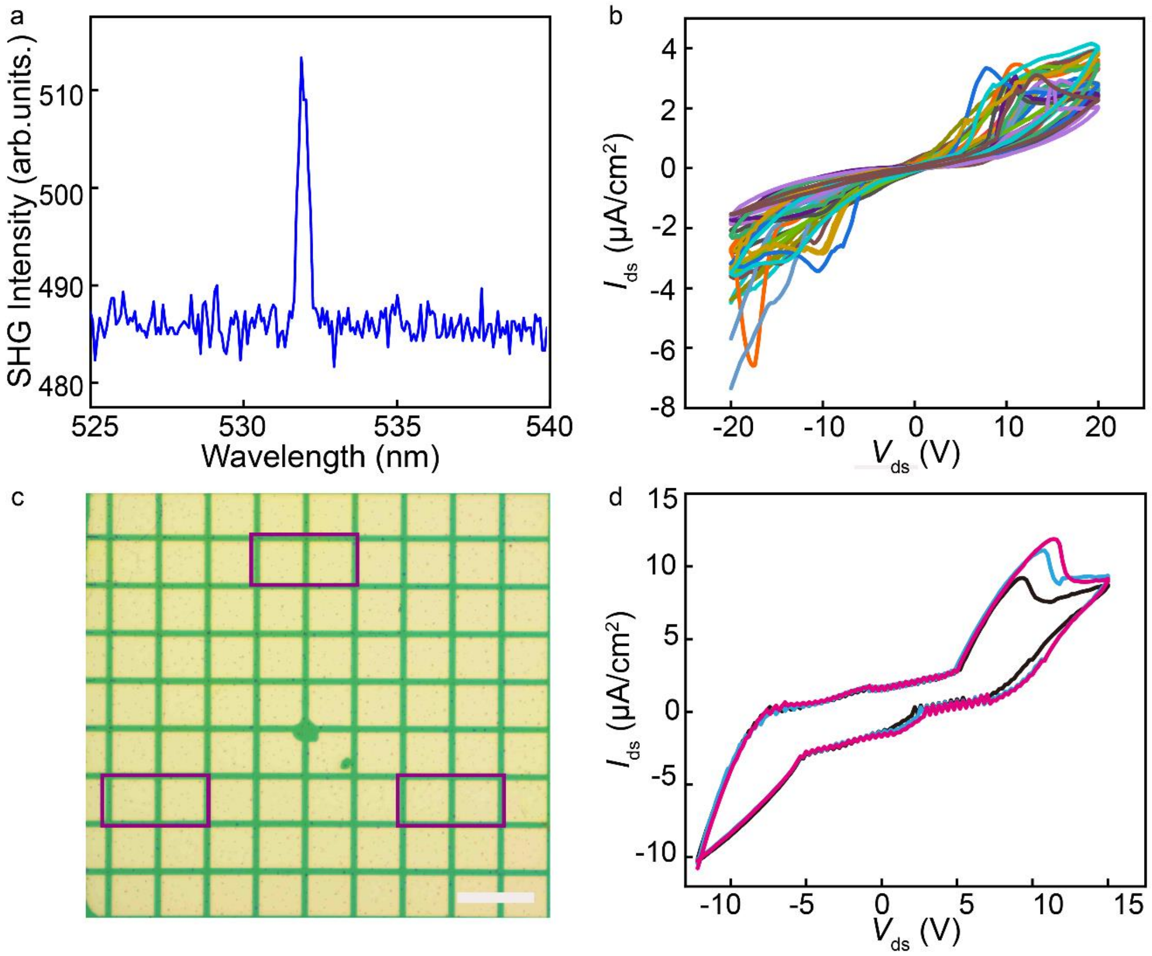

Supplementary Fig. 17 Electronic characterization of *n* = 2 HOIP ferroelectric films. a, SHG measurement on the *n* = 2 film. **b**, 20 sets of corresponding I_{ds}-V_{ds} curves, as shown in Fig. 5h. **c**, Optical image of large-area two-terminal arrays directly fabricated on the grown *n* = 2 film with a thickness of 75 nm on SiO_2/Si. **d**, I_{ds}-V_{ds} curves measured for the three pairs of electrodes highlighted by the purple box in (**c**). Scale bar, 200 μm (**c**). Source data are provided as a Source Data file.

Supplementary Table 1. Crystal structures of grown *n* = 1 and *n* = 2 HOIP films

	$(BA)_2PbBr_4$	$(BA)_2(MA)Pb_2Br_7$
Formula	$C_8H_{24}Br_4N_2Pb$	$C_9H_{30}Br_7N_3Pb_2$
n	1	2
Formula Weight, g/mol	675.12	1154.11
Crystal Structure	Orthorhombic	Orthorhombic
Space group No.	61	36
Space group	*Pbca*	$Cmc2_1$
a (Å)	8.268	8.4
b (Å)	8.385	8.35
c (Å)	27.7	39.35
$\alpha = \beta = \gamma$ (°)	90	90
Volume (Å^3)	1920.36	2760.0

Supplementary Table 2. Comparison of the E_c value of our film with the reported E_c values of solution-processed polycrystalline films.

HOIPs component	E_c value	Ref.
$(PFBA)_2PbBr_4$	~25 kV/cm	*(3)*
$(AP)RbBr_3$	~45 kV/cm	(*4*)
$(4,4\text{-}DFHHA)_2PbI_4$	~300 V	(*5*)
$(2\text{-}FBA)_2PbCl_4$	~15 kV/cm	(*6*)
$(ATHP)_2PbBr_4$	~40 kV/cm	(*7*)
$(BA)_2(MA)Pb_2Br_7$	10.2 kV/cm	This work

Supplementary References

1. Li, C. *et al*. Phonon driven ferroelectricity and Raman active modes in hybrid organic-inorganic perovskites. *Adv. Mater.* **37**, 2419685 (2025).

2. Dhanabalan, B. *et al*., Directional anisotropy of the vibrational modes in 2D-layered perovskites. *ACS Nano* **14**, 4689-4697 (2020).

3. Zhang, H.-Y., Zhang, Z.-X., Song, X.-J., Chen, X.-G. & Xiong, R.-G. Two-dimensional hybrid perovskite ferroelectric induced by perfluorinated substitution. *J. Am. Chem. Soc.* **142**, 20208-20215 (2020).

4. Pan, Q. *et al.* A Three-dimensional molecular perovskite ferroelectric: (3-Ammoniopyrrolidinium)$RbBr_3$. *J. Am. Chem. Soc.* **139**, 3954-3957 (2017).

5. Chen, X.-G. *et al.* Confinement-driven ferroelectricity in a two-dimensional hybrid lead iodide perovskite. *J. Am. Chem. Soc.* **142**, 10212-10218 (2020).

6. Shi, P.-P. *et al.* Two-dimensional organic-inorganic perovskite ferroelectric semiconductors with fluorinated aromatic spacers. *J. Am. Chem. Soc.* **141**, 18334-18340 (2019).

7. Chen, X.-G. *et al.* Two-dimensional layered perovskite ferroelectric with giant piezoelectric voltage coefficient. *J. Am. Chem. Soc.* **142**, 1077-1082 (2020).
