# Supplementary material for: Two-dimensional melt growth of large-scale, single-crystalline hybrid organic-inorganic perovskite films
Source: Nat Commun. 2026 Jun 3;17:7169. doi: 10.1038/s41467-026-73886-4 (PMC13396412; doi:10.1038/s41467-026-73886-4)
Supplement: Supplementary file 2 — Description of Additional Supplementary Files [file 41467_2026_73886_MOESM2_ESM.pdf]

Description of Additional Supplementary Files

File Name: Supplementary Movie 1
Description: In situ recording of the 2D melt growth process of a molecularly thin HOIP film.

File Name: Supplementary Movie 2
Description: In situ recording of the 2D melt growth process of a relatively thick HOIP film.

File Name: Supplementary Movie 3
Description: In situ recording of the conventional growth process of HOIP using $PbBr_2$ as inorganic precursor.

File Name: Supplementary Movie 4
Description: Dendrite formation induced by a fast cooling rate.

File Name: Supplementary Data 1
Description: Initial configurations of Pb octahedron from metadynamics simulation exploring the potential formation of Pb octahedron in the presence of BABr molecules only.

File Name: Supplementary Data 2
Description: Final configurations of Pb octahedron in metadynamics simulation to explore the potential formation of Pb octahedron only with BABr molecules.

File Name: Supplementary Data 3
Description: Initial configurations of Pb octahedron in metadynamics simulations to explore the potential formation of Pb octahedron with BABr molecules under the assistance of Na^+.

File Name: Supplementary Data 4
Description: Final configurations of Pb octahedron in metadynamics simulations to explore the potential formation of Pb octahedron with BABr molecules under the assistance of Na^+.

File Name: Source Data 1
Description: Raw data for the Figures in the main manuscript and Supplementary Information.

File Name: Source Data 2
Description: Source data of unedited TEM images and Figure 2b-d images.
