# Supplementary material for: Two-dimensional melt growth of large-scale, single-crystalline hybrid organic-inorganic perovskite films
Source: Nat Commun. 2026 Jun 3;17:7169. doi: 10.1038/s41467-026-73886-4 (PMC13396412; doi:10.1038/s41467-026-73886-4)
Supplement: Supplementary file 11 — Reporting Summary [file 41467_2026_73886_MOESM11_ESM.pdf]

nature portfolio

Corresponding author(s): Kai Leng

Last updated by author(s): Apr 28, 2026

Reporting Summary

Nature Portfolio wishes to improve the reproducibility of the work that we publish. This form provides structure for consistency and transparency in reporting. For further information on Nature Portfolio policies, see our Editorial Policies and the Editorial Policy Checklist.

Statistics

For all statistical analyses, confirm that the following items are present in the figure legend, table legend, main text, or Methods section.

n/a	Confirmed	
☐	☒	The exact sample size (*n*) for each experimental group/condition, given as a discrete number and unit of measurement
☐	☒	A statement on whether measurements were taken from distinct samples or whether the same sample was measured repeatedly
☒	☐	The statistical test(s) used AND whether they are one- or two-sided<br>*Only common tests should be described solely by name; describe more complex techniques in the Methods section.*
☒	☐	A description of all covariates tested
☒	☐	A description of any assumptions or corrections, such as tests of normality and adjustment for multiple comparisons
☐	☒	A full description of the statistical parameters including central tendency (e.g. means) or other basic estimates (e.g. regression coefficient) AND variation (e.g. standard deviation) or associated estimates of uncertainty (e.g. confidence intervals)
☒	☐	For null hypothesis testing, the test statistic (e.g. *F*, *t*, *r*) with confidence intervals, effect sizes, degrees of freedom and *P* value noted<br>*Give P values as exact values whenever suitable.*
☒	☐	For Bayesian analysis, information on the choice of priors and Markov chain Monte Carlo settings
☒	☐	For hierarchical and complex designs, identification of the appropriate level for tests and full reporting of outcomes
☒	☐	Estimates of effect sizes (e.g. Cohen's *d*, Pearson's *r*), indicating how they were calculated

Our web collection on statistics for biologists contains articles on many of the points above.

Software and code

Policy information about availability of computer code

Data collection	The data were collected using the following instruments: Dual-zone tubular furnace equipped with optical microscope (Micro-STS1200, Units Technology), Leica DM2700 M microscope and Nikon ECLIPSE LV150N microscope, X-ray diffractometer (Rigaku SmartLab), Confocal Raman microscopy (WITec alpha300R), Synchrotron-based grazing incidence X-ray diffractometer (PLS-II 6D UNIST-PAL beamline), Scanning probe microscope (Asylum MFP-3D Infinity), Annular dark field-scanning transmission electron microscope (JEOL ARM200F), High-resolution transmission electron microscope (Titan Krios and Talos F200X G2), Atomic force microscope (Bruker Dimension ICON), Field-emission scanning electron microscope (FE-SEM, Tescan MAIA3), X-ray photoelectron spectrometer (Thermo Fisher Scientific Nexsa), Contact angle tester (OCA25-HTV1800 system), Thermal gravimetric analyzer (Mettler Toledo TGA/DSC3+), Differential scanning calorimetry (Mettler Toledo DSC3), Semiconductor tester (Keithley 4200), TF ANALYZER 3000 system (aixACCT System GmbH).
Data analysis	The data were analyzed and plotted using Origin 2018. The Schematic illustration is created with 3D Max. WITec Project Five 5.0 was used to process the Raman, photoluminescence (PL), and SHG data. DigitalMicrograph was used to analyze the TEM images.

For manuscripts utilizing custom algorithms or software that are central to the research but not yet described in published literature, software must be made available to editors and reviewers. We strongly encourage code deposition in a community repository (e.g. GitHub). See the Nature Portfolio guidelines for submitting code & software for further information.

Data

Policy information about availability of data

All manuscripts must include a data availability statement. This statement should provide the following information, where applicable:

- Accession codes, unique identifiers, or web links for publicly available datasets
- A description of any restrictions on data availability
- For clinical datasets or third party data, please ensure that the statement adheres to our policy

The data that support the findings of this study are available within the paper. Additional data are available from the corresponding authors upon request.

Research involving human participants, their data, or biological material

Policy information about studies with human participants or human data. See also policy information about sex, gender (identity/presentation), and sexual orientation and race, ethnicity and racism.

Reporting on sex and gender	Not applicable
Reporting on race, ethnicity, or other socially relevant groupings	Not applicable
Population characteristics	Not applicable
Recruitment	Not applicable
Ethics oversight	Not applicable

Note that full information on the approval of the study protocol must also be provided in the manuscript.

Field-specific reporting

Please select the one below that is the best fit for your research. If you are not sure, read the appropriate sections before making your selection.

[x] Life sciences [ ] Behavioural & social sciences [ ] Ecological, evolutionary & environmental sciences

For a reference copy of the document with all sections, see nature.com/documents/nr-reporting-summary-flat.pdf

Life sciences study design

All studies must disclose on these points even when the disclosure is negative.

Sample size	Not applicable
Data exclusions	Not applicable
Replication	Not applicable
Randomization	Not applicable
Blinding	Not applicable

Reporting for specific materials, systems and methods

We require information from authors about some types of materials, experimental systems and methods used in many studies. Here, indicate whether each material, system or method listed is relevant to your study. If you are not sure if a list item applies to your research, read the appropriate section before selecting a response.

Materials & experimental systems

n/a	Involved in the study
☒	☐ Antibodies
☒	☐ Eukaryotic cell lines
☒	☐ Palaeontology and archaeology
☒	☐ Animals and other organisms
☒	☐ Clinical data
☒	☐ Dual use research of concern
☒	☐ Plants

Methods

n/a	Involved in the study
☒	☐ ChIP-seq
☒	☐ Flow cytometry
☒	☐ MRI-based neuroimaging

Plants

Seed stocks	Not applicable
Novel plant genotypes	Not applicable
Authentication	Not applicable
