# Supplementary figures and images for: Two-dimensional melt growth of large-scale, single-crystalline hybrid organic-inorganic perovskite films
Source: Nat Commun. 2026 Jun 3;17:7169. doi: 10.1038/s41467-026-73886-4 (PMC13396412; doi:10.1038/s41467-026-73886-4)

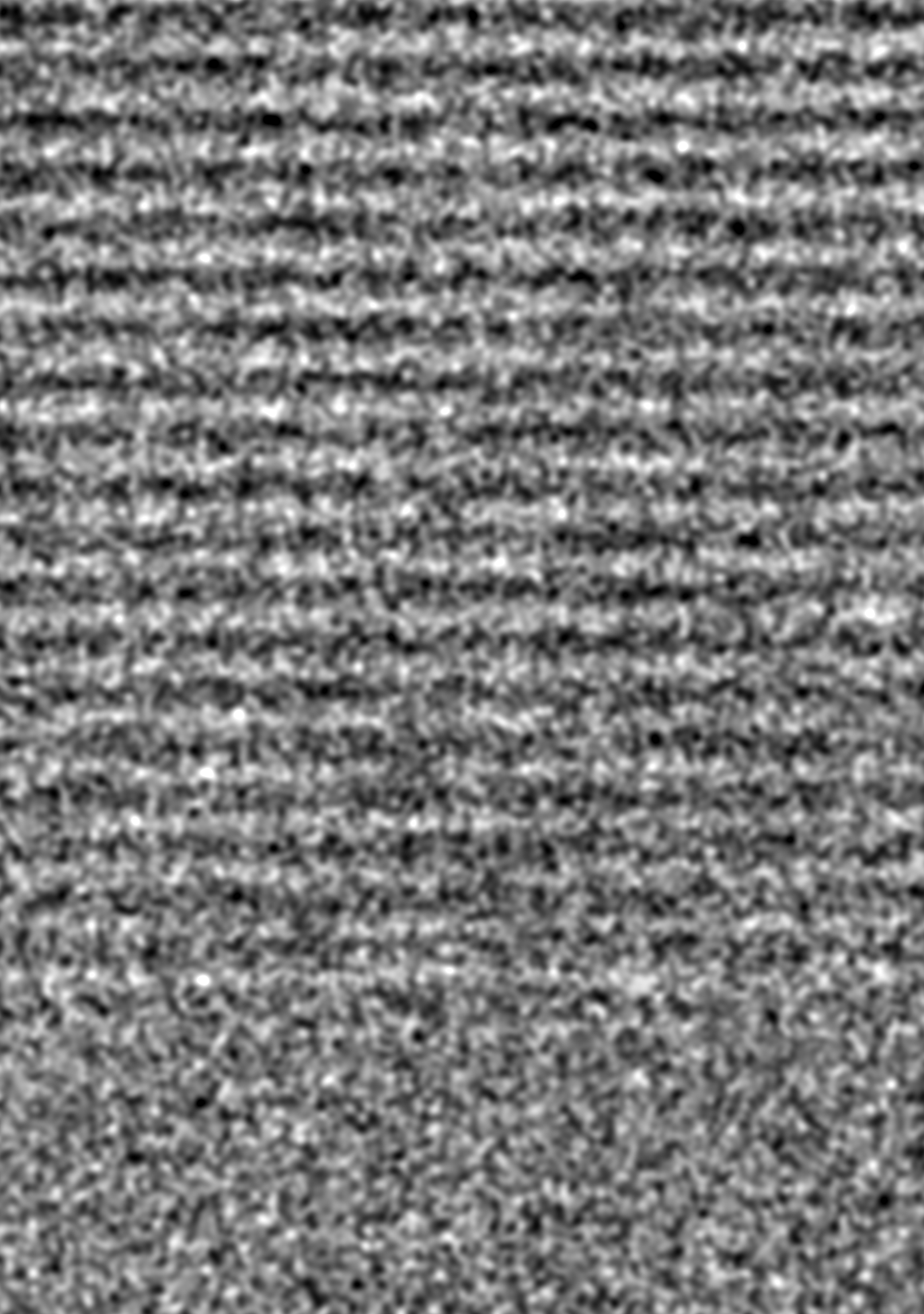

Supplement: Supplementary file 14 — Source Data 2 [file 41467_2026_73886_MOESM14_ESM.zip › Source data 2/fig.1e_grey.tif]

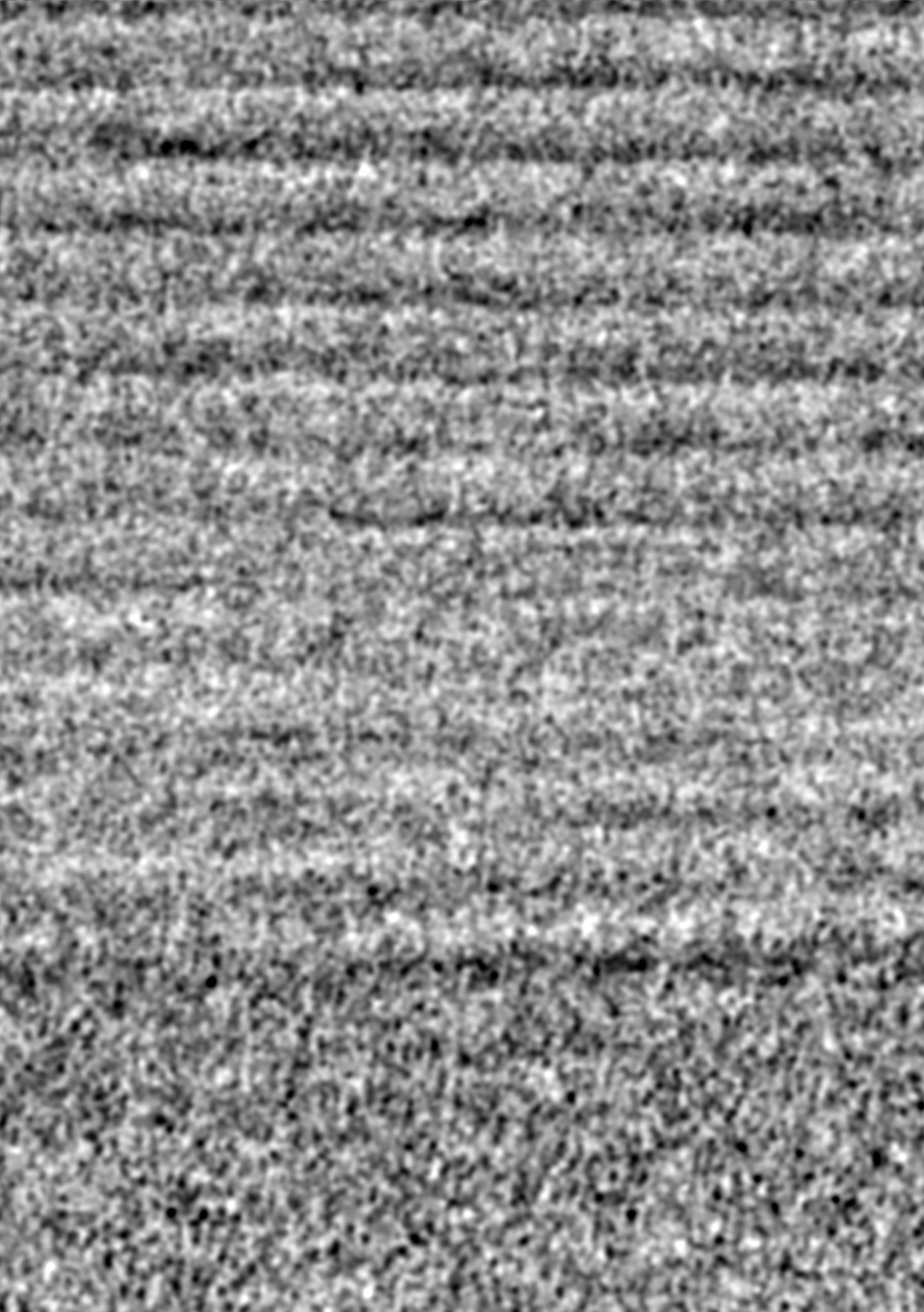

Supplement: Supplementary file 14 — Source Data 2 [file 41467_2026_73886_MOESM14_ESM.zip › Source data 2/Fig.1f_grey.tif]

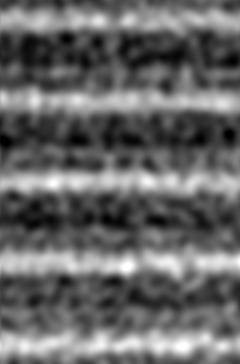

Supplement: Supplementary file 14 — Source Data 2 [file 41467_2026_73886_MOESM14_ESM.zip › Source data 2/fig.1g_grey.tif]

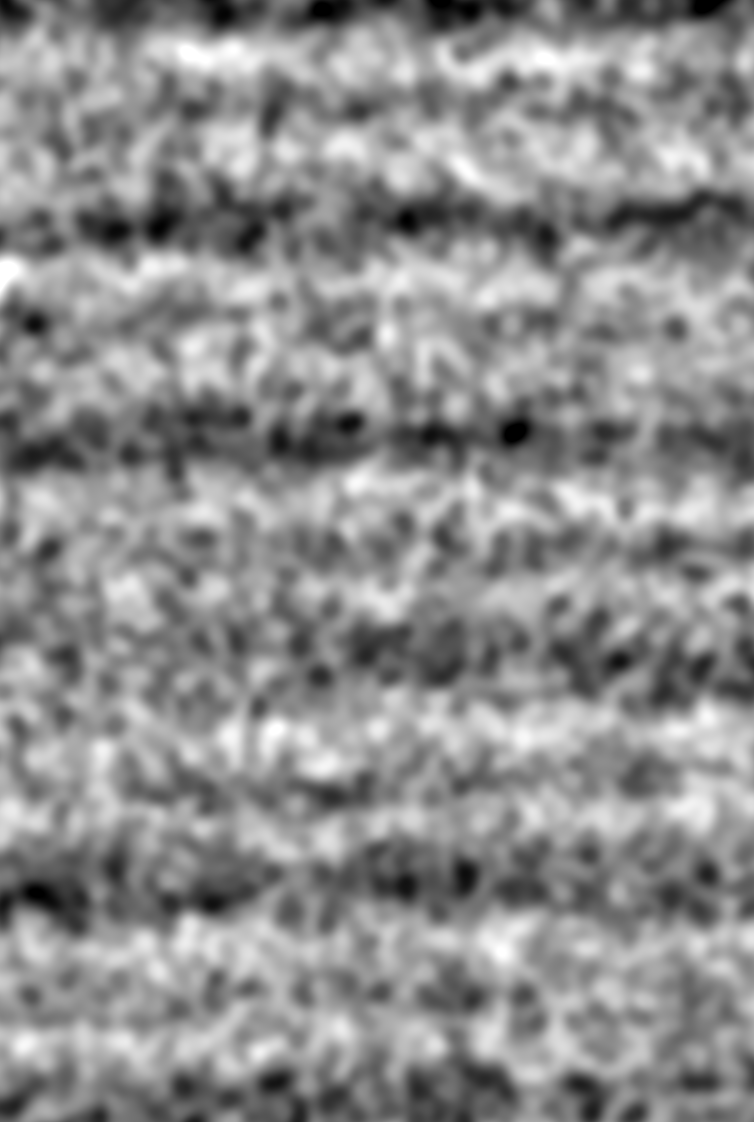

Supplement: Supplementary file 14 — Source Data 2 [file 41467_2026_73886_MOESM14_ESM.zip › Source data 2/Fig.1h_grey.tif]

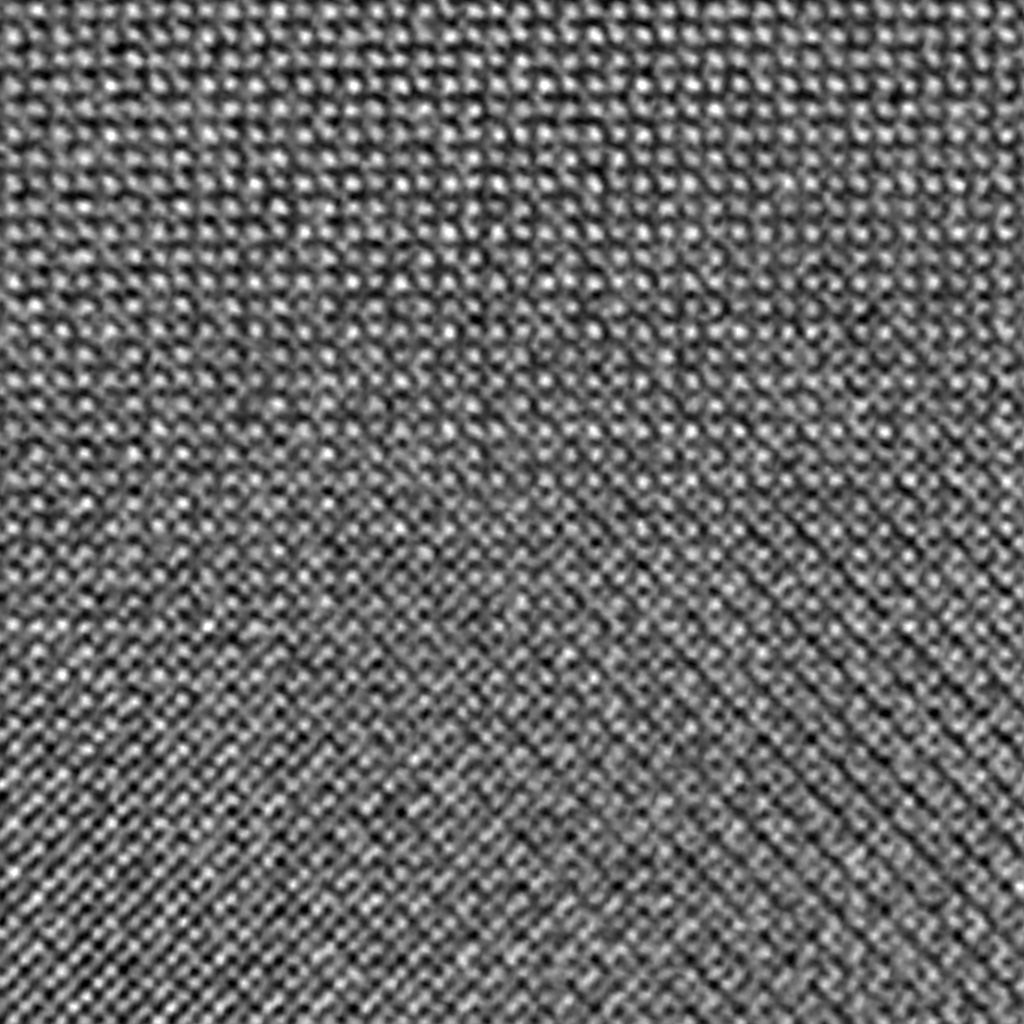

Supplement: Supplementary file 14 — Source Data 2 [file 41467_2026_73886_MOESM14_ESM.zip › Source data 2/fig.1i_grey.tif]

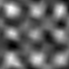

Supplement: Supplementary file 14 — Source Data 2 [file 41467_2026_73886_MOESM14_ESM.zip › Source data 2/fig.1i_inset_grey.tif]

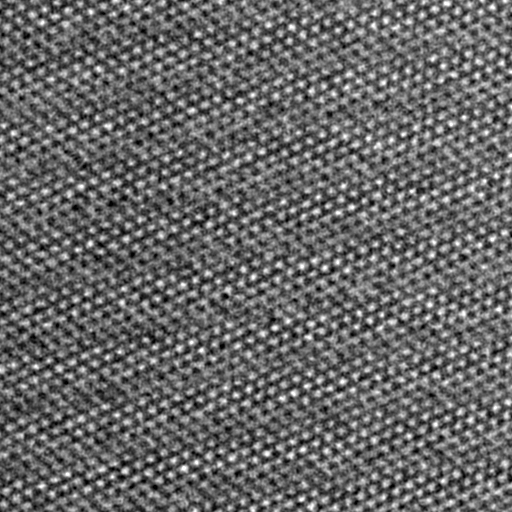

Supplement: Supplementary file 14 — Source Data 2 [file 41467_2026_73886_MOESM14_ESM.zip › Source data 2/fig.3c_grey.tif]

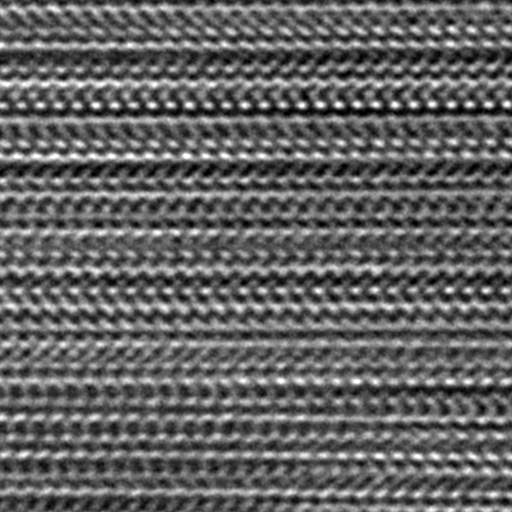

Supplement: Supplementary file 14 — Source Data 2 [file 41467_2026_73886_MOESM14_ESM.zip › Source data 2/fig.3d_grey.tif]

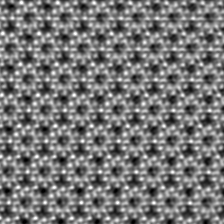

Supplement: Supplementary file 14 — Source Data 2 [file 41467_2026_73886_MOESM14_ESM.zip › Source data 2/fig.3e_grey.tif]

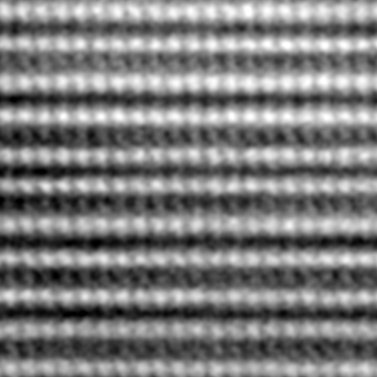

Supplement: Supplementary file 14 — Source Data 2 [file 41467_2026_73886_MOESM14_ESM.zip › Source data 2/fig.3g_grey.tif]

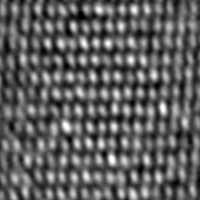

Supplement: Supplementary file 14 — Source Data 2 [file 41467_2026_73886_MOESM14_ESM.zip › Source data 2/fig.3i_grey.tif]

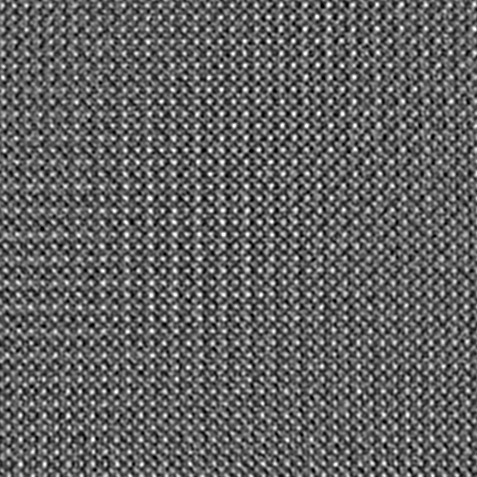

Supplement: Supplementary file 14 — Source Data 2 [file 41467_2026_73886_MOESM14_ESM.zip › Source data 2/fig.S_N2_inplane_grey.tif]

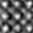

Supplement: Supplementary file 14 — Source Data 2 [file 41467_2026_73886_MOESM14_ESM.zip › Source data 2/fig.S_N2_inset_inplane_grey.tif]

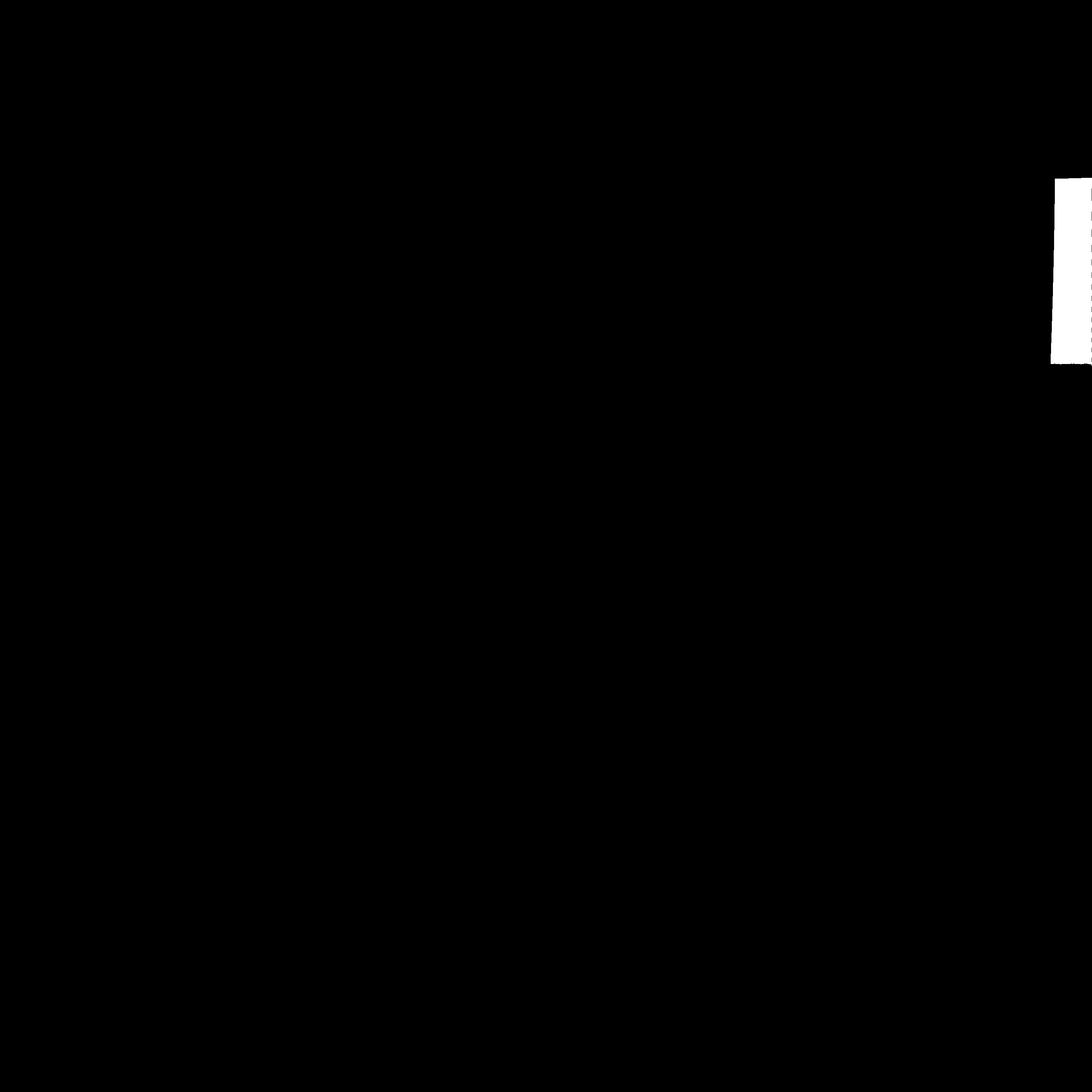

Supplement: Supplementary file 14 — Source Data 2 [file 41467_2026_73886_MOESM14_ESM.zip › Source data 2/Figure 2b-Rot0d_1s_0003.tif]

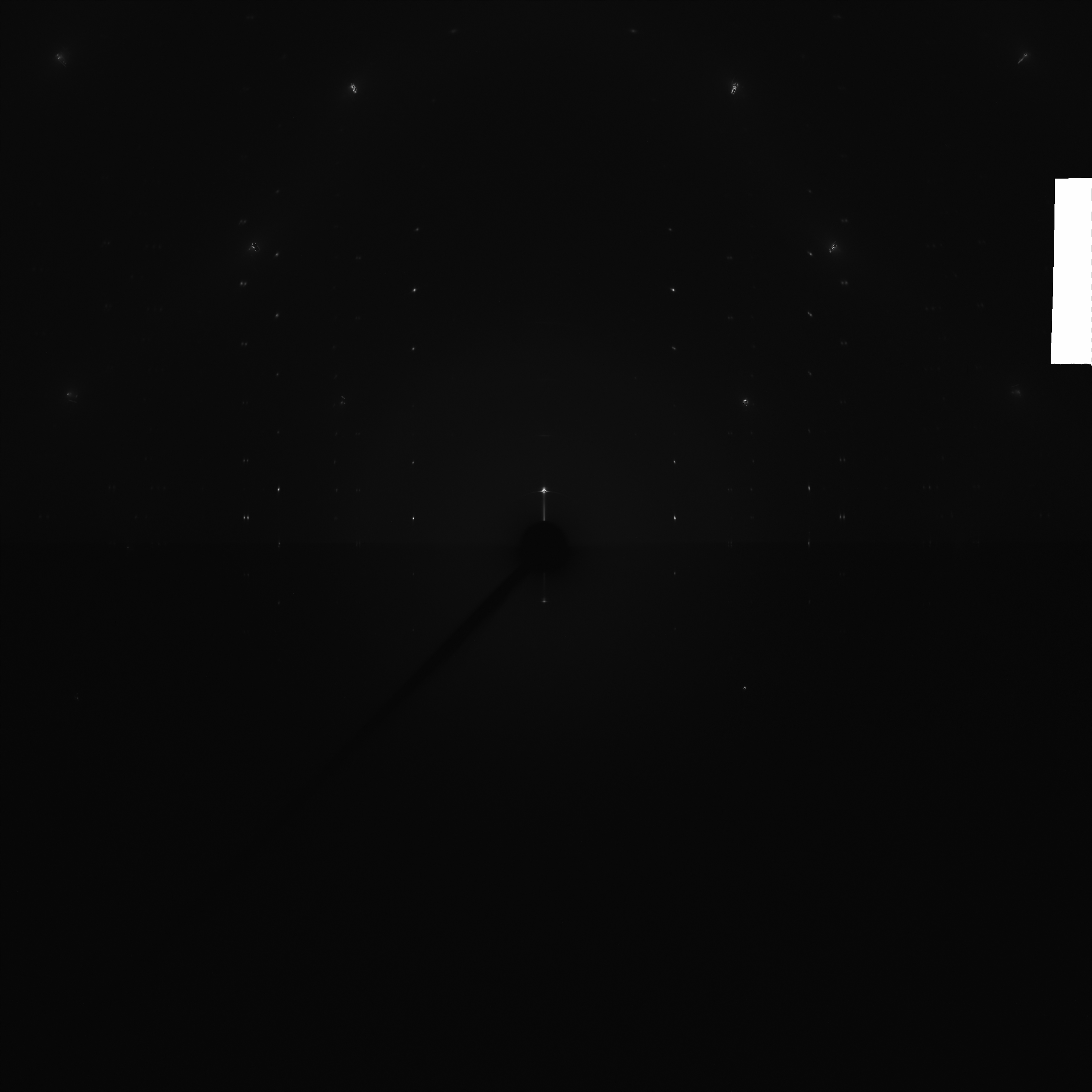

Supplement: Supplementary file 14 — Source Data 2 [file 41467_2026_73886_MOESM14_ESM.zip › Source data 2/Figure 2c-S1_Rot0-180o_merge_0001.tif]
