# Supplementary material for: Two-dimensional melt growth of large-scale, single-crystalline hybrid organic-inorganic perovskite films
Source: Nat Commun. 2026 Jun 3;17:7169. doi: 10.1038/s41467-026-73886-4 (PMC13396412; doi:10.1038/s41467-026-73886-4)
Supplement: Supplementary file 12 — Transparent Peer Review file [file 41467_2026_73886_MOESM12_ESM.pdf]

nature portfolio

Peer Review File

Two-dimensional melt growth of large-scale, single-crystalline hybrid organic-inorganic perovskite films

Corresponding Author: Professor Kai Leng

Parts of this Peer Review File have been redacted as indicated to remove third-party material.

This file contains all reviewer reports in order by version, followed by all author rebuttals in order by version.

Version 0:

Reviewer comments:

Reviewer #1

(Remarks to the Author)
This is a beautiful work reporting the growth of wafter scale single crystalline 2D and quasi-2D halide perovskite thin films. Specifically, the authors developed a pre-deposited inorganic NaxPbBry seeding layer and then treat the seeding layer with organic precursor flux to produce the 2D molten phase of perovskites. The method is very novel, and the results are convincing. Growing wafer scale thin 2D perovskites has been very challenging. This new method provides a promising solution for future device integration. I truly enjoyed reading this work and would enthusiastically recommend its publication.

A few minor questions for the authors to consider:

1. It seems that Pb(OH)2 and NaBr are produced as by-products. Where do the residual materials locate? Below the perovskite layer? Or they are eventually fully converted to 2D perovskites?

2. It is amazing to see Pb Br n=2 phase can be grown. I wonder if this method works for Pb - I or Sn based 2D perovskites? Also, if other aromatic bulky cation can be used? If not, some discussion about why would be helpful for the reader.

3. Have the authors compared with optical and electrical properties of the large sheets with conventional small size 2D crystals? How's PL intensity, defect density, and carrier mobility different from those smaller crystals?

Reviewer #2

(Remarks to the Author)
The manuscript presents a two-dimensional melt growth method based on a NaxPbBry seeding layer. Using this method, large-area, thickness-controlled, single-crystalline HOIP films (n=1, 2) were synthesized on SiO_2/Si substrates. The manuscript concludes by demonstrating the successful implementation of these films in ferroelectric devices. This strategy ingeniously overcomes the long-standing challenges of substrate limitation and phase segregation in HOIP film growth. However, clarifications and careful revisions are needed before the manuscript can be considered for publication.

1. The manuscript mentions that film thickness can be controlled by adjusting the distance between Zone 1 and Zone 2. Please elaborate on the underlying physical mechanism.
2. It is recommended to provide more direct experimental evidence or theoretical computational support (e.g., contact angle simulations or interfacial energy calculations) in the main text or supplementary information to compare the wettability of the NaxPbBry film versus $PbBr_2$.
3. Will the byproducts (NaBr, Pb(OH)2) affect the growth of HOIP crystal? Where will these byproducts be located? Any evidence they are completely removed?
4. The X-axis (coordination number) range in Fig. 3j is only 0-5, yet the text (lines 234-238) discusses structures with coordination numbers of 6 (NaxPbBry) and 7 (PbBr2). Please verify and ensure consistency between the figure and the textual description.

5. How were the optical images in Fig. 4a-c obtained during the high-temperature growth process? Furthermore, optical images alone are not conclusive evidence for a liquid state. Are there supplementary data confirming the transition of NaxPbBry into a liquid phase at 210°C?
6. Please clarify in the caption of Fig. 4g whether the "Nucleation energy" and "Interfacial energy" profiles are qualitative schematic illustrations or quantitative results derived from theoretical calculations.
7. How was the cooling rate mentioned around line 270 precisely controlled? While the Methods section lists specific temperature programs and cooling rates, it is suggested to briefly explain the experimental basis or optimization process for selecting these particular parameters.
8. To better understand the "complete wetting" behavior, could the authors provide additional characterization data for the seeding layer itself (e.g., SEM, XPS, AFM surface morphology) to illustrate its uniformity, coverage, and bonding state with the substrate?
9. The feasibility of growing BA/HA-based 2D single crystal films with n=3 and n=4 should be explored, which would help to demonstrate the broader applicability of the growth technique.
10. In the Conclusion, based on the successful (BA/HA)-based bromide systems, it would be beneficial to more specifically discuss the potential challenges in extending this method to other HOIP systems (e.g., iodides, Dion-Jacobson phases). A brief discussion on the current limits, precision of thickness control, and potential future improvements would also be valuable.

Version 1:

Reviewer comments:

Reviewer #1

(Remarks to the Author)
Authors have carefully revised the manuscript based on reviewers' feedback. The quality and clarity of the manuscript have been greatly improved. I want to congratulate the authors for the impressive results. In my opinion this paper can be accepted and published now.

Reviewer #2

(Remarks to the Author)
The authors have performed additional experiments, particularly the in-situ optical microscopy, DSC measurements, which have substantially strengthened the manuscript. However, after carefully examining the revised materials, two issues remain that require further clarification.
1. The authors provide contact angle measurements to demonstrate the superior wettability of NaxPbBry (35.09°) compared to PbBr2 (112.59°). However, the droplets appear highly irregular in shape, and the contact angle is clearly asymmetric: the left side exhibits a visibly larger angle than the right side. The reported value of 35.09° was taken from the right side, which shows a smaller angle. This selective reporting is problematic, as the true contact angle for an irregular droplet is ambiguous. The authors should provide additional contact angle measurements (at least 3–5 independent droplets) to ensure reliability. Alternatively, if the irregularity stems from the measurement technique, a more controlled method should be considered.
2. The authors successfully grew (BA)2(MA)2Pb3Br10 (n=3) and (HA)2(MA)Pb2Br7 (n=2), which indeed extends the method's demonstrated versatility. A key claim of the paper is the broad applicability of the growth technique, and the absence of n=4 leaves this claim partially unsubstantiated. The growth of an n=4 film (BA-based) is strongly encouraged. A successful demonstration, even with non-optimized film quality, would significantly strengthen the claim of broad applicability. If the attempt fails, a brief discussion of the observed challenges would still be valuable for the community.

Version 2:

Reviewer comments:

Reviewer #2

(Remarks to the Author)
The authors have addressed all my concerns, and the paper can be accepted.

Open Access This Peer Review File is licensed under a Creative Commons Attribution 4.0 International License, which permits use, sharing, adaptation, distribution and reproduction in any medium or format, as long as you give appropriate credit to the original author(s) and the source, provide a link to the Creative Commons license, and indicate if changes were made.
In cases where reviewers are anonymous, credit should be given to 'Anonymous Referee' and the source.
The images or other third party material in this Peer Review File are included in the article’s Creative Commons license, unless indicated otherwise in a credit line to the material. If material is not included in the article’s Creative Commons license and your intended use is not permitted by statutory regulation or exceeds the permitted use, you will need to obtain permission directly from the copyright holder.
To view a copy of this license, visit https://creativecommons.org/licenses/by/4.0/

A Point-by-Point Response Letter for *Nature Communications* Manuscript NCOMMS-25-78861-T *"Two-dimensional melt growth of large-scale, single-crystalline hybrid organic-inorganic perovskite films"*

Reviewer #1 (Remarks to the Author):

This is a beautiful work reporting the growth of wafter scale single crystalline 2D and quasi-2D halide perovskite thin films. Specifically, the authors developed a pre-deposited inorganic NaxPbBry seeding layer and then treat the seeding layer with organic precursor flux to produce the 2D molten phase of perovskites. The method is very novel, and the results are convincing. Growing wafer scale thin 2D perovskites has been very challenging. This new method provides a promising solution for future device integration. I truly enjoyed reading this work and would enthusiastically recommend its publication.

ANS: Thanks for your positive comment. We have added *in-situ* comparison growth videos of HOIP formation using the Na_xPbBr_y-BABr precursors versus conventional $PbBr_2$-BABr precursors recorded with a newly established CVD system equipped with optical microscopy (Micro-STS1200, Units Technology) (Videos 1–3). These videos further corroborate our previous observations of melting, spreading into a 2D liquid film, and subsequent crystallization into single-crystalline films when using Na_xPbBr_y-BABr precursors.

A few minor questions for the authors to consider:

1. It seems that $Pb(OH)_2$ and NaBr are produced as by-products. Where do the residual materials locate? Below the perovskite layer? Or they are eventually fully converted to 2D perovskites?

ANS: Thank you for this comment. The by-product $Pb(OH)_2$ further decomposes into PbO and remains as a residue in Crucible 1 (Fig. R1a). The initial reaction, $2NaOH + 7PbBr_2 \rightarrow 2NaPb_3Br_7 + Pb(OH)_2$ is carried out at 580 ℃ in Crucible 1. At this temperature, the $Pb(OH)_2$ undergoes thermal decomposition according to $Pb(OH)_2(s) \xrightarrow[400\ ℃]{\Delta} PbO\ (s) + H_2O\ (g)$. The formation of PbO was confirmed by XRD measurement of the residual powder collected from Crucible 1 after the initial reaction (Fig. R1b).

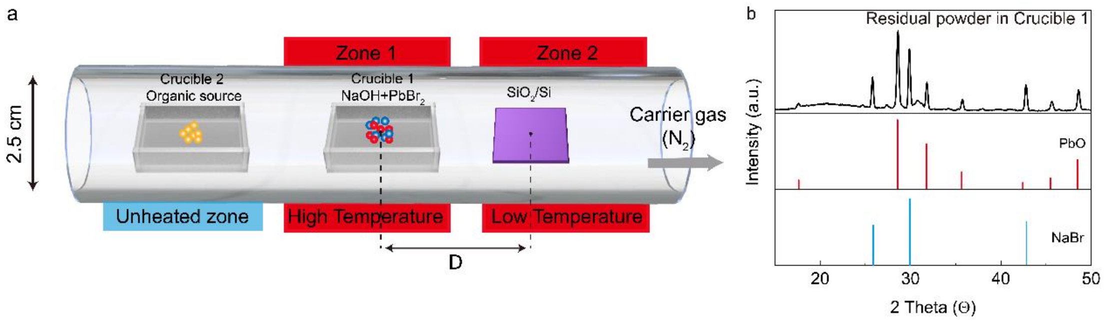

Fig. R1 **a**, CVD growth of HOIP film using Na_xPbBr_y-BABr precursors. **b**, XRD measurement of the residual powder in Crucible 1.

The second interaction proceeds as $NaPb_3Br_7 + 6BABr \rightarrow 3(BA)_2PbBr_4 + NaBr$. The by-

product NaBr is segregated to the edges of the as-grown HOIP films. To determine its distribution, we performed XPS and TEM elemental mapping on as-grown HOIP films with a thickness of ~ 9 nm. Multiple XPS measurements (with an X-ray spot size of ~400 µm) taken from the central region of the film show no detectable Na signal (Fig. R2a,b). In contrast, a weak Na signal is observed at the film edge. Because XPS is a surface-sensitive technique (probing the top ~1–10 nm), we further carried out TEM elemental mapping on HOIP films directly grown on Cu/carbon TEM grids, which allows full transmission through the entire film thickness to bottom. As shown in Fig. R2c-h, no Na signal is detected within the mapped region, indicating that NaBr is not present at or beneath the perovskite layer.

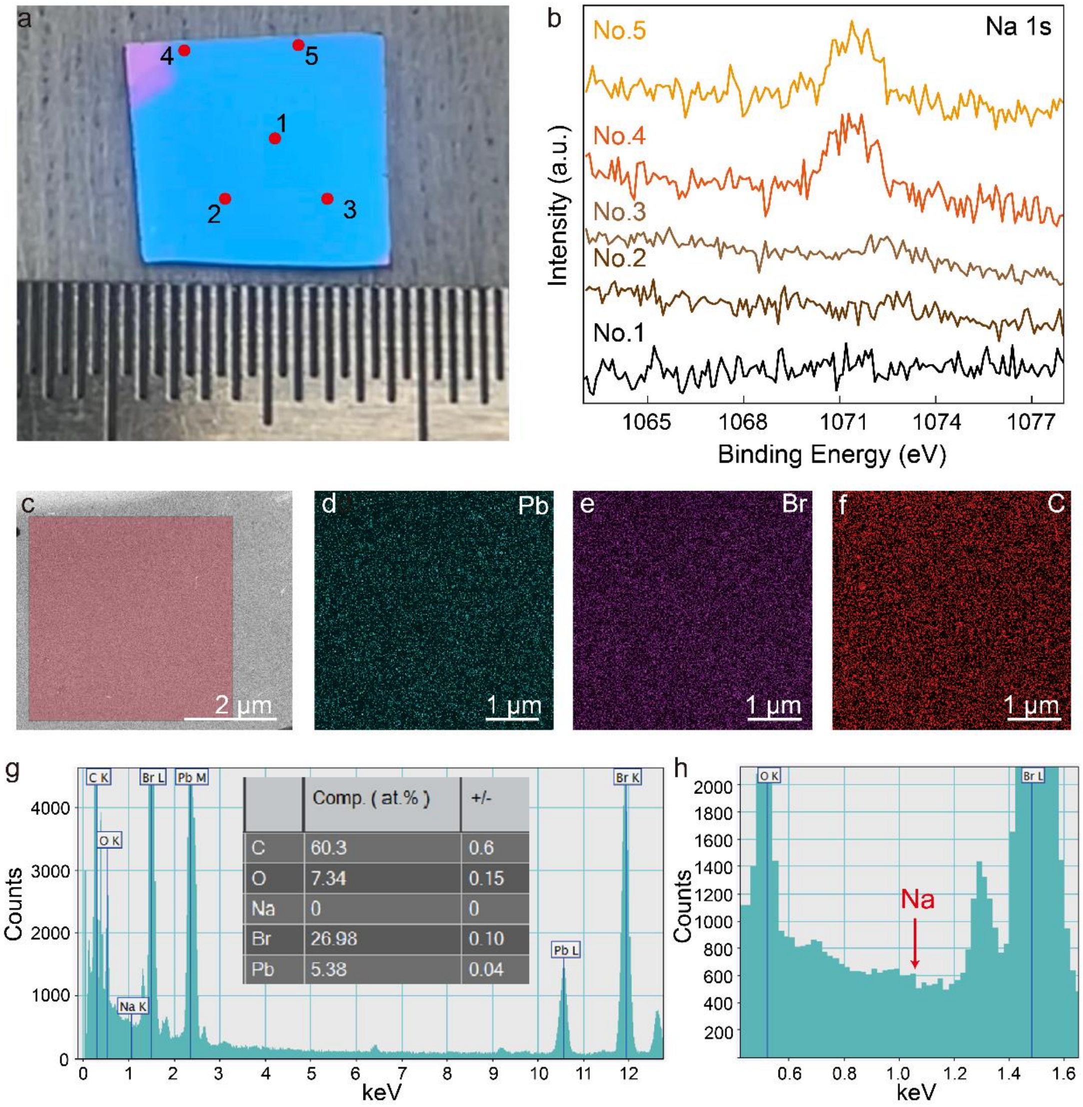

Fig. R2 **Characterization of NaBr by-product.** **a**, Photograph of n = 1 BA_2PbBr_4 single-crystalline film on SiO_2/Si. **b**, Corresponding Na 1s XPS spectra collected at five positions marked in (**a**). **c**, TEM image of BA_2PbBr_4 film grown on a TEM grid. **d-f**, Elemental maps of (**d**) Pb, (**e**) Br and (**f**) C acquired from the red rectangular region in (**c**). **g**, EDS spectrum acquired from the red rectangular region in (**c**). **h**, Zoom-in EDS spectrum on the Na-related energy range.

The NaBr-rich edge regions can be easily removed using a DMF-soaked cotton swab. The films shown in Fig. 1c and 1d in the manuscript correspond to the edges have been removed.

Action: The following description has been added to page 9 of the revised manuscript: "It is worth noting that during the HOIP growth process, the by-product $Pb(OH)_2$ further decomposes into PbO in the crucible of the CVD furnace (Methods). Another by-product, NaBr, is primarily detected at the edges of the as-grown HOIP films (Figure S8) and can be readily removed using a DMF-soaked cotton swab." In addition, Fig. R2 has been added as Fig. S8 in the revised Supplementary Information.

2. It is amazing to see Pb Br n=2 phase can be grown. I wonder if this method works for Pb - I or Sn based 2D perovskites? Also, if other aromatic bulky cation can be used? If not, some discussion about why would be helpful for the reader.

ANS: Thanks for the thoughtful suggestions. The reported 2D melt growth method works for Pb-I based 2D perovskite. We have grown BA_2PbI_4 film, which was verified by XRD and PL measurements (Fig. R3). Besides BA_2PbI_4, we have also added newly grown $(BA)_2(MA)Pb_2Br_7$ ($n = 3$) and $HA_2MAPb_2Br_7$ ($n = 2$), as asked by Reviewer 2 in Question 9.

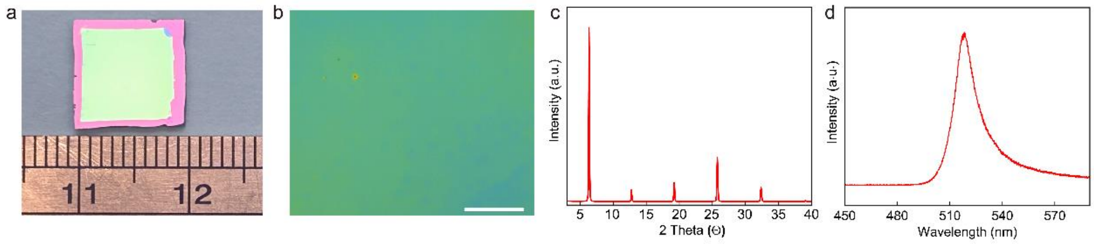

Fig. R3 **Centimetre-scale $(BA)_2PbI_4$ single-crystalline film grown on SiO_2/Si by 2D melt growth. a**, Photograph of $n = 1$ $(BA)_2PbI_4$ film grown on SiO_2/Si. **b**, Zoom-in optical image of $n = 1$ $(BA)_2PbI_4$ film. **c**, XRD pattern. **d**, PL spectrum. Scale bar, 50 μm (**b**).

We also attempted to grow Sn-based 2D perovskites, including BA_2SnI_4 and TEA_2SnI_4. Although melt states can form from the Sn-based precursors, they were not stable due to the sensitivity of Sn^{2+}, and side reactions occurred during crystallization, preventing the formation of large-scale continuous films. A more strictly controlled, oxygen-free growth environment is required to achieve large-scale Sn-based 2D perovskite films which we plan to pursue in future work.

Regarding whether aromatic bulky cation-based HOIPs can be grown by this method, it depends on the three key conditions: 1. Congruent melting of the HOIP precursors; 2. Formation of a continuous 2D liquid film on the substrate; 3. Stable crystallization. Not all HOIPs' precursors can form stable melt states. The concept of "melt state" of HOIPs is recently reported (*Nat. Rev. Mater*. **10**, 211-227 (2025); *J. Am. Chem. Soc*. **146**, 9272-9284 (2024)). Notably, congruent melting temperature (T_m) has recently been reported for chiral *S-/R-*$(NEA)_2PbBr_4$ (T_m = 175 °C) (*Nat. Commun*. **16**, 7696, (2025)). Earlier studies have also reported melting behavior in aromatic bulky-cation-based HOIPs *β*-Me-PEA_2PbI_4 (T_m = 207 °C); 2-F-PEA_2PbI_4 (T_m = 245.8 °C); 4-MeO-PEA_2PbI_4 (T_m = 247.1 °C); PEA_2PbI_4 (T_m = 252.9 °C) (*Chem. Mater.* **29**,

6200–6204 (2017)). These compounds satisfy the first key condition and thus exhibit strong potential for 2D melt growth into large-scale films, provided that appropriate growth parameters meet the remaining two conditions.

Action: The conclusion of the manuscript has been revised as follows: “In addition to HOIPs based on aliphatic chain and cyclic organic cations, HOIPs incorporating aromatic cations can also be grown using our 2D melt-growth approach. Moreover, congruent melting has been reported for chiral NEA-based HOIPs, halogen substituted PEA-based HOIPs, and even 3D perovskites[43-45]. These findings highlight strong potential for extending the 2D melt growth strategy to these systems. Compared to furnace-type CVD, organic molecular beam epitaxy (OMBE) provides a more tunable growth method, affording wafer-scale growth with precise thickness control. Bypassing substrate limitations and enabling parallel production, this work provides a foundation for expanding HOIP-based devices into large-scale industrial applications, including integrated HOIP/Si photovoltaics, photonics and neuromorphic computing platforms.”

Relevant growth description:

“2D melt growth of centimetre-scale *n* = 1 $(BA)_2PbI_4$ film

For the growth of n = 1 $(BA)_2PbI_4$, the entire process was conducted under N_2 gas (99.9%) with a flow rate of 80 sccm. The distance D was set to 19 cm. The organic source used was BAI (35 mg) and a mixture of PbI_2 (30 mg) and NaOH (8 mg) was used as the inorganic source. In step 1, the temperature of Zone 1 was ramped to 520 °C over 25 minutes and maintained at this temperature for 20 minutes. Simultaneously, Zone 2 was heated to 250 °C. In step 2, the temperatures of Zone 1 and Zone 2 were reduced to 370 °C and 213 °C, respectively. In step 3, Zone 2 was cooled from 213 °C to 200 °C at a rate of 1 °C/min, after which the cap of the CVD was opened.” has been added in the Methods.

In addition, Fig. R3 has been added as Fig. S4 in the revised Supplementary Information.

3. Have the authors compared with optical and electrical properties of the large sheets with conventional small size 2D crystals? How's PL intensity, defect density, and carrier mobility different from those smaller crystals?

ANS: Thank you for the comment. We have compared our melt-grown $BA_2MAPb_2Br_7$ film with mechanically exfoliated $BA_2MAPb_2Br_7$ flake from bulk single crystals. To ensure a fair comparison, both samples have the same thickness of approximately 50 nm.

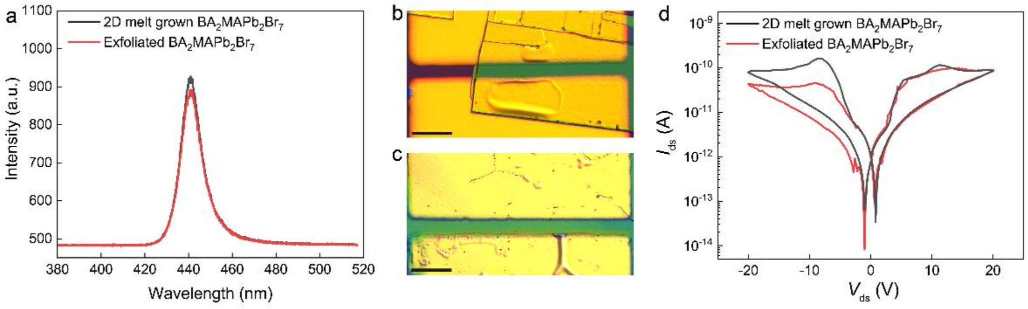

Fig. R4 Comparison of optical (**a**) and electrical properties (**b-d**) of $BA_2MAPb_2Br_7$ grown by

2D melt method with exfoliated $BA_2MAPb_2Br_7$ flake. Scale bar, 20 µm (**b**,**c**).

As shown in Fig. R4a, the PL intensity of the melt-grown film and the exfoliated small-size crystal are of the same order of magnitude, with comparable spectral linewidths (FWHM ≈ 10 nm), indicating that both samples possess high crystalline quality with similarly low defect densities.

We also performed a quantitative comparison of the I-V characteristics. Due to the small size of the exfoliated flakes, it is difficult to identify a uniform region that can accommodate a 110 µm width shadow mask for direct electrode deposition, in contrast to the melt-grown film (Fig. R4b,c). The measured I-V current of the grown film is slightly higher than that of the exfoliated flake (Fig. R4d). Since ferroelectric $BA_2MAPb_2Br_7$ is not a high-mobility material, reliable electrostatic gating is challenging, therefore, the carrier mobility cannot be accurately extracted at this stage. Ferroelectric hybrid perovskites are well suited for memristor applications in neuromorphic computing, which we will demonstrate in future work.

Reviewer #2 (Remarks to the Author):

The manuscript presents a two-dimensional melt growth method based on a NaxPbBry seeding layer. Using this method, large-area, thickness-controlled, single-crystalline HOIP films (n=1, 2) were synthesized on SiO_2/Si substrates. The manuscript concludes by demonstrating the successful implementation of these films in ferroelectric devices. This strategy ingeniously overcomes the long-standing challenges of substrate limitation and phase segregation in HOIP film growth. However, clarifications and careful revisions are needed before the manuscript can be considered for publication.

ANS: Thanks for your positive comments. We have added *in-situ* comparison growth videos of HOIP formation using the Na_xPbBr_y-BABr precursors versus conventional $PbBr_2$-BABr precursors recorded with a newly established CVD system equipped with optical microscopy (Micro-STS1200, Units Technology). These videos further corroborate our previous observations of melting, spreading into a 2D liquid film, and subsequent crystallization into single-crystalline films when using Na_xPbBr_y-BABr precursors (Videos 1–3).

1. The manuscript mentions that film thickness can be controlled by adjusting the distance between Zone 1 and Zone 2. Please elaborate on the underlying physical mechanism.

ANS: Thank you for the comment. As described in the Methods (Fig. R5), the distance between Zone 1 and Zone 2 (denoted as *D*) directly determines the amount of inorganic Na_xPbBr_y deposited on the SiO_2/Si. A shorter *D* leads to a higher flux and a higher accumulation of Na_xPbBr_y on the substrate, whereas increasing *D* reduces the deposited amount. As clearly shown in Figs. R5b-e, the amount of Na_xPbBr_y deposited on the SiO_2/Si substrate decreases systematically with increasing distance *D*. Furthermore, the melting of Na_xPbBr_y and BABr produces a 2D liquid film, and a larger amount of Na_xPbBr_y results in a higher density of the liquid phase on the substrate, which subsequently crystallizes into thicker films.

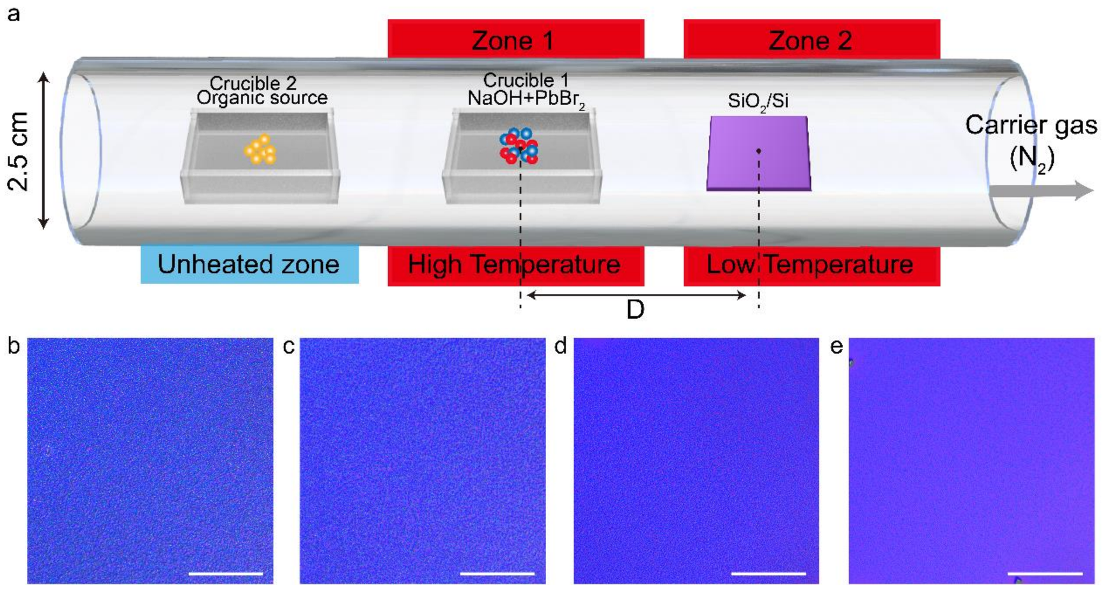

Fig. R5 **a**, Schematic illustration of 2D melt growth of HOIP films via CVD. **b-e**, Amount of deposited Na_xPbBr_y on SiO_2/Si as a function of distance (*D*): 16 cm (**b**), 17 cm (**c**), 18 cm (**d**) and 19 cm (**e**). Scale bars, 50 μm (**b-e**).

2. It is recommended to provide more direct experimental evidence or theoretical, computational support (e.g., contact angle simulations or interfacial energy calculations) in the main text or supplementary information to compare the wettability of the Na_xPbBr_y film versus $PbBr_2$.

ANS: Thank you for your comment. We have added new supplementary experiments to address this point: 1. Comparative contact-angle measurements of Na_xPbBr_y and $PbBr_2$ on SiO_2/Si; 2. Comparative *in-situ* video recordings of the interactions between Na_xPbBr_y and BABr, and between $PbBr_2$ and BABr, to evaluate the wettability of Na_xPbBr_y versus $PbBr_2$.

As shown in Fig. R6, the contact angle of $PbBr_2$ on SiO_2 is 112.59°, whereas Na_xPbBr_y is 35.09°, confirming the better wettability of Na_xPbBr_y compared with $PbBr_2$ on SiO_2. The improved wettability enables Na_xPbBr_y to form a highly dense layer cross the entire substrate, whereas $PbBr_2$ only forms microsized particles due to its poorer wetting property, consistent with the description in our manuscript. **Furthermore, our *in-situ* videos provide convincing visual proof that only Na_xPbBr_y-BABr precursor system can melt to form a 2D liquid film.**

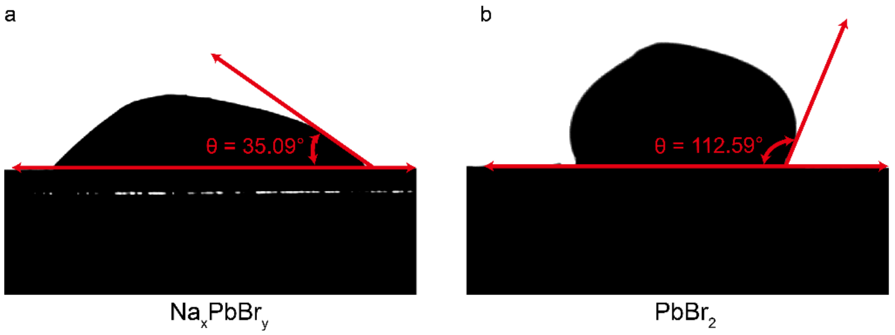

Fig.R6 Contact angle measurements of Na_xPbBr_y (**a**) and $PbBr_2$(**b**) on SiO_2/Si substrate.

Action: Fig. R6 has been added as Fig. S14 in the revised Supplementary Information.

3. Will the byproducts (NaBr, Pb(OH)$_2$) affect the growth of HOIP crystal? Where will these byproducts be located? Any evidence they are completely removed?

ANS: Thank you for your comment. The by-product Pb(OH)$_2$ further decomposes into PbO and remains as a residue in Crucible 1 (Fig. R7a). The initial reaction, $2\mathrm{NaOH} + 7\mathrm{PbBr_2} \rightarrow 2\mathrm{NaPb_3Br_7} + \mathrm{Pb(OH)_2}$ is carried out at 580 °C in Crucible 1. At this temperature, Pb(OH)$_2$ undergoes thermal decomposition according to $\mathrm{Pb(OH)_2}(s)\xrightarrow[400\ ^\circ\mathrm{C}]{\Delta}\mathrm{PbO}\ (s) + \mathrm{H_2O}\ (g)$. The formation of PbO was confirmed by XRD measurement of the residual powder collected from Crucible 1 after the initial reaction (Fig. R7b).

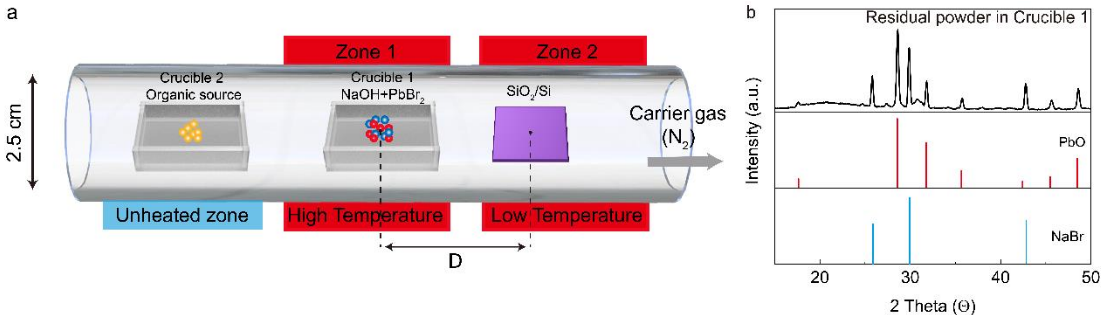

Fig. R7 **a**, CVD growth of HOIP film using $\mathrm{Na_xPbBr_y}$-BABr precursors. **b**, XRD measurement of the residual powder in Crucible 1.

The second interaction proceeds as $\mathrm{NaPb_3Br_7} + 6\mathrm{BABr} \rightarrow 3\mathrm{(BA)_2PbBr_4} + \mathrm{NaBr}$. The by-product NaBr segregated to the edges of the as-grown HOIP films. To determine its distribution, we performed XPS and TEM elemental mapping on as-grown HOIP films with a thickness of ~ 9 nm. Multiple XPS measurements (with an X-ray spot size of ~400 μm) taken from the central region of the film show no detectable Na signal (Fig. R8a,b). In contrast, a weak Na signal is observed at the film edge. Because XPS is a surface-sensitive technique (probing the top ~1–10 nm), we further carried out TEM elemental mapping on HOIP films directly grown on Cu/carbon TEM grids, which allows full transmission through the entire film thickness to bottom. As shown in Fig. R8c-h, no Na signal is detected within the mapped region, indicating that NaBr is not present at or beneath the bottom of the perovskite layer.

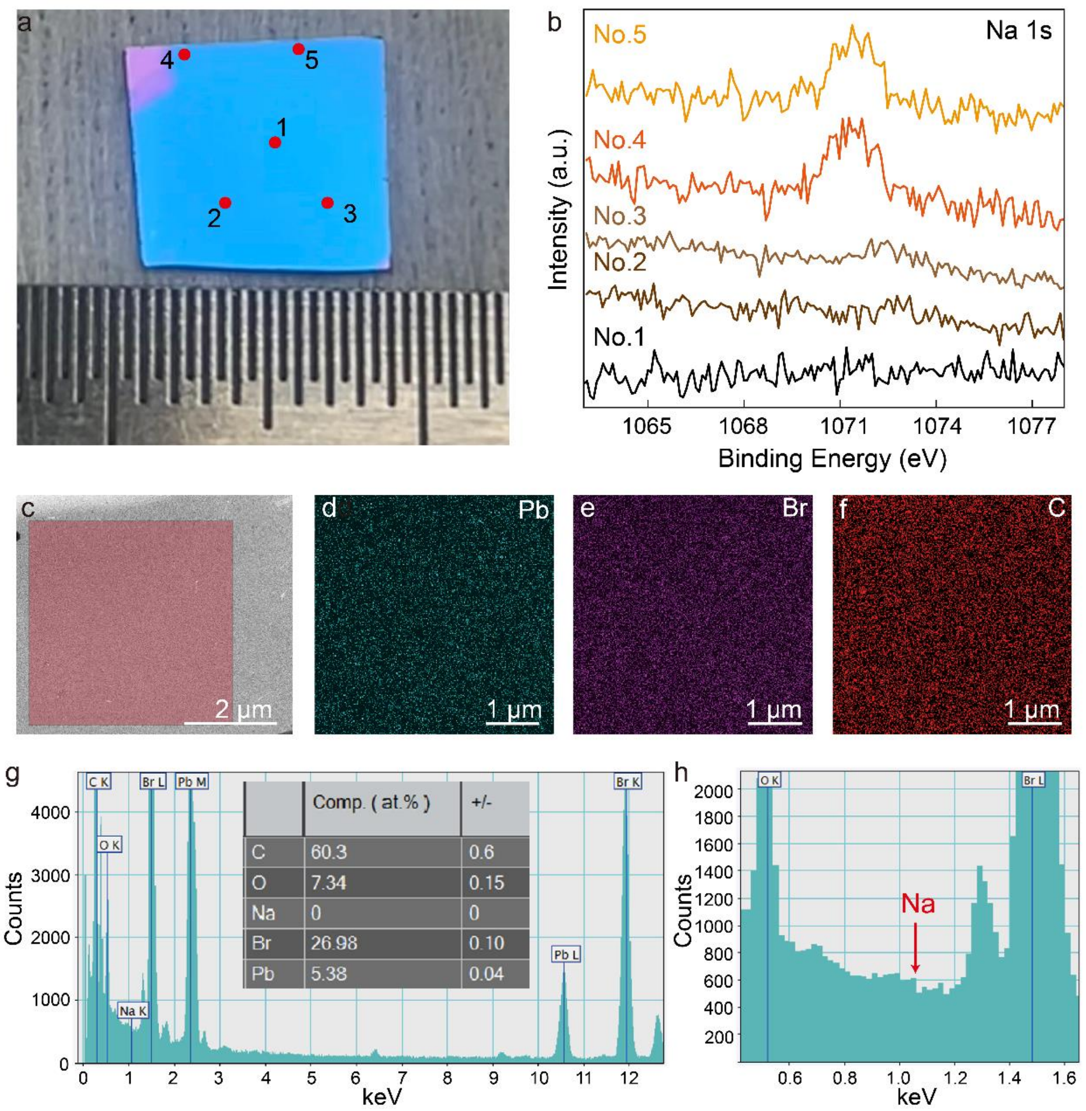

Fig. R8 **Characterization of NaBr by-product**. **a**, Photograph of n = 1 BA_2PbBr_4 single-crystalline film on SiO_2/Si. **b**, Corresponding Na 1s XPS spectra collected at five positions marked in (**a**). **c**, TEM image of BA_2PbBr_4 film grown on a TEM grid. **d-f**, Elemental maps of (**d**) Pb, (**e**) Br and (**f**) C acquired from the red rectangular region in (**c**). **g**, EDS spectrum acquired from the red rectangular region in (**c**). **h**, Zoom-in EDS spectrum on the Na-related energy range.

The NaBr-rich edge regions can be easily removed using a DMF-soaked cotton swab. In fact, the films shown in Fig. 1c and 1d in the manuscript correspond to the NaBr-edges have been removed. The film samples for GIXRD measurements shown in Fig. 2 were also the NaBr-edges removed.

Action: The following description has been added to page 9 of the revised manuscript: “It is worth noting that during the HOIP growth process, the by-product $Pb(OH)_2$ further decomposes into PbO in the crucible of the CVD furnace (Methods). Another by-product, NaBr, is primarily detected at the edges of the as-grown HOIP films (Figure S8) and can be readily removed using

a DMF-soaked cotton swab." In addition, Fig. R8 has been added as Fig. S8 in the revised Supplementary Information.

4. The X-axis (coordination number) range in Fig. 3j is only 0-5, yet the text (lines 234-238) discusses structures with coordination numbers of 6 (Na_xPbBr_y) and 7 ($PbBr_2$). Please verify and ensure consistency between the figure and the textual description.

ANS: Thank you for your comment. The X-axis in Fig. 3j represents the Pb–Br coordination number (CN) as determined by well-tempered metadynamics (WTmetaD) simulations. In this calculation, we identify the target octahedral structure through free-energy calculations of CN-dependent configurations, which form under increased Br^- coordination with Pb^{2+}.

The X-axis range is restricted to 0-5 because our WTmetaD sampling efficiently resolves configurations within this interval. When CN > 5 (as shown in Fig. R9), the free-energy profiles in both environments exhibit plateau-like behavior, indicating the presence of an inaccurate free energy barrier due to insufficient sampling in this region. That's why we only plotted CN from 0-5, in which the free-energy profile was adequately resolved.

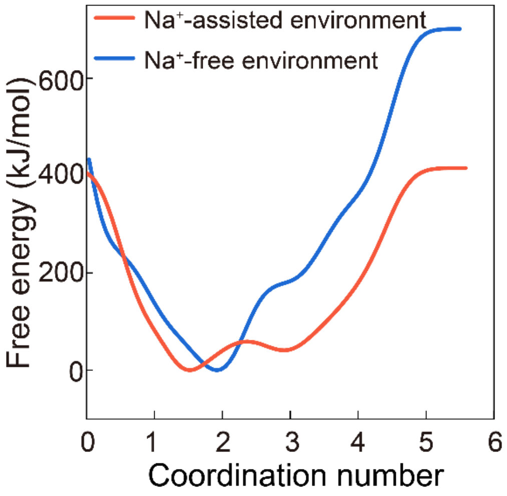

Fig R9. Free energy profiles include CN >5.

The text in lines 234–238 provides an alternative structural explanation for why a Na^+-assisted environment is more favorable for HOIP formation. As stated in the text: "*The 3D $PbBr_2$ crystallizes in the orthorhombic Pnma space group and adopts a cotunnite structure, where each Pb^{2+} is bonded in a 7-coordinate geometry to Br^-. Upon substitution of Na^+ in $PbBr_2$ and its subsequent elimination as NaBr, the concentration of Br^- available to coordinate with Pb^{2+} decreases, thereby promoting the formation of 6-coordinate $PbBr_6$ octahedra in HOIP.*" Together, the discussions in lines 232–234 and lines 234–238 provide two complementary explanations: energetic and structural–for why a Na^+-assisted environment is more favorable for producing HOIPs. To avoid confusion, we have added a transitional sentence between these two explanations (see Action below).

Action: The transitional sentence "Besides the free energy consideration, structural factors also provide an additional explanation for why the Na^+-assisted environment facilitates the

conversion to 6-coordinate $PbBr_6$ octahedra in HOIPs" has been added to page 13 of the revised manuscript. Fig. R9 has replaced the original Fig. 3j as the new Fig. 3j in the revised manuscript.

5. How were the optical images in Fig. 4a-c obtained during the high-temperature growth process? Furthermore, optical images alone are not conclusive evidence for a liquid state. Are there supplementary data confirming the transition of Na_xPbBr_y into a liquid phase at 210°C?

ANS: Figure 4a-c was obtained via a home-built temperature- and atmosphere- controlled heating stage (Linkam Thms600), combined with an optical microscope (Leica DMC5400). This setup was used to monitor the interaction of Na_xPbBr_y and BABr and the corresponding optical images were recorded as Fig. 4a-c.

In the revised manuscript, we have revised Fig. 4a-c with Fig. R10 to demonstrate the *in-situ* HOIP film growth process, extracted from **Videos 1. Videos 1** and **2** clearly capture the melting of Na_xPbBr_y and BABr, followed by crystallization into films with different thicknesses. In contrast, $PbBr_2$ and BABr do not exhibit a similar melt-mediated growth behavior (**Video 3**).

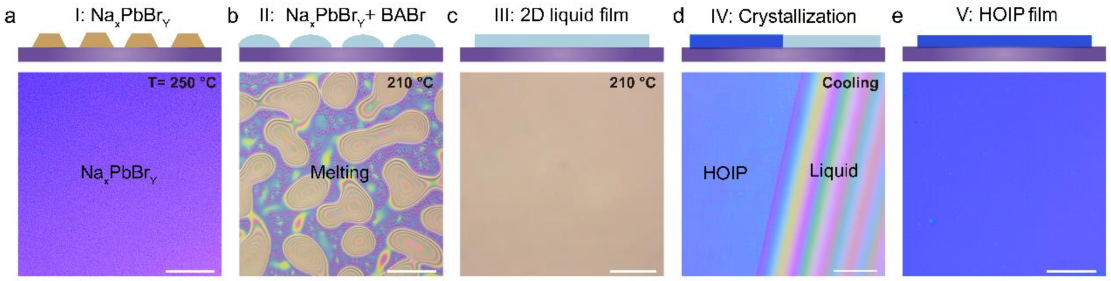

Fig. R10 *In-situ* optical images showing the 2D melt growth process of the HOIP film. Scale bars, 50 µm (**a**), 100 µm (**b-d**) and 25 µm (**e**).

In addition, we conducted differential scanning calorimetry (DSC) measurements, which are widely used to verify the melting behavior (*J. Am. Chem. Soc.* **146**, 9272–9284 (2024); *Nat. Commun.* **16**, 7696 (2025); *Nat. Chem.* **13**, 778–785 (2021)). As shown in Fig. R11, a clear endothermic peak corresponding to congruent melting of the Na_xPbBr_y and BABr is observed, yielding a melting temperature (T_m) of 199.8 °C, which is well below the decomposition temperature (T_d) of BA_2PbBr_4. This melting temperature closely matches the substrate temperature of 210 °C used in our growth process (Fig. R10c) within experimental error margin, confirming that the precursors are maintained in the liquid phase under this condition.

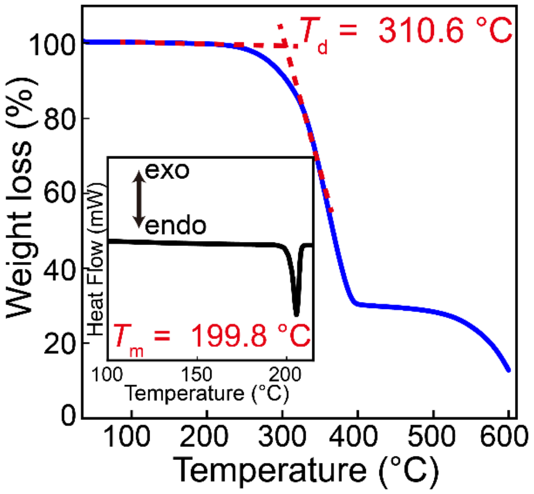

Fig. R11 TGA analysis of BA_2PbBr_4 and DSC curve of Na_xPbBr_y interacting with BABr (inset).

Action: Figure R10 replaces the original Fig. 4a-c as the new Fig. 4a-e in the revised manuscript. The description has been added on page 15 of the revised manuscript: "To further verify the melting behavior of the Na_xPbBr_y and BABr precursors, differential scanning calorimetry (DSC) measurements were performed. The DSC result reveals a melting temperature (T_m) of 199.8 °C, which is well below the decomposition temperature (T_d) of BA_2PbBr_4 (Fig. 4g and Figure S15). This melting temperature closely matches the substrate temperature of 210 °C used during growth within experimental error margin (Fig. 4c), thus confirming that the precursors are maintained in the liquid phase under this condition." In addition, Fig. R11 has been added as new Fig. 4g in the revised manuscript.

6. Please clarify in the caption of Fig. 4g whether the "Nucleation energy" and "Interfacial energy" profiles are qualitative schematic illustrations or quantitative results derived from theoretical calculations.

ANS: The "Nucleation energy" and "Interfacial energy" profiles shown in Fig. 4g are qualitative schematic illustrations, rather than quantitative results derived from theoretical calculations. The schematic illustration was inspired by the literature cited in the manuscript (*Nat. Mater.* **24**, 188-196 (2025)). In that work (see Fig. 2e, attached here as Fig. R12), the authors present qualitative energy diagrams comparing the nucleation and diffusion barriers during liquid-to-solid crystallization of MoS_2 with those in a conventional vapor-solid CVD process.

[Figure Redacted]

Fig. R12 Qualitative schematic illustration comparing the nucleation and diffusion barrier variations during MoS_2 growth via a liquid-to-solid CVD process and a conventional vapor-

solid CVD process. (Fig. R12 is cited from *Nat. Mater.* **24**, 188-196 (2025)).

Our system is conceptually similar to the scenario described in this *Nat. Mater.* paper. In our case, the formation of a liquid phase, as directly observed in the *in-situ* videos, promotes homogeneous nucleation compared to growth processes without a liquid intermediate. Furthermore, the rapid spreading of the liquid phase during film formation, also captured in the *in-situ* videos, effectively lowers the diffusion barrier. From a thermodynamic perspective, interfacial energy governs wetting behavior, with lower interfacial energy corresponding to higher wettability. Therefore, the qualitative comparison presented in Fig. 4g is reasonable and serves to illustrate the underlying physical mechanisms rather than to provide quantitative energy values.

Action: The figure caption of Fig. 4h has changed to "Qualitative comparison of nucleation energy and interfacial energy variation during HOIPs growth using Na_xPbBr_y (pink line) versus $PbBr_2$ (blue line)."

7. How was the cooling rate mentioned around line 270 precisely controlled? While the Methods section lists specific temperature programs and cooling rates, it is suggested to briefly explain the experimental basis or optimization process for selecting these particular parameters.

ANS: The cooling rate was precisely controlled using a programmable PID (Proportional–Integral–Derivative) controller integrated into the CVD furnace, which regulates the heating power to achieve a well-defined and reproducible cooling profile.

Regarding the selection and optimization of the temperature program and cooling rate, we take BA_2PbBr_4 as a representative example. Figure R13 shows a typical heating–cooling cycle applied to the Na_xPbBr_y-BABr precursors. The temperature T_m corresponds to the congruent melting process, while T_c represents the crystallization temperature at which BA_2PbBr_4 forms during cooling. Notably, the melting point of NaBr is approximately 747 °C; therefore, the observed endothermic peak cannot originate from NaBr.

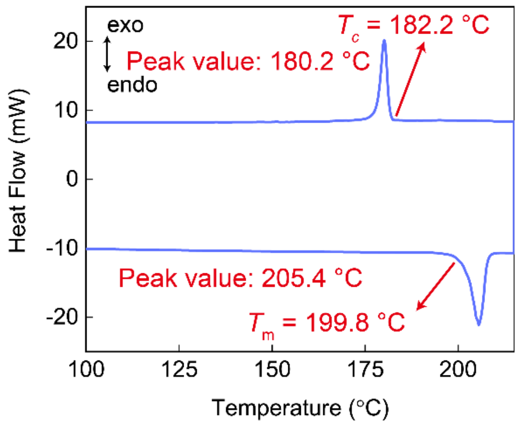

Fig. R13 Heating-cooling DSC scans of Na_xPbBr_y interacting with BABr.

Based on these thermal characteristics, the temperature of Zone 2 (the SiO_2/Si substrate

temperature) was set to 210 °C during the melting process, basically consistent with T_m, and reduced to 190 °C during the crystallization process, basically consistent with T_c. Because gas flow is present during the CVD growth process, slight temperature deviations from T_m and T_c are reasonable. The cooling rate from 210 °C to 190 °C was set to 1.0 °C min^{-1}. When faster cooling rates (>1.0 °C min^{-1}, e.g., 1.5 °C min^{-1}) were applied, dendritic structures formed, as shown in Video 4. Then, we reduced the cooling rate to 1.0 °C min^{-1} enabled the film formation. After 190 °C, since the crystallization has almost finished, the cap of the CVD system was opened, allowing to cool to room temperature under a continuous N_2 gas flow.

Action: The description "To further verify the melting behavior of the Na_xPbBr_y and BABr precursors, differential scanning calorimetry (DSC) measurements were performed. The DSC result reveals a melting temperature (T_m) of 199.8 °C, which is well below the decomposition temperature (T_d) of BA_2PbBr_4 (Fig. 4g and Figure S15). This melting temperature closely matches the substrate temperature of 210 °C used during growth within experimental error margin (Fig. 4c), thus confirming that the precursors are maintained in the liquid phase under this condition." has been added on page 15 of the revised manuscript. In Methods, the description "The heating and cooling scans of DSC measurement can be used to estimate the experimental melting temperature (210 °C) and crystallization temperature (190 °C) (Figure S15)" has been added on page 21 of the revised manuscript.

8. To better understand the "complete wetting" behavior, could the authors provide additional characterization data for the seeding layer itself (e.g., SEM, XPS, AFM surface morphology) to illustrate its uniformity, coverage, and bonding state with the substrate?

ANS: First, we would like to clarify that the term "complete wetting" was never used in the manuscript. The behavior referred to by the reviewer likely corresponds to the formation of a 2D liquid film during growth. Importantly, the Na_xPbBr_y seeding layer does not exhibit "complete wetting" on SiO_2/Si; rather, it shows improved wettability compared to $PbBr_2$, and the melting behavior occurs only upon interaction with BABr.

The enhanced wettability of Na_xPbBr_y relative to $PbBr_2$ has been verified by comparative *in-situ* video observations and contact-angle measurements (see response to Question 2). As requested, we additionally performed SEM and AFM measurements (Fig. R14). The SEM images clearly indicate that the Na_xPbBr_y forms a denser on SiO_2/Si compared to $PbBr_2$. Moreover, the morphology of Na_xPbBr_y evolves into a quasi-2D structure, in contrast to the 3D $PbBr_2$. Consistently, AFM images reveal a thinner, flake-like morphology for Na_xPbBr_y, further supporting its enhanced surface coverage relative to $PbBr_2$. The high dense and 2D structure of Na_xPbBr_y have already verified in our manuscript.

Regarding interfacial bonding, Na_xPbBr_y does not form chemical bonds with the SiO_2/Si substrate, instead, its adhesion is governed by electrostatic interactions. Therefore, XPS analysis is not expected to provide meaningful information on the wettability of Na_xPbBr_y and would deviate from the main focus of the manuscript.

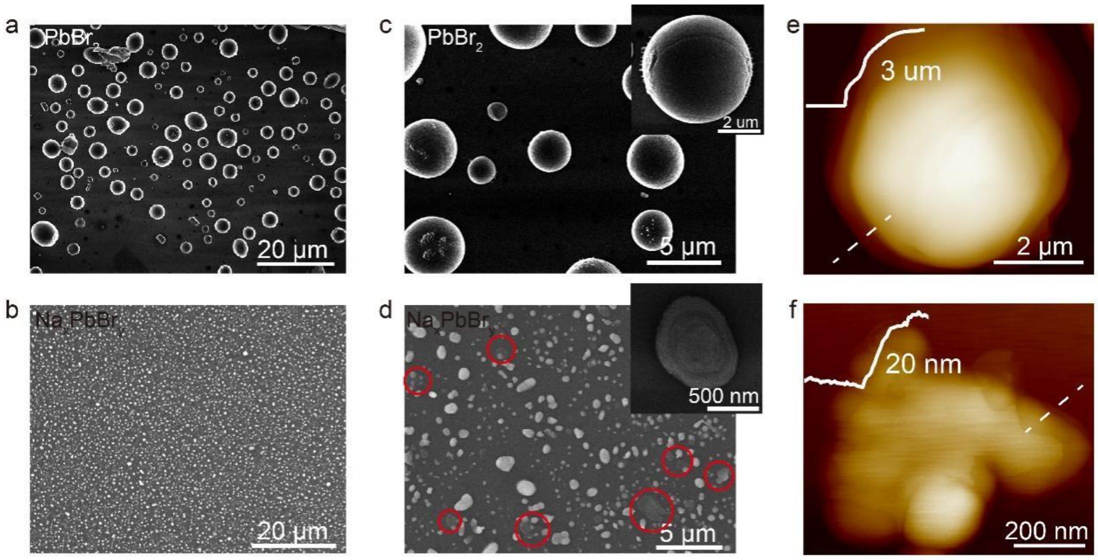

Fig. R14 **Characterization of Na_xPbBr_y seeding layer and $PbBr_2$ layer**. **a**,**b,** Comparative SEM images of $PbBr_2$ (**a**) and Na_xPbBr_y (**b**). **c**,**d,** Enlarged SEM images of $PbBr_2$ (**c**) and Na_xPbBr_y (**d**). **e**,**f,** Comparative AFM images of $PbBr_2$ (**e**) and Na_xPbBr_y (**f**).

Action: The description "AFM and SEM characterizations further confirm the high density and 2D morphology of Na_xPbBr_y as compared to $PbBr_2$ (Figure S12)." has been added on page 11 of the revised manuscript. Fig. R14 has been added as Fig. S12 in the revised Supplementary Information.

9. The feasibility of growing BA/HA-based 2D single crystal films with n=3 and n=4 should be explored, which would help to demonstrate the broader applicability of the growth technique.

ANS: Thank you for your suggestion. In the revised manuscript, we demonstrate the growth of additional BA/HA-based 2D HOIP single-crystal films, including $BA_2MA_2Pb_3Br_{10}$ ($n = 3$) (Fig. R15a,b) and $HA_2MAPb_2Br_7$ ($n = 2$) (Fig. R15c,d), as well as Pb-I-based BA_2PbI_4 ($n = 1$) (Fig. R3), following Reviewer 1's suggestion. Together with previously reported films, we have now grown six HOIP films, demonstrating the broad applicability of the 2D melt-growth mechanism.

Although $n = 4$ growth is not included, the successful formation of $n = 2$ and $n = 3$ BA-based HOIP films confirms that the Na_xPbBr_y-BABr-MABr precursor system can undergo congruent melting and spread into a two-dimensional liquid film for $n > 1$ HOIPs. Extension to $n = 4$ BA-based and $n = 3$ and $n = 4$ HA-based HOIPs is expected through precursor-ratio optimization and will be reported in our future work. Relevant growth methods of $BA_2MA_2Pb_3Br_{10}$ ($n = 3$) and $HA_2MAPb_2Br_7$ ($n = 2$) have been added to the revised manuscript.

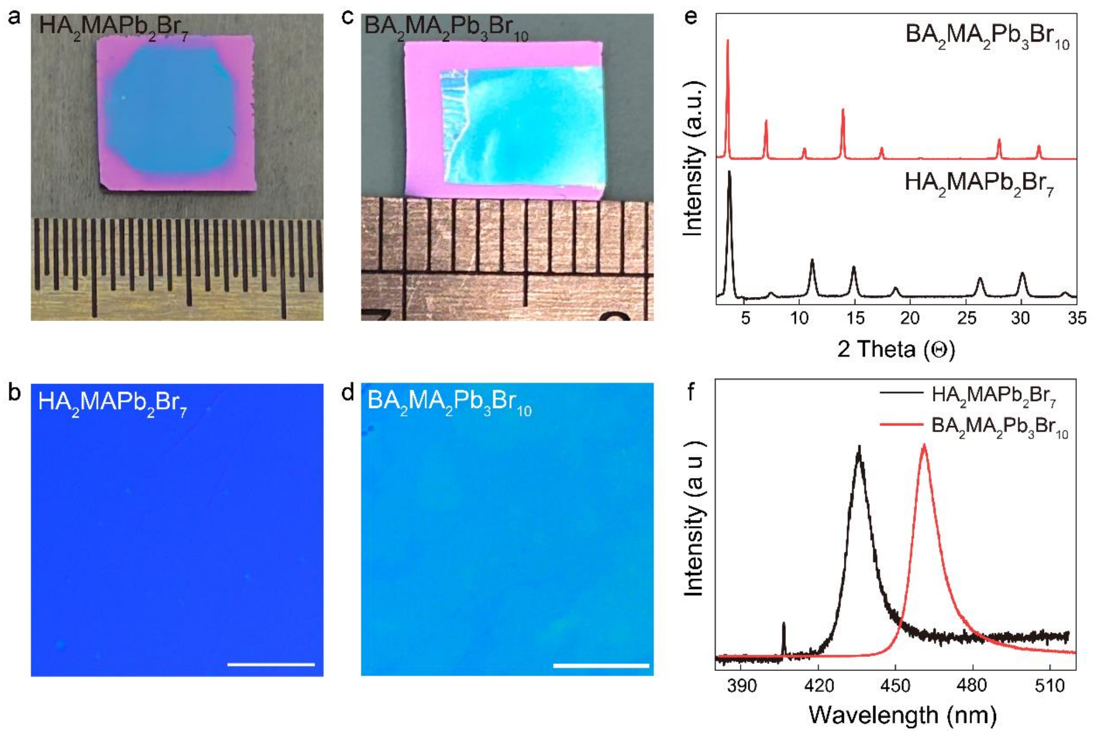

Fig. R15 Photographs and optical images of $HA_2MAPb_2Br_7$ (n = 2) (**a**,**b**) and $BA_2MA_2Pb_3Br_{10}$ (n = 3) (**b**,**d**) films. XRD patterns (**e**) and PL (**f**) characterization of the $HA_2MAPb_2Br_7$ (n = 2) and $BA_2MA_2Pb_3Br_{10}$ (n = 3) film. Scale bars, 25 μm (**b**), 50 μm (**d**).

Action: Relevant growth methods:

“2D melt growth of centimetre-scale *n* = 3 $(BA)_2(MA)_2Pb_3Br_{10}$ film

For the growth of n = 3 $(BA)_2(MA)_2Pb_3Br_{10}$, the entire process was conducted under N_2 gas (99.9%) with a flow rate of 80 sccm. The distance D was set to 18 cm. The organic source was modified to a mixture of BABr (31 mg) and MABr (22 mg). Step 1 is identical to the procedure used for the growth of n = 1 $(BA)_2PbBr_4$. In step 2, the temperature of Zone 2 was reduced to 175 °C. In step 3, the temperature of Zone 2 was allowed to cool naturally from 175 °C to 150 ºC, after which the cap of the CVD system was opened.”

“2D melt growth of centimetre-scale *n* = 2 $(HA)_2(MA)Pb_2Br_7$ film

For the growth of n = 2 $(HA)_2(MA)Pb_2Br_7$, the entire process was conducted under N_2 gas (99.9%) with a flow rate of 80 sccm. The distance D was set to 17 cm. The organic source was modified to a mixture of HABr (36 mg) and MABr (11 mg). Step 1 is identical to the procedure used for the growth of $(BA)_2PbBr_4$ (n = 1). In step 2, the temperature of Zone 2 was reduced to 220 °C. In step 3, the cooling rate was set to 2.2 °C/min, cooling the temperature of Zone 2 from 220 °C to 200 °C.”

The description “Additionally, we also grew $BA_2MA_2Pb_3Br_{10}$ (n = 3), $(HA)_2PbBr_4$ (n = 1), $HA_2MAPb_2Br_7$ (n = 2) and Pb-I based BA_2PbI_4 (n = 1) large-scale films on SiO_2/Si, as shown in Figure S3-6. X-ray diffraction (XRD) patterns of six different HOIPs in Fig. 1j indicates the growth of highly oriented (001)-textured films.” has been added on page 8 of the revised manuscript. Fig. R15 has been added as Fig. S5 and Fig. S6 in the revised Supplementary

Information.

10. In the Conclusion, based on the successful (BA/HA)-based bromide systems, it would be beneficial to more specifically discuss the potential challenges in extending this method to other HOIP systems (e.g., iodides, Dion-Jacobson phases). A brief discussion on the current limits, precision of thickness control, and potential future improvements would also be valuable.

ANS: Besides BA/HA based HOIPs, aromatic cation based HOIPs can also be grown using our 2D melt growth method. The concept of "melt state" of HOIP has recently been reported (*Nat. Rev. Mater*. **10**, 211-227 (2025); *J. Am. Chem. Soc*. **146**, 9272-9284 (2024)). Notably, congruent melting temperature (T_m) has been reported for chiral *S-/R*-$(NEA)_2PbBr_4$ (T_m = 175 °C) (*Nat. Commun*. **16**, 7696, (2025)), as well as for several aromatic bulky-cation-based HOIPs, including β-Me-PEA_2PbI_4 (T_m = 207 °C); 2-F-PEA_2PbI_4 (T_m = 245.8 °C); 4-MeO-PEA_2PbI_4 (T_m = 247.1 °C); PEA_2PbI_4 (T_m = 252.9 °C) (*Chem. Mater.* **29**, 6200–6204 (2017)). These compounds therefore exhibit strong potential for 2D melt growth into large-scale films, provided that suitable growth parameters are established to ensure uniform substrate wetting and stable crystallization. It should be noted that not all HOIP precursors can form stable molten states. At present, melting behavior has not been reported for diammonium-cation-based Dion–Jacobson HOIPs, leaving significant opportunity to first explore their melt states and subsequently extend the 2D melt-growth strategy to these systems.

Beyond CVD, organic molecular beam epitaxy (OMBE) represents a highly promising alternative, offering wafer-scale growth with monolayer-level thickness control for HOIP films grown via the 2D melt-growth approach.

We have added a new discussion regarding these points in the revised manuscript, as shown in Action below.

Action: The conclusion of the manuscript has been revised as follows: "In addition to HOIPs based on aliphatic chain and cyclic organic cations, HOIPs incorporating aromatic cations can also be grown using our 2D melt growth approach. Moreover, congruent melting has been reported for chiral NEA-based HOIPs, halogen substituted PEA-based HOIPs, and even 3D perovskites[43-45]. These findings highlight strong potential for extending the 2D melt growth strategy to these systems. Compared to furnace-type CVD, organic molecular beam epitaxy (OMBE) provides a more tunable growth method, affording wafer scale growth with precise thickness control. Bypassing substrate limitations and enabling parallel production, this work provides a foundation for expanding HOIP-based devices into large-scale industrial applications, including integrated HOIP/Si photovoltaics, photonics, and neuromorphic computing platforms."

A Point-by-Point Response Letter for Nature Communications Manuscript NCOMMS-25-78861A "*Two-dimensional melt growth of large-scale, single-crystalline hybrid organic-inorganic perovskite films*"

Reviewer #1 (Remarks to the Author):

Authors have carefully revised the manuscript based on reviewers' feedback. The quality and clarity of the manuscript have been greatly improved. I want to congratulate the authors for the impressive results. In my opinion this paper can be accepted and published now.

Reviewer #2 (Remarks to the Author):

The authors have performed additional experiments, particularly the in-situ optical microscopy, DSC measurements, which have substantially strengthened the manuscript. However, after carefully examining the revised materials, two issues remain that require further clarification.

1. The authors provide contact angle measurements to demonstrate the superior wettability of Na_xPbBr_y (35.09°) compared to $PbBr_2$ (112.59°). However, the droplets appear highly irregular in shape, and the contact angle is clearly asymmetric: the left side exhibits a visibly larger angle than the right side. The reported value of 35.09° was taken from the right side, which shows a smaller angle. This selective reporting is problematic, as the true contact angle for an irregular droplet is ambiguous. The authors should provide additional contact angle measurements (at least 3–5 independent droplets) to ensure reliability. Alternatively, if the irregularity stems from the measurement technique, a more controlled method should be considered.

ANS: We have provided a video of the contact angle measurement of Na_xPbBr_y to demonstrate its wetting behavior on SiO_2 substrate for the reviewer's reference. Na_xPbBr_y exhibits clear and favorable wetting on SiO_2 surface. Fig. R1 shows a symmetric contact angle of 23°, we extracted from the video at 20 seconds, highlighting its excellent wettability. For comparison, we also supplied a video of the contact angle measurement of $PbBr_2$ on SiO_2. In contrast to Na_xPbBr_y, $PbBr_2$ maintains a contact angle of 112° (> 90°) without further spreading, indicating poorer wetting behavior.

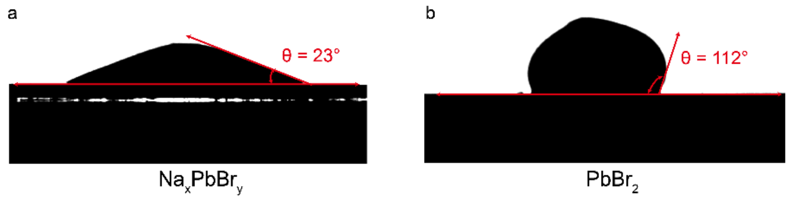

Fig. R1 Contact angle of Na_xPbBr_y (a) and $PbBr_2$ (b) on SiO_2/Si substrate.

These comparative results sufficiently support the statement regarding the "poorer wetting property of $PbBr_2$ on SiO_2/Si (Supplementary Fig. 14)" in our manuscript.

Action: We have replaced Supplementary Fig. 14 with the updated Fig. R1 shown above in the Supplementary Information.

2. The authors successfully grew $(BA)_2(MA)_2Pb_3Br_{10}$ (n = 3) and $(HA)_2(MA)Pb_2Br_7$ (n = 2), which indeed extends the method's demonstrated versatility. A key claim of the paper is the broad applicability of the growth technique, and the absence of n = 4 leaves this claim partially unsubstantiated. The growth of an n = 4 film (BA-based) is strongly encouraged. A successful demonstration, even with non-optimized film quality, would significantly strengthen the claim of broad applicability. If the attempt fails, a brief discussion of the observed challenges would still be valuable for the community.

ANS: Thank you for your suggestion. The absence of $(BA)_2(MA)_3Pb_4Br_{13}$ (n = 4) single-crystalline film is not due to a limitation of our growth technique, but rather to the intrinsic difficulty in obtaining phase-pure $(BA)_2(MA)_3Pb_4Br_{13}$ n = 4 (Br-based). I-based n = 4 can be grown; however, the I-based series does not exhibit ferroelectricity; thus, $(BA)_2(MA)_3Pb_4I_{13}$ is not relevant to the focus of our work and could introduce conceptual inconsistency.

As shown in Fig. R2 and reported in our previous work (Kai Leng*, *Nat. Commun.* **15**, 10221, (2024)), we successfully synthesized bulk single crystals of $(BA)_2(MA)_{n-1}Pb_nBr_{3n+1}$ for n = 1, 2, and 3 using solution methods. However, when attempting to grow $(BA)_2(MA)_3Pb_4Br_{13}$ (n = 4), lower-n phases and 3D perovskite impurities readily form due to the intrinsic thermodynamic instability of the $(BA)_2(MA)_3Pb_4Br_{13}$ (n = 4). To date, to the best of our knowledge, there have been no reports demonstrating the successful growth of phase-pure $(BA)_2(MA)_3Pb_4Br_{13}$ (n = 4) single crystal. If bulk single crystal cannot be achieved, it can be appreciated that it will be challenging to realize large-scale single-crystalline thin films of this material by the current growth method.

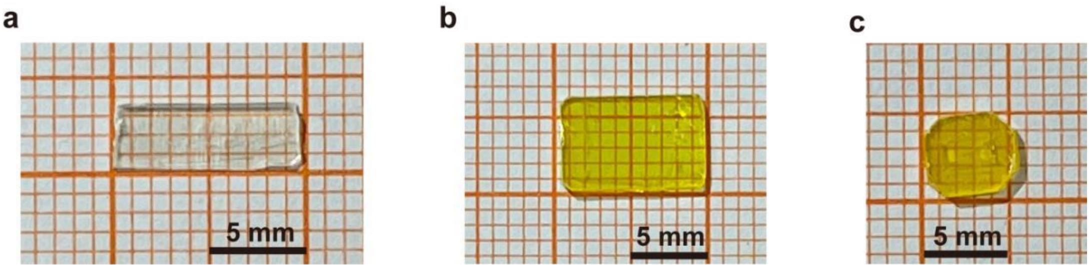

Fig. R2 Photographs of $(BA)_2(MA)_{n-1}Pb_nBr_{3n+1}$ (n = 1-3) single crystals. n = 1 (a), b, n = 2 (b) and n = 3 (c). (Cited from *Nat. Commun.* **15**, 10221, (2024))

As shown in Fig. R3, we have also attempted 2D melt growth of the $(BA)_2(MA)_3Pb_4Br_{13}$ (n = 4). The results indicate that morphologically uniform film can be obtained, and a single

photoluminescence (PL) emission peak at 476 nm—characteristic of the $n = 4$ phase—is observed. However, XRD measurements reveal that the presence of mixed phases, including $n = 2$, $n = 3$, and 3D perovskite phases, therefore we prefer not to include these data.

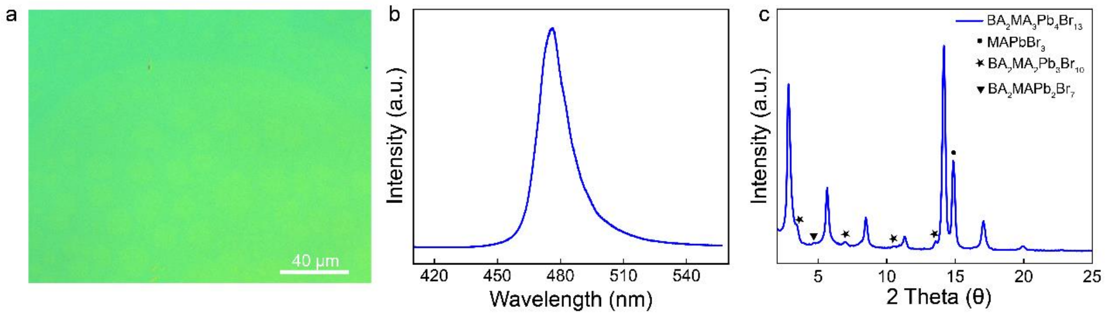

Fig. R3 Optical image (a), PL emission spectra (b) and XRD pattern (c) of the as-grown $(BA)_2(MA)_3Pb_4Br_{13}$ ($n = 4$) film.

We have summarized the previously reported studies on $(BA)_2(MA)_3Pb_4Br_{13}$ ($n = 4$) growth below (Table R1). To our knowledge, no prior work has reported the successful synthesis of $(BA)_2(MA)_3Pb_4Br_{13}$ ($n = 4$) single crystal.

Table R1. Summary of previous studies on $(BA)_2(MA)_3Pb_4Br_{13}$ ($n = 4$).

References	Characterization method			Note
	Single crystal XRD	PL	Powder XRD	
Adv. Funct. Mater. **33**, 2209249 (2023)	×	Single PL emission peak observed; however, the wavelength is inconsistent with other reports	Peaks assigned to 3D $MAPbBr_3$ detected	Spin-coated film
Adv. Funct. Mater. **35**, 2510543 (2025)	×	Two PL emission peaks observed	×	Spin-coated film

In summary, the challenge arises from the intrinsic phase instability of the Br-based $n = 4$ rather than from limitations of our 2D melt-growth strategy. Claiming the successful growth of phase-pure $(BA)_2(MA)_3Pb_4Br_{13}$ ($n = 4$) single-crystalline films without definitive evidence would raise further questions in the future.

Action: Since the difficulty does not arise from 2D melt-growth technique in obtaining $(BA)_2(MA)_3Pb_4Br_{13}$ ($n = 4$) films, we prefer not to be drawn to discuss $n = 4$ Br-based film in the present manuscript because we would like to leave this as an open question. As the response letter will be published alongside the paper, readers with specific interest in this issue will be able to refer to our detailed explanation here.

If the phase-purity issue of $(BA)_2(MA)_3Pb_4Br_{13}$ can be resolved in the future, then 2D melt-

growth strategy should be applicable for growing the single-crystalline films. We sincerely thank the reviewer for understanding our concern.

Point-by-Point Response Letter for Nature Communications Manuscript NCOMMS-25-78861B "*Two-dimensional melt growth of large-scale, single-crystalline hybrid organic-inorganic perovskite films***"**

Reviewer #2 (Remarks to the Author):

The authors have addressed all my concerns, and the paper can be accepted.

ANS: Thank you.
